# Assessment of Discrepancies Between Follow-up Infarct Volume and 90-Day Outcomes Among Patients With Ischemic Stroke Who Received Endovascular Therapy

**DOI:** 10.1001/jamanetworkopen.2021.32376

**Published:** 2021-11-05

**Authors:** Aravind Ganesh, Johanna M. Ospel, Bijoy K. Menon, Andrew M. Demchuk, Ryan A. McTaggart, Raul G. Nogueira, Alexandre Y. Poppe, Mohammed A. Almekhlafi, Ricardo A. Hanel, Götz Thomalla, Staffan Holmin, Volker Puetz, Brian A. van Adel, Jason W. Tarpley, Michael Tymianski, Michael D. Hill, Mayank Goyal

**Affiliations:** 1 Calgary Stroke Program, Department of Clinical Neurosciences, University of Calgary, Calgary, Alberta, Canada; 2 Department of Radiology, University Hospital Basel, University of Basel, Basel, Switzerland; 3 Department of Radiology, University of Calgary, Calgary, Alberta, Canada; 4 Department of Community Health Sciences, University of Calgary, Calgary, Alberta, Canada; 5 The Hotchkiss Brain Institute, University of Calgary, Calgary, Alberta, Canada; 6 Departments of Diagnostic Imaging, Neurology, and Neurosurgery, The Warren Alpert Medical School of Brown University, Providence, Rhode Island; 7 Departments of Neurology, Neurosurgery, and Radiology, Emory University School of Medicine, Atlanta, Georgia; 8 Neuroendovascular Service, Marcus Stroke and Neuroscience Center, Grady Memorial Hospital, Atlanta, Georgia; 9 Department of Neurosciences, Centre Hospitalier de l’Université de Montréal (CHUM), Université de Montréal, Montreal, Quebec, Canada; 10 Lyerly Neurosurgery, Baptist Hospital, Jacksonville, Florida; 11 Departments of Neurology and Neuroradiology, University Medical Center Hamburg-Eppendorf, Hamburg, Germany; 12 Department of Clinical Neuroscience, Karolinska Institutet and Departments of Neuroradiology and Neurology, Karolinska University Hospital, Stockholm, Sweden; 13 Dresden Neurovascular Center, Department of Neurology, University Hospital Carl Gustav Carus at the Technische Universität Dresden, Dresden, Germany; 14 McMaster University, Hamilton, Ontario, Canada; 15 Providence Little Company of Mary Medical Center, Providence Saint John’s Health Center and The Pacific Neuroscience Institute, Torrance, California; 16 Division of Neurosurgery and Neurovascular Therapeutics Program, University Health Network, Departments of Surgery and Physiology, University of Toronto, Toronto Western Hospital Research Institute, Toronto, Canada; 17 NoNO Inc, Toronto, Ontario, Canada; 18 Department of Medicine, University of Calgary Cumming School of Medicine, Calgary, Alberta, Canada

## Abstract

**Importance:**

Some patients have poor outcomes despite small infarcts after endovascular therapy (EVT), while others with large infarcts do well. Understanding why these discrepancies occur may help to optimize EVT outcomes.

**Objective:**

To validate exploratory findings from the Endovascular Treatment for Small Core and Anterior Circulation Proximal Occlusion with Emphasis on Minimizing CT to Recanalization Times (ESCAPE) trial regarding pretreatment, treatment-related, and posttreatment factors associated with discrepancies between follow-up infarct volume (FIV) and 90-day functional outcome.

**Design, Setting, and Participants:**

This cohort study is a post hoc analysis of the Safety and Efficacy of Nerinetide in Subjects Undergoing Endovascular Thrombectomy for Stroke (ESCAPE-NA1) trial, a double-blind, randomized, placebo-controlled, international, multicenter trial conducted from March 2017 to August 2019. Patients who participated in ESCAPE-NA1 and had available 90-day modified Rankin Scale (mRS) scores and 24-hour to 48-hour posttreatment follow-up parenchymal imaging were included.

**Exposures:**

Small FIV (volume ≤25th percentile) and large FIV (volume ≥75th percentile) on 24-hour computed tomography/magnetic resonance imaging. Baseline factors, outcomes, treatments, and poststroke serious adverse events (SAEs) were compared between discrepant cases (ie, patients with 90-day mRS score ≥3 despite small FIV or those with mRS scores ≤2 despite large FIV) and nondiscrepant cases.

**Main Outcomes and Measures:**

Area under the curve (AUC) and goodness of fit of prespecified logistic models, including pretreatment (eg, age, cancer, vascular risk factors) and treatment-related and posttreatment (eg, SAEs) factors, were compared with stepwise regression–derived models for ability to identify small FIV with higher mRS score and large FIV with lower mRS score.

**Results:**

Among 1091 patients (median [IQR] age, 70.8 [60.8-79.8] years; 549 [49.7%] women; median [IQR] FIV, 24.9 mL [6.6-92.2 mL]), 42 of 287 patients (14.6%) with FIV of 7 mL or less (ie, ≤25th percentile) had an mRS score of at least 3; 65 of 275 patients (23.6%) with FIV of 92 mL or greater (ie, ≥75th percentile) had an mRS score of 2 or less. Prespecified models of pretreatment factors (ie, age, cancer, vascular risk factors) associated with low FIV and higher mRS score performed similarly to models selected by stepwise regression (AUC, 0.92 [95% CI, 0.89-0.95] vs 0.93 [95% CI, 0.90-0.95]; *P* = .42). SAEs, specifically infarct in new territory, recurrent stroke, pneumonia, and congestive heart failure, were associated with low FIV and higher mRS scores; stepwise models also identified 24-hour hemoglobin as treatment-related/posttreatment factor (AUC, 0.92 [95% CI, 0.90-0.95] vs 0.94 [95% CI, 0.91-0.96]; *P* = .14). Younger age was associated with high FIV and lower mRS score; stepwise models identified absence of diabetes and higher baseline hemoglobin as additional pretreatment factors (AUC, 0.76 [95% CI, 0.70-0.82] vs 0.77 [95% CI, 0.71-0.83]; *P* = .82). Absence of SAEs, especially stroke progression, symptomatic intracerebral hemorrhage, and pneumonia, was associated with high FIV and lower mRS score2; stepwise models also identified 24-hour hemoglobin level, glucose, and diastolic blood pressure as posttreatment factors associated with discrepant cases (AUC, 0.80 [95% CI, 0.74-0.87] vs 0.79 [95% CI, 0.72-0.86]; *P* = .92).

**Conclusions and Relevance:**

In this study, discrepancies between functional outcome and post-EVT infarct volume were associated with differences in pretreatment factors, such as age and comorbidities, and posttreatment complications related to index stroke evolution, secondary prevention, and quality of stroke unit care. Besides preventing such complications, optimization of blood pressure, glucose levels, and hemoglobin levels are potentially modifiable factors meriting further study.

## Key Points

**Question:**

Why do some patients have poor outcomes despite small infarcts after endovascular therapy, while others with large infarcts fare better?

**Findings:**

In this cohort study of 1091 patients who received endovascular therapy as part of a randomized clinical trial, discrepancies between infarct volume and functional outcome were associated with prespecified pretreatment factors, such as age, cancer, and vascular risk factors, as well as posttreatment factors, such as infarct in new territory, stroke progression, intracerebral hemorrhage, recurrent stroke, pneumonia, and heart failure. Models including these factors performed similar to those derived from stepwise regressions.

**Meaning:**

In this study, discrepancies between outcome and infarct volume were associated with pretreatment and posttreatment factors, including complications related to index stroke evolution, secondary prevention, and quality of stroke unit care.

## Teaser

This cohort study validates exploratory findings from the Endovascular Treatment for Small Core and Anterior Circulation Proximal Occlusion with Emphasis on Minimizing CT to Recanalization Times (ESCAPE) trial regarding pretreatment, treatment, and posttreatment factors associated with discrepancies between follow-up infarct volume and 90-day functional outcome.

## Introduction

Endovascular thrombectomy (EVT) improves functional outcomes in patients with acute ischemic stroke caused by large-vessel occlusions (LVO),^1^ partly by reducing infarct volumes.^2,3,4^ Follow-up or final imaging infarct volume (FIV), measured 24 to 48 hours post stroke, is associated with functional outcomes at 90 days assessed using the modified Rankin Scale (mRS) in patients with stroke due to LVO.^5,6,7^ However, there is only a moderate correlation of FIV to clinical outcomes,^8,9^ with FIV only explaining an estimated 12% of the variance in EVT treatment benefit using mediation analyses.^10^ Similarly, infarct volume was a modest predictor of 3-month mRS score and a poor predictor of health-related quality-of-life in a prospective cohort.^11^ Some patients have discrepancies between FIV and functional outcome: patients with small FIV can fare poorly while others with large FIV can fare well.

Several explanations have been proposed for discrepant outcomes, including errors in measuring FIV or ascertaining outcome (pseudodiscrepancy); pretreatment factors, such as age, comorbidities, functional eloquence, and selective neuronal loss vs pan-necrosis of involved regions^12^; or posttreatment factors, such as differences in postacute care or complications including hemorrhage or pneumonia.^13,14,15^ In a recent exploratory analysis of the Endovascular Treatment for Small Core and Anterior Circulation Proximal Occlusion with Emphasis on Minimizing CT to Recanalization Times (ESCAPE) trial among 315 individuals, we found that discrepancies between FIV and 90-day mRS score occurred in 1 in 8 patients and were not associated with differences in imaging modality for FIV ascertainment or right vs left hemisphere involvement.^16^ Pretreatment factors best associated with small FIV and an mRS score of 3 or greater included older age, preexisting cancer, and vascular risk factors; posttreatment factors included 48-hour National Institutes of Health Stroke Score (NIHSS) and poststroke complications. Factors associated with large FIV and mRS score of 2 or less included absence of vascular risk factors, lower 24-hour NIHSS, and absence of complications. However, this analysis was exploratory or hypothesis generating, and the models included a mix of patients with and without EVT. Therefore, we sought to validate these exploratory findings by examining the performance of these models for FIV-mRS discrepancies in the larger Safety and Efficacy of Nerinetide in Subjects Undergoing Endovascular Thrombectomy for Stroke (ESCAPE-NA1) randomized clinical trial, in which all patients underwent EVT. We aimed to examine the construct validity of the discordance between infarct volume and outcome in patients who received EVT as well as the predictive validity of the identified pretreatment and posttreatment factors associated with discrepant outcomes at 90 days.

## Methods

ESCAPE-NA1 was a double-blind, randomized, placebo-controlled clinical trial (NCT02930018) involving 48 acute care hospitals in 8 countries.^17^ The trial enrolled patients with acute ischemic stroke due to LVO within 12 hours from last known well. Eligible adult patients had a disabling ischemic stroke, were functionally independent before their stroke, and met imaging criteria. Patients were randomly assigned to intravenous nerinetide or saline placebo, and unlike the ESCAPE trial, all received EVT. Intravenous alteplase was used according to best standard of care. Participants were enrolled from March 1, 2017, through August 12, 2019, and were followed up for 90 days.

The ethics board at each site approved the trial. A previous publicaton^18^ includes the study flow diagram and protocol. Signed informed consent was obtained from patients, authorized representatives, or if permitted by applicable national regulations, from the investigator with an independent physician not otherwise participating in the trial. The ESCAPE-NA1 data are not currently publicly available for distribution, but a future public data set may be made available.

All participants had follow-up imaging (magnetic resonance imaging [MRI] or computed tomography [CT]) at 24 hours from symptom onset. MR diffusion-weighted imaging (DWI) was the modality of choice for measuring FIV, but if it was not available or feasible because of clinical contraindications (ie, patient tolerability or instability), noncontrast CT was used. MRI at minimum included axial DWI, fluid-attenuated inversion recovery, and T2-weighted sequences, and CT included axial 5 mm cuts. Two experts (J.M.O. and M.G.) used the open-source software ITK Snap^19^ to measure the FIV (in milliliters) by manual planimetry on axial images while masked to all other clinical and imaging information. If the infarct showed hemorrhagic conversion, the hemorrhage regions were incorporated within the infarct boundaries. Masslike hemorrhages were included in segmentation if they were within or adjacent to infarcted areas, while hemorrhages remote from infarcted areas were not included.

All participants had standard assessments of demographic characteristics, medical history, laboratory values, and stroke severity (NIHSS). The primary outcome (ie, 90-day mRS score^20^) was assessed by trained personnel unaware of treatment-group assignment. Secondary clinical outcomes included 24-hour and 48-hour NIHSS and 30-day and 90-day Barthel Index (BI) scores.^21^ Serious adverse events (SAEs) were systematically monitored and reported, and included various events that were broadly categorized as being related to the index stroke, to EVT and/or thrombolysis, to secondary prevention, to stroke unit care, or as miscellaneous. Infarct in new territory (INT) was identified on 24-hour follow-up imaging and was defined as a new CT/DWI infarct (ie, not delineated on preprocedural noninvasive imaging) outside the affected territory (every territory not located distal to the clot).^18^ Independent monitors validated the clinical data.

### Statistical Analysis

**Figure 1.**
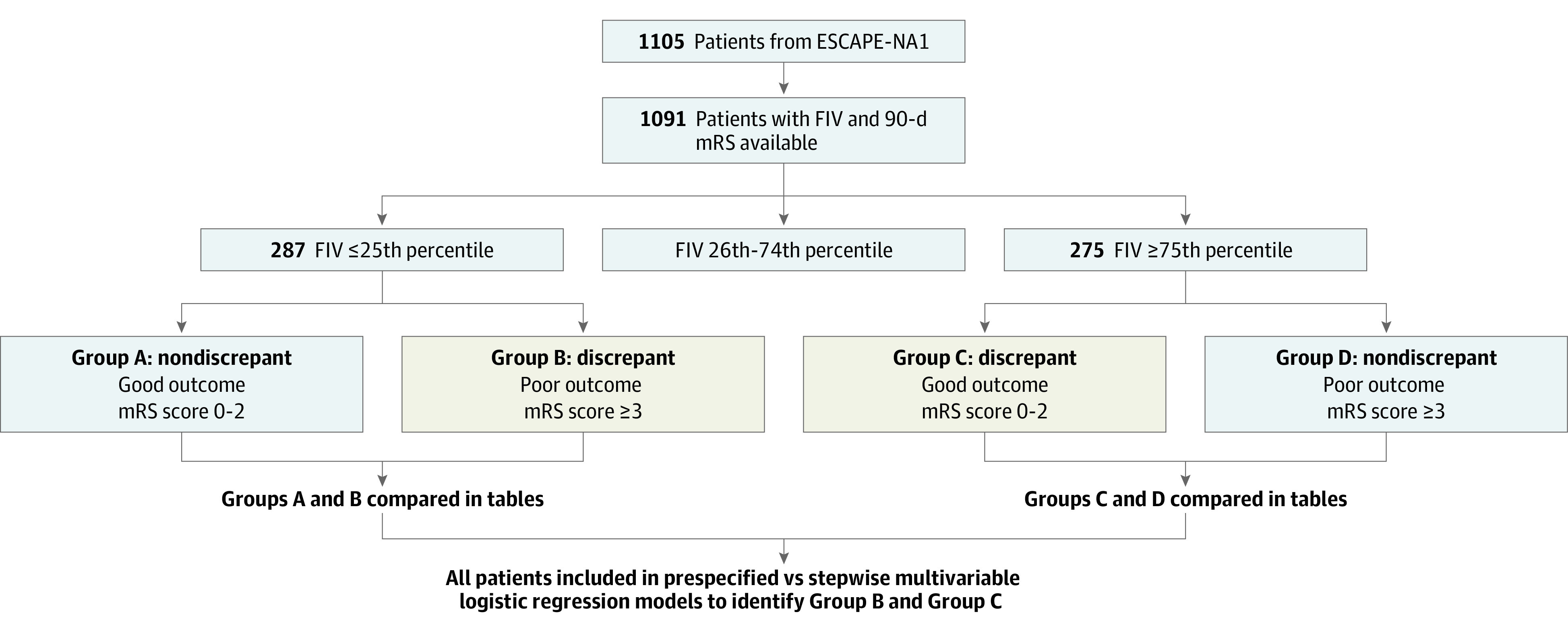
Summary of the Principal Analyses Undertaken in the Study ESCAPE-NA1 indicates Safety and Efficacy of Nerinetide in Subjects Undergoing Endovascular Thrombectomy for Stroke; FIV, follow-up infarct volume; mRS, modified Rankin Scale. Group names (A-D) used in this figure purely for illustrative purposes.

Our analytical process is summarized in Figure 1. Patients with FIV in the 25th percentile or lower and with FIV in the 75th percentile or greater were identified (percentiles rounded to nearest milliliter). Discrepant cases were defined as patients with 90-day mRS score of 3 or greater despite small FIV (ie, ≤25th percentile) or mRS score of 2 or less despite large FIV (ie, ≥75th percentile). These cases were compared with nondiscrepant cases according to FIV size. We examined the imaging modality for FIV ascertainment and other 90-day outcomes to confirm prior findings that discrepancies did not relate to such factors (pseudodiscrepancies).^16^ Based on the prior exploratory findings from the ESCAPE trial, we hypothesized that comorbidities, such as vascular risk factors and cancer, would occur more frequently among patients with small FIV and higher mRS score (vs others with small FIV) and less frequently among patients with large FIV and higher mRS score in the ESCAPE-NA1 trial. We hypothesized that poststroke SAEs would occur more frequently among patients with small FIV and higher mRS score and less frequently among patients with large FIV and lower mRS score. We used Fisher exact tests for proportions and Wilcoxon rank-sum tests for continuous and pseudocontinuous variables.

We used multivariable logistic regression models to validate the performance of variables identified in the preceding ESCAPE trial as associated with discrepant cases among all patients. Analyses were adjusted for FIV (as a continuous variable), receipt of nerinetide and/or alteplase, and the interaction between alteplase and nerinetide, which was observed in the ESCAPE-NA1 trial.^17^ Separate models were used to test the association between discrepant cases and pretreatment variables, with prespecified variables of age, cancer (for small FIV and higher mRS score), and vascular risk factors as well as treatment-related and posttreatment variables (chiefly SAEs). To build on prior work, we sought to better delineate the SAEs most associated with these discrepant outcomes. Since the 24-hour/48-hour NIHSS both reflects a lesion’s functional eloquence and is itself an outcome measure, we excluded the NIHSS from our primary analysis; however, we also examined model performance when the NIHSS was included. We compared the performance of our prespecified models with models generated by stepwise logistic regression of pretreatment and treatment-related/posttreatment variables that were significant on univariate analyses. The purpose of the stepwise models was to provide a data-derived comparison for the performance of the prespecified models. In deciding on the number of factors that could be included in our multivariable models, we used the simple criterion of having at least 10 events per variable included in the logistic regression.^22,23^ As there were 42 patients with small FIV and higher mRS score and 65 patients with large FIV and lower mRS score, we included a maximum of 4 variables in our multivariable models for the association with small FIV and higher mRS score and a maximum of 6 variables for association with large FIV and lower mRS score. We used k-fold cross-validation to generate the area under the receiver operating characteristic curve (AUC) for each model, randomly splitting the data set into 5 equally sized groups.^24,25^ As model calibration diagnostics, we used Pearson χ^2^ and Hosmer-Lemeshow tests.^26^

In sensitivity analyses for our dichotomous outcomes, we examined model performance when defining small infarcts as FIV in the 10th percentile or lower and large infarcts as FIV in the 90th percentile or greater, and when defining favorable outcome as mRS score of 3 or less. Additional analyses restricted the sample to those with FIV in the 25th percentile or lower and with FIV in the 75th percentile or greater, assessing variables associated with poor and good outcomes, respectively, in these restricted samples. Statistical significance was set at 2-sided *P* < .05. In addition, the Hochberg method was used to report correction for multiple comparisons (*P* < .0013).^27^ Analyses were performed using Stata MP version 16.1 (StataCorp).

## Results

Among 1105 patients, FIV and 90-day mRS were both available for 1091 (98.7%). The median (IQR) age was 70.8 (60.8-79.8) years, 549 patients (49.7%) were women, the median (IQR) prestroke mRS score was 0 (0-0), and the median (IQR) NIHSS was 17 (12-21). Other characteristics appear in eTable 1 in Supplement 1. FIV was determined using MRI for 445 patients (40.8%); the remainder underwent CT. Median FIV was 24.9 mL (25th percentile, 6.6 mL; 75th percentile, 92.2 mL; 10th percentile, 1.2 mL; 90th percentile, 214.8 mL). Of 287 patients with FIV of 7 mL or less (≤25th percentile), 42 (14.6%) had mRS scores of 3 or greater; 65 of 275 patients (23.6%) with FIV of 92 mL or greater (≥75th percentile) had mRS scores of 2 or less. These patients were considered discrepant cases (9.8% of cohort) for primary analyses (eFigure 1 in Supplement 1). The number of patients with FIV-mRS discrepancies by other definitions of FIV and mRS are described in eTable 2 in Supplement 1.

### Small FIV and Higher mRS Score

Compared with patients with FIV of 7 mL or less and an mRS score of 2 or less, patients with FIV of 7 mL or less and mRS score of 3 or greater (ie, discrepant cases) less often had MRI for FIV determination than CT (7 [17.1%] vs 87 [35.5%]; *P* = .02). This difference remained on examining those with FIV of 1 mL or less (1 [4.4%] vs 93 [35.4%]; *P* = .002) but was not significant with the Hochberg correction. Discrepant cases also fared poorly on other outcomes, including the BI, hospital length of stay, and residence at follow-up (eTable 3 in Supplement 1).

Examining pretreatment variables (eTable 4 in Supplement 1), patients with discrepant outcomes were older (eFigure 2A in Supplement 1) and less likely to have prestroke mRS score of 0 (25 [61.0%] vs 206 [84.1%]; *P* = .0010). Overall, 22 patients had improvement in mRS scores from baseline to 90 days, but none of them had FIV of 7 mL or less with an mRS score of 2 or less. Discrepant cases had similar NIHSS and deficits at baseline as nondiscrepant cases, and did not differ in infarct location (per Alberta Stroke Program Early CT Score [ASPECTS] regions or when separating into deep [thalamic/basal ganglia], superficial/cortical, or mixed location based on the baseline ASPECTS scan) or left vs right hemisphere involvement.

**Figure 2.**
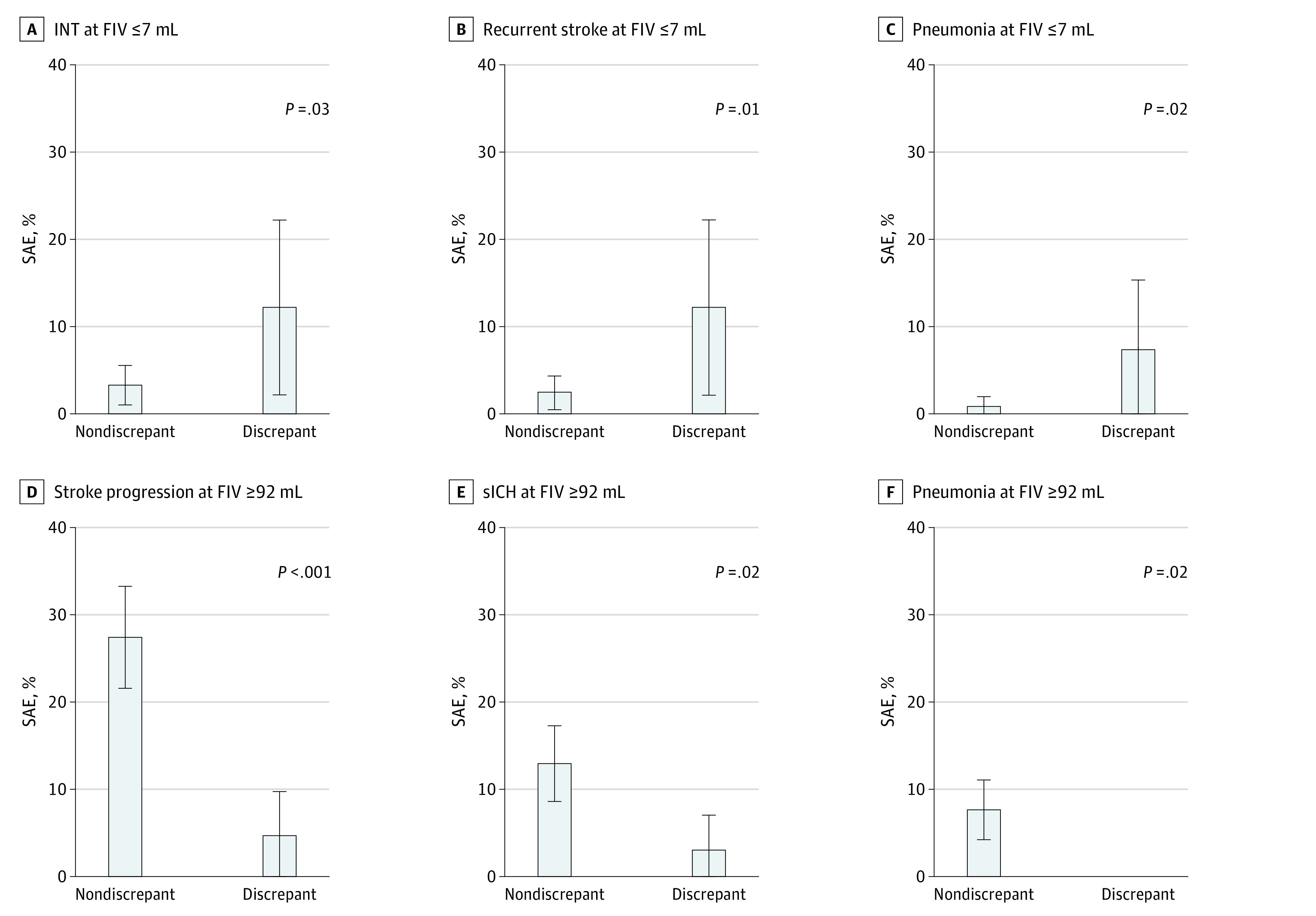
Key Serious Adverse Events (SAEs) That Differed Between Patients With Discrepant vs Nondiscrepant Outcomes, With Small and Large Follow-up Infarct Volume (FIV) SAEs occurred more frequently among patients with poor functional outcome despite small FIV compared with those with small FIV and good outcome. SAEs occurred less frequently among patients with good functional outcome despite large FIV compared with those with large FIV and poor outcome. However, only stroke progression was significant after applying the Hochberg correction for multiple comparisons. Whiskers represent 95% CIs. *P* values shown are from Fisher exact tests comparing discrepant and nondiscrepant cases. INT indicates infarct in new territory; sICH, symptomatic intracerebral hemorrhage.

Examining treatment-related and posttreatment variables (eTable 5 in Supplement 1), discrepant cases had lower hemoglobin and hematocrit at 24 hours after stroke (eg, mean 24-hour hemoglobin: 11.10 g/dL [95% CI, 10.53-11.67 g/dL] vs 12.20 g/dL [95% CI, 11.99-12.42 g/dL] [to convert to grams per liter, multiply by 10]; *P* = .0004), and higher 24-hour/48-hour NIHSS (eg, mean 24-hour NIHSS: 6.5 [95% CI, 4.7-8.2] vs 2.5 [95% CI, 2.1-3.0]; *P* < .0001). SAEs were more common in discrepant cases (20 [48.9%] vs 53 [21.6%]; *P* = .001) (Figure 2A-C). On examining the different types of SAEs, INTs were numerically more common in discrepant cases (5 [12.2%] vs 8 [3.3%]; *P* = .03), as were recurrent strokes (5 [12.2%] vs 6 [2.5%]; *P* = .01) and systemic SAEs (13 [31.7%] vs 17 [6.9%]; *P* < .001), especially pneumonia (3 [7.3%] vs 2 [0.8%]; *P* = .02) and congestive heart failure (CHF; 3 [7.3%] vs 2 [0.8%]; *P* = .02).

In prespecified multivariable logistic regression, pretreatment factors of older age, comorbid cancer, and vascular risk factors were independently associated with mRS score of 3 or greater despite FIV of 7 mL or less (Table 1). Age and vascular risk factors were also selected by the stepwise model, which gave a similar goodness-of-fit and cross-validated AUC. There was no difference between the AUCs of the prespecified and stepwise models (0.92 [95% CI, 0.89-0.95] vs 0.93 [95% CI, 0.90-0.95]; *P* = .42). Prestroke mRS was not independently associated with small PIV and poor outcome once these other factors were considered, and adjustment for infarct location (deep/cortical/mixed) did not alter results. The prespecified posttreatment factor of any SAE was associated with small FIV and poor outcome (Table 1), giving an AUC of 0.92 (95% CI, 0.90-0.95), which was similar to that of the stepwise model (AUC, 0.94 [95% CI, 0.91-0.96]; *P* = .14). The prespecified model had a similar AUC (0.91 [95% CI, 0.88-0.94]) after replacing the variable with more specific SAEs (ie, INT, recurrent stroke, pneumonia, CHF) noted on univariable analyses (eTable 6 in Supplement 1). INT and recurrent stroke were also key posttreatment factors selected by the stepwise model, in addition to hemoglobin at 24 hours and any systemic SAEs (including pneumonia and CHF), giving a similar AUC. With poor outcome defined as mRS of 4 or greater, the stepwise models selected similar pretreatment and posttreatment variables as the prespecified models, with similar performance (eTable 7 in Supplement 1). Similar results were seen when small FIV was defined as FIV of 1 mL or less. When 24-hour and 48-hour NIHSS was included as a potential posttreatment variable, the AUCs obtained with both prespecified and stepwise models improved to 0.95 (95% CI, 0.92-0.98), but INT, recurrent stroke, systemic SAEs, and 24-hour hemoglobin remained important (eTable 8 in Supplement 1). Prespecified and stepwise models performed well on limiting the sample to those with FIV of 7 mL or less and performed similarly on limiting the sample to those patients who had an MRI for FIV determination (eTable 9 in Supplement 1).

**Table 1.** Comparison of Prespecified and Stepwise Multivariable Logistic Regression Models for the Association of Pretreatment Variables and Treatment-Related or Posttreatment Variables With the Outcome of mRS Score of 3 or Greater Despite Small FIV Among 1091 Patients From the Safety and Efficacy of Nerinetide in Subjects Undergoing Endovascular Thrombectomy for Stroke Trial

Variable	Outcome: FIV ≤7 mL with mRS score ≥3			
	Model with prespecified variables based on ESCAPE analysis^a^		Model with variables selected by stepwise multivariable logistic regression^a^	
	aOR (95% CI)	*P* value	aOR (95% CI)	*P* value
**Pretreatment factors**				
Age, per year	1.08 (1.04-1.13)	<.001	1.08 (1.04-1.12)	<.001
Cancer	61.65 (2.91-1304.27)	.008	NA	NA
Vascular risk factors, No.	1.30 (1.06-1.59)	.01	1.27 (1.04-1.56)	.02
AUC (95% CI)^b^	0.92 (0.89-0.95)	<.0001	0.93 (0.90-0.95)	<.0001
Pearson χ^2^	344	>.99	329	>.99
Hosmer-Lemeshow χ^2^	0.37	.95	0.46	.93
**Treatment-related and posttreatment factors**				
Any SAE	3.46 (1.72-6.96)	.001	NA	NA
INT	NA	NA	5.39 (1.40-20.79)	.01
Recurrent stroke	NA	NA	12.44 (2.85-54.41)	.001
Systemic SAEs, potentially related to stroke unit care	NA	NA	5.38 (2.15-13.44)	<.001
Hemoglobin level at 24 h, per g/L increase	NA	NA	0.97 (0.95-0.99)	.003
AUC (95% CI)^c^	0.92 (0.90-0.95)	<.0001	0.94 (0.91-0.96)	<.0001
Pearson χ^2^	231	>.99	286	>.99
Hosmer-Lemeshow χ^2^	0.62	.89	0.28	.96

Abbreviations: aOR, adjusted odds ratio; AUC, area under the receiver operating characteristic curve; ESCAPE, Endovascular Treatment for Small Core and Anterior Circulation Proximal Occlusion with Emphasis on Minimizing CT to Recanalization Times; FIV, follow-up infarct volume; INT, infarct in new territory; mRS, modified Rankin Scale; NA, not applicable; SAE, serious adverse event.

^a^ All models were also adjusted for FIV, alteplase, nerinetide, and interaction of alteplase with nerinetide treatment.

^b^ No difference in AUC between pretreatment factor models 1 and 2 (*P* = .42).

^c^ No difference in AUC between posttreatment factor models 1 and 2 (*P* = .14).

### Large FIV and Good Outcome

Compared with patients with FIV of 92 mL or greater and an mRS score of 3 or greater, patients with FIV of 92 mL or greater and an mRS score of 2 or less (ie, discrepant cases) did not differ significantly in MRI use for FIV determination (21 of 65 [32.3%] vs 56 of 216 [25.9%]; *P* = .34). They fared better than nondiscrepant cases on other outcomes, such as BI and residence at follow-up (eTable 3 in Supplement 1).

**Figure 3.**
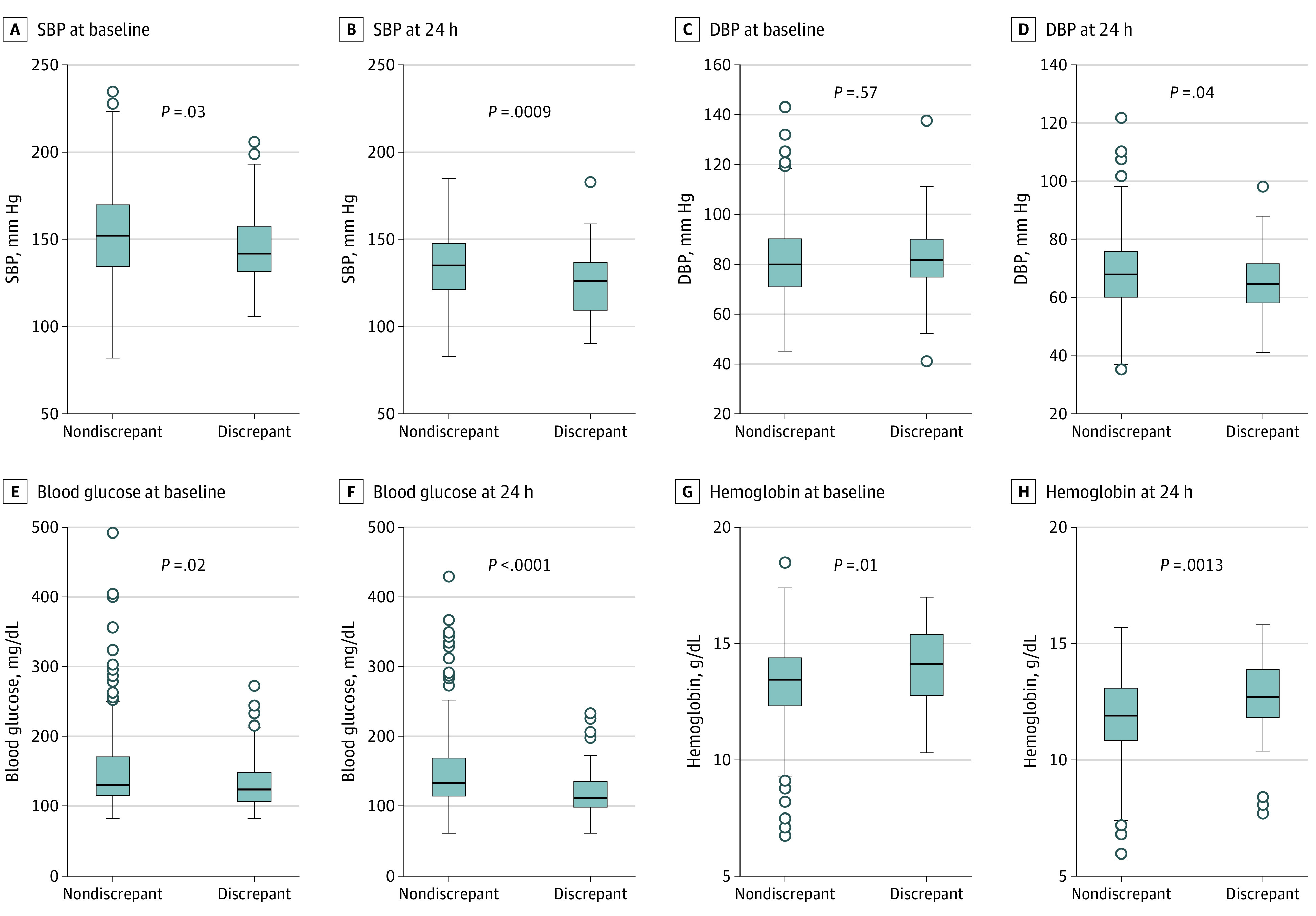
Key Physiological and Laboratory Parameters at Baseline and 24 Hours That Differed Between Patients With Large Follow-up Infarct Volume and Discrepant Modified Rankin Scale Scores vs Nondiscrepant Modified Rankin Scale Scores Discrepant cases, who had good outcome (ie, modified Rankin Scale score, ≤2) despite large follow-up infarct volume (ie, ≥92 mL), had significantly lower systolic blood pressure (SBP) and glucose levels and higher hemoglobin levels at 24 hours vs patients who had large FIV and poor outcome (ie, modified Rankin Scale score ≥3), even after applying the Hochberg correction (significance threshold of .0013). *P* values are from the Wilcoxon rank-sum test comparing discrepant with nondiscrepant cases. DBP indicates diastolic blood pressure. To convert glucose to millimoles per liter, multiply by 0.0555; hemoglobin to grams per liter, multiply by 10.

Examining pretreatment variables (eTable 4 in Supplement 1), patients with discrepant outcomes were younger (eFigure 2B in Supplement 1), were less likely to have diabetes, had lower systolic blood pressure (SBP) and glucose at baseline, and had higher hemoglobin (median [IQR] SBP: 141.5 [131.5-157.5] mm Hg vs 152.0 [134.0-170.0] mm Hg; median [IQR] glucose: 124.1 [106.2-149.5] mg/dL vs 130.1 [115.3-171.2] mg/dL [to convert to millimoles per liter, multiply by 0.0555]; median [IQR] hemoglobin: 14.1 [12.8-15.4] g/dL vs 13.4 [12.3-14.4] g/dL) (Figure 3). They did not differ in baseline NIHSS, ASPECTS scores, or affected regions.

Examining treatment-related and posttreatment variables (eTable 5 in Supplement 1), discrepant cases had lower 24-hour and 48-hour NIHSS (eg, mean 24-hour NIHSS: 9.2 [95% CI, 7.5-10.9] vs 18.4 [95% CI, 17.5-19.4]; *P* < .0001). They had lower SBP and glucose at 24 hours, and higher hemoglobin (eTable 5 in Supplement 1). They less frequently had SAEs (13 [20.3%] vs 139 [64.7%]; *P* < .001) (Figure 2D-F), including likely index stroke-related SAEs, particularly stroke progression; potentially treatment-related SAEs, particularly symptomatic intracerebral hemorrhage (sICH); and systemic SAEs, especially pneumonia (eTable 5 in Supplement 1). Stroke progression was coded if the patient had evident neurological deterioration with imaging correlate (like evolution of prior changes or edema) without better explanation like recurrent stroke or hemorrhage.

In prespecified multivariable logistic regressions, younger age was independently associated with an mRS score of 2 or less despite FIV of 92 mL or greater, although the number of vascular risk factors was not (Table 2). Younger age and absence of diabetes were the pretreatment factors selected by the stepwise model, in addition to higher hemoglobin at baseline, giving a similar goodness of fit and AUC (AUC, 0.76 [95% CI, 0.70-0.82] vs 0.77 [95% CI, 0.71-0.83]; *P* = .82). The prespecified posttreatment factor of any SAE was associated with large FIV and good outcome (Table 2), giving an AUC of 0.80 (95% CI, 0.74-0.87), similar to that obtained by the stepwise model (0.79 [95% CI, 0.72-0.86]; *P* = .92). A higher AUC of 0.87 (95% CI, 0.83-0.92) was achieved in the prespecified model after replacing the SAE variable with the specific SAEs of stroke progression, sICH, and pneumonia noted on univariable analyses (eTable 10 in Supplement 1). Stroke progression and sICH were also selected by the stepwise model, in addition to hemoglobin level (adjusted odds ratio [aOR], 1.02; 95% CI, 1.01-1.04), glucose level (aOR, 0.82; 95% CI, 0.69-0.96), and DBP (aOR, 0.97; 95% CI, 0.94-0.99) at 24 hours, giving a slightly smaller AUC of 0.83 (95% CI, 0.77-0.89; *P* = .046). When good outcome was defined as mRS of 3 or less, the stepwise models selected age and diabetes as pretreatment variables and again selected stroke progression, sICH, and BP (SBP) at 24 hours as posttreatment variables, along with pneumonia (eTable 11 in Supplement 1). These models performed similarly to prespecified models. Similar results were seen when defining large FIV as FIV of 215 mL or greater with different mRS definitions and on limiting the sample to those with FIV of 92 mL or greater. When 24-hour and 48-hour NIHSS was included as a posttreatment variable, the AUCs obtained were similar, and stroke progression, sICH, pneumonia, 24-hour SBP, and hemoglobin level remained relevant (eTable 12 in Supplement 1). All models had satisfactory goodness-of-fit metrics.

**Table 2.** Comparison of Prespecified and Stepwise Multivariable Logistic Regression Models for the Association of Pretreatment Variables and Treatment-Related or Posttreatment Variables With the Outcome of mRS Score of 2 or Less Despite Large FIV Among 1091 Patients in the Safety and Efficacy of Nerinetide in Subjects Undergoing Endovascular Thrombectomy for Stroke Trial

Variable	Outcome: FIV ≥92 mL with mRS score ≤2			
	Model with pre-specified variables based on ESCAPE analysis^a^		Model with variables selected by stepwise multivariable logistic regression^a^	
	aOR(95%CI)	*P *value	aOR(95%CI)	*P* value
Pretreatment factors				
Age, per year	0.97 (0.95-0.99)	.006	0.98 (0.96-0.99)	.02
Vascular risk factors, No.	0.99 (0.84-1.16)	.89	NA	NA
Diabetes	NA	NA	0.38 (0.16-0.92)	.03
Baseline hemoglobin level, per g/L increase	NA	NA	1.02 (1.00-1.03)	.04
AUC (95% CI)^b^	0.76 (0.70-0.82)	<.0001	0.77 (0.71-0.83)	<.0001
Pearson χ^2^	703	>.99	686	>.99
Hosmer-Lemeshow χ^2^	2.49	.47	3.44	.33
**Treatment-related and posttreatment factors**				
Any SAE	0.13 (0.05-0.30)	<.001	NA	NA
Stroke progression	NA	NA	0.05 (0.01-0.25)	<.001
sICH	NA	NA	0.15 (0.03-0.84)	.03
DBP at 24 h, per mm Hg increase	NA	NA	0.97 (0.94-0.99)	.003
Glucose level at 24 h, per mmol/L increase	NA	NA	0.82 (0.69-0.96)	.02
Hemoglobin level at 24 h per g/L increase	NA	NA	1.02 (1.01-1.04)	.009
AUC (95% CI)^c^	0.80 (0.74-0.87)	<.0001	0.79 (0.72-0.86)	<.0001
Pearson χ^2^	830	.34	725	.93
Hosmer-Lemeshow χ^2^	3.11	.38	3.17	.37

Abbreviations: aOR, adjusted odds ratio; AUC, area under the receiver operating characteristic curve; DBP, diastolic blood pressure; ESCAPE, Endovascular Treatment for Small Core and Anterior Circulation Proximal Occlusion with Emphasis on Minimizing CT to Recanalization Times; FIV, follow-up infarct volume; mRS, modified Rankin Scale; NA, not applicable; SAE, serious adverse event; sICH, symptomatic intracerebral hemorrhage.

^a^ All models were also adjusted for FIV, alteplase, nerinetide, and interaction of alteplase with nerinetide treatment.

^b^ No difference in AUC between pretreatment factor models 1 and 2 (*P* = .82).

^c^ No difference in AUC between posttreatment factor models 1 and 2 (*P* = .92).

## Discussion

In this post hoc analysis of the ESCAPE-NA1 trial, discrepancies between FIV and 90-day functional outcome were common, occurring in between 9.8% and 13.6% of patients, depending on the definitions used. We compared the performance of prespecified models, based on hypotheses generated from the ESCAPE trial, with data-derived models selected by stepwise regression. Our results validated that these discrepancies occurred in approximately 1 in 8 patients and are generally not pseudodiscrepancies, with the mRS aligning with other functional outcomes (construct and concurrent validity). Roughly 1 in 5 patients achieved an mRS of 2 or less despite large FIV, emphasizing that poor outcomes are not inevitable with large FIVs. Our analyses highlight the finding that discrepancies between FIV and outcome are associated with a combination of pretreatment, treatment-related, and posttreatment factors, underscoring the complex chain leading to 90-day outcomes,^28^ incompletely captured by FIV.^2,9^

Our analyses of pretreatment factors validate the importance of age and comorbidities, particularly cancer and vascular risk factors, in poor outcomes among patients despite small FIV (predictive validity). Such factors associated with recovery and disability^13^ are not captured by FIV but can help adjust our treatment expectations. Whereas vascular risk factors as a whole were not associated with large FIV and good outcome, diabetes was associated with this discrepant outcome in stepwise analyses.

Pretroke mRS score was not independently associated with FIV-mRS discrepancies in our analyses, but there were few patients with premorbid disability in the ESCAPE-NA1 trial. Again, we saw no difference in right vs left hemisphere involvement, in keeping with prior analyses of trials that concluded that stroke lateralization is not independently associated with global 90-day outcomes scored on the mRS.^29,30^ In ESCAPE, sparing of the lentiform nucleus was associated with an mRS score of 2 or less despite FIV in the 75th percentile or greater, but this was not the case when using different definitions of large FIV, suggesting an unstable association.^16^ Indeed, we found no such association in this study. In keeping with our prior findings, we found that patients with small FIV and higher mRS scores had higher 24-hour and 48-hour NIHSS, while those with large FIV and lower mRS scores had lower NIHSS than nondiscrepant cases, potentially reflecting differences in functional eloquence, but also confirming prior observations of 24-hour NIHSS reflecting 90-day mRS better than FIV.^2,3^

Besides validating the importance of SAEs as a posttreatment factor for FIV-mRS discrepancies, our analyses further clarify the most important SAEs in this regard. For small FIV with higher mRS score, these include INT, recurrent stroke, and SAEs attributable to the quality of stroke unit care (specifically pneumonia and CHF); for large FIV and lower mRS score, they include avoiding stroke progression, sICH, and pneumonia. These findings underscore the importance of high-quality postacute care^31^ with optimization of secondary prevention and mitigation of complications such as pneumonia.^15,32^ Importantly, these complications may also be associated with periprocedural care factors and certain baseline factors; for example, CHF post-EVT may result from excess intravenous fluids through unchecked catheter flush lines during EVT, particularly in a patient with preexisting poor ejection fraction. INTs may be mitigated by neuroprotective strategies and pretreatment with alteplase, as noted in a post hoc analysis of ESCAPE.^14^ Whereas ESCAPE-NA1 was not powered to examine the added value of thrombolysis, future analyses of INT and discrepant outcomes in recent bridging studies may further inform this issue.^33,34,35^ Symptomatic ICH is associated with various nonmodifiable and modifiable factors,^36,37^ including hypertension and hyperglycemia^38,39^; our results support the intuitive result that avoiding sICH will help optimize outcomes even in those with large infarcts.

We identified several additional posttreatment factors, especially in the first 24 hours, that may mediate FIV-mRS discrepancies. These include BP control, glucose, and hemoglobin at 24 hours. Optimal BP parameters after EVT are uncertain; our findings showed that lower BP was favorably associated with functional outcome even among patients with large infarcts.^40,41^ Whether lowering BP will translate to better outcomes remains unclear. Hyperglycemia occurs in approximately 40% of patients with acute ischemic stroke and is associated with worse outcomes.^42,43,44^ Although this has been attributed to greater infarct growth,^43,45^ other mechanisms may also be important, such as hemorrhagic conversion or endothelial dysfunction.^46,47^ Aggressive glycemic control in the first 72 hours after stroke was not shown to be beneficial in the Stroke Hyperglycemia Insulin Network Effort (SHINE) trial, but the population of patients was different, with only 13% receiving EVT.^48^ Concordant with lower hemoglobin at 24 hours being associated with small FIV and higher mRS scores (and higher hemoglobin with large FIV and lower mRS score), anemia has been linked to higher mortality and worse outcome in ischemic stroke.^49,50^ However, optimal hemoglobin concentrations or transfusion thresholds are unclear.^51,52^ It is possible that these factors remain targets for treatment trials.

### Strengths and Limitations

Our study has several strengths, including the use of high-quality data from a randomized clinical trial of EVT recipients, a sample 3 times larger than the ESCAPE trial, relative completeness of data for most factors, and replication of analyses using different FIV and mRS definitions. However, there are some important limitations. ESCAPE-NA1 was not powered to detect differences between discrepant and nondiscrepant cases. Whereas we used simple events-per-variable criteria for our multivariable models, more formal power calculation methods have been developed by others.^53^ FIV was measured using MRI or CT at 24 to 48 hours; measuring infarct volumes later (eg, at 1 week or 6 months) on MRI may offer varying estimates.^54^ Although we had minimum requirements for the follow-up scans at each study site, minor scanner or protocol differences, which were simply impractical to fully homogenize in this large international trial, may have affected the calculation of FIV. Whereas patients with small FIV and higher mRS scores were more likely to have CT for FIV determination than those with small FIV and lower mRS scores, suggesting that FIVs of some discrepant patients may have been underestimated due to the lower sensitivity of CT, our results were similar on restricting the sample to those who had MRI. Whereas cancer was associated with poor outcome despite small FIV, this requires cautious interpretation, as there were few patients with cancer in the trial. Our analysis of stroke lesion location was limited by use of ASPECTS-based location classifiers.^55^ Future analyses could include more detailed and precise classifications to examine how the eloquence of the affected territory (eg, internal capsule with a small infarct) or spared territory (eg, unaffected motor cortex with a large infarct) may explain discrepancies between FIV and mRS scores. We did not assess prestroke vascular burden, such as leukoaraiosis, atrophy, or old infarcts, which are potentially relevant for poststroke outcomes.^56^ Our classification of SAEs (eg, potentially related to stroke unit care) involved subjective judgements; others may classify them differently. However, this approach helped us understand the differential contribution of these SAEs to observed discrepancies. While this was not a focus of our analysis, some relevant pretreatment and posttreatment factors may have varied among the 48 centers in the trial, potentially affecting the occurrence of FIV-mRS discrepancies. We used stepwise models to provide a data-derived comparison for our prespecified models. While these models are simple and commonly used, they have important limitations, and more sophisticated methods may be considered.^57^ We used various dichotomous definitions of small and large FIV and good and poor mRS scores for our analyses; future analyses may seek to use sliding dichotomies or ordinal approaches to classify FIV-mRS discrepancies to make use of the full range of infarct volumes and mRS scores.^58^

## Conclusions

In this study, discrepancies between functional ability and infarct volume were associated with differences in pretreatment factors, such as age; comorbidities, such as cancer or vascular risk factors; and poststroke complications related to the evolution of the index stroke, secondary prevention, and quality of periprocedural and stroke unit care. Besides prevention of such complications, optimization of BP, glucose levels, and potentially hemoglobin levels constitute important modifiable treatment-related posttreatment factors for further study.

## Notes

Supplement 1. **eTable 1.** Baseline Characteristics of Participants in the Trial Who Had Both FIV and 90-Day mRS Data **eTable 2.** Patients With Discrepancies Between FIV and Functional Outcomes per the Modified Rankin Scale (mRS) at 90 Days, Using Different Definitions of Small and Large FIV and Favorable and Unfavorable Outcome **eTable 3.** Univariate Comparison of Imaging Modality and 90-Day Outcomes in Discrepant vs Nondiscrepant Cases **eTable 4.** Univariate Comparison of Pretreatment Factors Discrepant vs Nondiscrepant Cases **eTable 5.** Comparison of Treatment-Related and Posttreatment Factors, Including Serious Adverse Events (SAEs), in Discrepant vs Nondiscrepant Cases **eTable 6.** Comparison of Prespecified and Stepwise Multivariable Logistic Regression Models for the Association of Pretreatment Variables Among 1091 Patients and Treatment-Related or Posttreatment Variables Among 1086 Patients in Prespecified Model and 631 Patients in Stepwise Model for the Outcome of mRS Score of 3 or Greater Despite FIV of 7 mL or Less in the ESCAPE-NA1 Trial **eTable 7.** Comparison of Prespecified and Stepwise Multivariable Logistic Regression Models for the Association of Pretreatment Variables Among 1094 Patients and Treatment-Related or Posttreatment Variables Among 1089 Patients for the Outcome of mRS Score of 4 or Greater Despite FIV of 7 mL or Less in the ESCAPE-NA1 Trial **eTable 8.** Comparison of Prespecified and Stepwise Multivariable Logistic Regression Models for the Association of Treatment-Related or Posttreatment Variables Among 918 Patients for the Outcome of mRS Score of 3 or Greater Despite FIV of 7 mL or Less in the ESCAPE-NA1 Trial With 24-Hour or 48-hour NIHSS Included as a Posttreatment Variable **eTable 9.** Comparison of Prespecified and Stepwise Multivariable Logistic Regression Models for the Association of Pretreatment Variables Among 254 Patients and Treatment-Related or Posttreatment Variables Among 256 Patients for Outcome of mRS Score of 4 or Greater Despite FIV of 7 mL or Less in the ESCAPE-NA1 Trial, Restricted to Patients With MRI for FIV Determination **eTable 10.** Comparison of Prespecified and Stepwise Multivariable Logistic Regression Models for the Association of Pretreatment Variables Among 1091 Patients and Treatment-Related or Posttreatment Variables Among 1082 Patients for the Outcome of mRS Score of 2 or Less Despite FIV of 92 mL or Greater in the ESCAPE-NA1 Trial **eTable 11.** Comparison of Prespecified and Stepwise Multivariable Logistic Regression Models for the Association of Pretreatment Variables Among 1091 Patients and Treatment-Related or Posttreatment Variables Among 1082 Patients for the Outcome of mRS Score of 3 or Less Despite FIV of 92 mL or Greater in the ESCAPE-NA1 Trial **eTable 12.** Comparison of Prespecified and Stepwise Multivariable Logistic Regression Models for the Association Treatment-Related or Posttreatment Variables Among 924 Patients for the Outcome of mRS Score of 2 or Less Despite FIV of 92 mL or Greater in the ESCAPE-NA1 Trial With 24-Hour or 48-hour NIHSS Included as a Posttreatment Variable **eFigure 1.** Proportions of Patients in the ESCAPE-NA1 Trial With Discrepancies Between FIV and 90-day mRS Score, Based on the Definitions Used in this Study **eFigure 2.** Proportion of Patients in Each Age Group With 90-Day mRS Score of 3 or Greater Despite Small FIV and mRS Score of 2 or Less Despite Large FIV

Supplement 2.
Nonauthor Collaborators
