# Supplementary material for: Assessment of Discrepancies Between Follow-up Infarct Volume and 90-Day Outcomes Among Patients With Ischemic Stroke Who Received Endovascular Therapy
Source: JAMA Netw Open. 2021 Nov 5;4(11):e2132376. doi: 10.1001/jamanetworkopen.2021.32376 (PMC8571657; doi:10.1001/jamanetworkopen.2021.32376)
Supplement: Supplement 1. — eTable 1. Baseline Characteristics of Participants in the Trial Who Had Both FIV and 90-Day mRS Data eTable 2. Patients With Discrepancies Between FIV and Functional Outcomes per the Modified Rankin Scale (mRS) at 90 Days, Using Different Definitions of Small and Large FIV and Favorable and Unfavorable Outcome eTable 3. Univariate Comparison of Imaging Modality and 90-Day Outcomes in Discrepant vs Nondiscrepant Cases eTable 4. Univariate Comparison of Pretreatment Factors Discrepant vs Nondiscrepant Cases eTable 5. Comparison of Treatment-Related and Posttreatment Factors, Including Serious Adverse Events (SAEs), in Discrepant vs Nondiscrepant Cases eTable 6. Comparison of Prespecified and Stepwise Multivariable Logistic Regression Models for the Association of Pretreatment Variables Among 1091 Patients and Treatment-Related or Posttreatment Variables Among 1086 Patients in Prespecified Model and 631 Patients in Stepwise Model for the Outcome of mRS Score of 3 or Greater Despite FIV of 7 mL or Less in the ESCAPE-NA1 Trial eTable 7. Comparison of Prespecified and Stepwise Multivariable Logistic Regression Models for the Association of Pretreatment Variables Among 1094 Patients and Treatment-Related or Posttreatment Variables Among 1089 Patients for the Outcome of mRS Score of 4 or Greater Despite FIV of 7 mL or Less in the ESCAPE-NA1 Trial eTable 8. Comparison of Prespecified and Stepwise Multivariable Logistic Regression Models for the Association of Treatment-Related or Posttreatment Variables Among 918 Patients for the Outcome of mRS Score of 3 or Greater Despite FIV of 7 mL or Less in the ESCAPE-NA1 Trial With 24-Hour or 48-hour NIHSS Included as a Posttreatment Variable eTable 9. Comparison of Prespecified and Stepwise Multivariable Logistic Regression Models for the Association of Pretreatment Variables Among 254 Patients and Treatment-Related or Posttreatment Variables Among 256 Patients for Outcome of mRS Score of 4 or Greater Despite FIV of 7 mL or Less in t [file jamanetwopen-e2132376-s001.pdf]

Supplemental Online Content

Ganesh A, Ospel JM, Menon BK, et al; ESCAPE-NA1 Trial Investigators. Assessment of discrepancies between follow-up infarct volume and 90-day outcomes among patients with ischemic stroke who received endovascular therapy. *JAMA Netw Open.* 2021;4(11):e2132376. doi:10.1001/jamanetworkopen.2021.32376

eTable 1. Baseline Characteristics of Participants in the Trial Who Had Both FIV and 90-Day mRS Data

eTable 2. Patients With Discrepancies Between FIV and Functional Outcomes per the Modified Rankin Scale (mRS) at 90 Days, Using Different Definitions of Small and Large FIV and Favorable and Unfavorable Outcome

eTable 3. Univariate Comparison of Imaging Modality and 90-Day Outcomes in Discrepant vs Nondiscrepant Cases

eTable 4. Univariate Comparison of Pretreatment Factors Discrepant vs Nondiscrepant Cases

eTable 5. Comparison of Treatment-Related and Posttreatment Factors, Including Serious Adverse Events (SAEs), in Discrepant vs Nondiscrepant Cases

eTable 6. Comparison of Prespecified and Stepwise Multivariable Logistic Regression Models for the Association of Pretreatment Variables Among 1091 Patients and Treatment-Related or Posttreatment Variables Among 1086 Patients in Prespecified Model and 631 Patients in Stepwise Model for the Outcome of mRS Score of 3 or Greater Despite FIV of 7 mL or Less in the ESCAPE-NA1 Trial

eTable 7. Comparison of Prespecified and Stepwise Multivariable Logistic Regression Models for the Association of Pretreatment Variables Among 1094 Patients and Treatment-Related or Posttreatment Variables Among 1089 Patients for the Outcome of mRS Score of 4 or Greater Despite FIV of 7 mL or Less in the ESCAPE-NA1 Trial

eTable 8. Comparison of Prespecified and Stepwise Multivariable Logistic Regression Models for the Association of Treatment-Related or Posttreatment Variables Among 918 Patients for the Outcome of mRS Score of 3 or Greater Despite FIV of 7 mL or Less in the ESCAPE-NA1 Trial With 24-Hour or 48-hour NIHSS Included as a Posttreatment Variable

eTable 9. Comparison of Prespecified and Stepwise Multivariable Logistic Regression Models for the Association of Pretreatment Variables Among 254 Patients and Treatment-Related or Posttreatment Variables Among 256 Patients for Outcome of mRS Score of 4 or Greater Despite FIV of 7 mL or Less in the ESCAPE-NA1 Trial, Restricted to Patients With MRI for FIV Determination

eTable 10. Comparison of Prespecified and Stepwise Multivariable Logistic Regression Models for the Association of Pretreatment Variables Among 1091 Patients and Treatment-Related or Posttreatment Variables Among 1082 Patients for the Outcome of mRS Score of 2 or Less Despite FIV of 92 mL or Greater in the ESCAPE-NA1 Trial

eTable 11. Comparison of Prespecified and Stepwise Multivariable Logistic Regression Models for the Association of Pretreatment Variables Among 1091 Patients and Treatment-Related or

© 2021 Ganesh A et al. *JAMA Network Open.*

Posttreatment Variables Among 1082 Patients for the Outcome of mRS Score of 3 or Less Despite FIV of 92 mL or Greater in the ESCAPE-NA1 Trial

eTable 12. Comparison of Prespecified and Stepwise Multivariable Logistic Regression Models for the Association Treatment-Related or Posttreatment Variables Among 924 Patients for the Outcome of mRS Score of 2 or Less Despite FIV of 92 mL or Greater in the ESCAPE-NA1 Trial With 24-Hour or 48-hour NIHSS Included as a Posttreatment Variable

eFigure 1. Proportions of Patients in the ESCAPE-NA1 Trial With Discrepancies Between FIV and 90-day mRS Score, Based on the Definitions Used in this Study

eFigure 2. Proportion of Patients in Each Age Group With 90-Day mRS Score of 3 or Greater Despite Small FIV and mRS Score of 2 or Less Despite Large FIV

This supplemental material has been provided by the authors to give readers additional information about their work.

© 2021 Ganesh A et al. *JAMA Network Open.*

eTable 1. Baseline Characteristics of Participants in the Trial Who Had Both FIV and 90-Day mRS Data

Age, Median years (IQR)	70.9 (60.7-79.8)
Sex – female, N (%)	549 (49.7%)
Race	
White, N (%)	881 (80.8)
Asian, N (%)	105 (9.6)
**Medical history**	
Hypertension, N (%)	764 (70.0)
Non-smoker (lifelong), N (%)	559 (51.4)
Hyperlipidemia, N (%)	506 (46.4)
Atrial fibrillation, N (%)	385 (35.3)
Ischemic heart disease, N (%)	250 (22.9)
Diabetes, N (%)	215 (19.7)
Congestive heart failure, N (%)	135 (12.4)
Any past stroke, N (%)	155 (14.2)
Peripheral vascular disease, N (%)	58 (5.3)
Chronic renal failure, N (%)	63 (5.8)
Recent major surgery, N (%)	39 (3.6)
**Clinical factors**	
Witnessed stroke onset, N (%)	624 (57.4)
Stroke on awakening, N (%)	171 (15.7)
Right hemisphere stroke, N (%)	575 (52.7)
NIHSS score, median (IQR)	17 (12-21)
Systolic blood pressure (mmHg), median (IQR)	146 (130-165)
Blood glucose (mmol/L), median (IQR)	6.7 (5.9-7.9)
ASPECTS, median (IQR)	8 (7-9)
Occlusion site – ICA, N (%)	211 (19.3)
Collaterals – good, N (%)	699 (64.1)
**Treatment and workflow**	
Nerinetide treatment, N (%)	544 (49.9)
Alteplase treatment, N (%)	653 (59.9)
Interhospital transfer to endovascular center, N (%)	456 (41.8)
General anesthesia use, N (%)	187 (17.2)
Onset to randomization (min), median (IQR)	187 (121-309)
Endovascular center arrival to arterial access or puncture (min), median (IQR)	59 (42-84)
Study drug start to reperfusion (min), median (IQR)	22 (8-41)
eTICI (expanded Thrombolysis in Cerebral Ischemia)	
2b-2c-3, N (%)	947 (87.3)
2c-3, N (%)	504 (46.5)

© 2021 Ganesh A et al. *JAMA Network Open.*

eTable 2. Patients With Discrepancies Between FIV and Functional Outcomes per the Modified Rankin Scale (mRS) at 90 Days, Using Different Definitions of Small and Large FIV and Favorable and Unfavorable Outcome

	**Definition of favourable/unfavourable outcome at 90-days**	
**Definition of small/large FIV**	Favourable: mRS≤2<br><br>Unfavourable: mRS≥3	Favourable: mRS≤3<br><br>Unfavourable: mRS≥4
**SET 1**		
FIV≤7mL (25th percentile) and unfavourable outcome, N(%)	42/287 (14.6)	23/287 (8.0)
FIV≥92mL (75th percentile) and favourable outcome, N(%)	65/275 (23.6)	106/275 (38.6)
**Total "discrepant cases" in the cohort, N(%)**	107/1,091 (9.8)	129/1,091 (11.8)
**SET 2**		
FIV≤1mL (10th percentile) and unfavourable outcome, N(%)	24/103 (23.3)	8/103 (7.8)
FIV≥215mL, (90th percentile) and favourable outcome, N(%)	10/109 (9.2)	18/109 (16.5)
**Total "discrepant cases" in the cohort, N(%)**	34/1,091 (3.1)	26/1,091 (2.4)

Using the most inclusive definitions (FIV≤7mL/mRS≥3 or FIV≥92/mRS≤3), discrepant outcomes occurred in 148/1,091 (13.6%) of the cohort.

© 2021 Ganesh A et al. *JAMA Network Open.*

eTable 3. Univariate Comparison of Imaging Modality and 90-Day Outcomes in Discrepant vs Nondiscrepant Cases

Patients with small follow-up infarct volume or FIV (defined as FIV≤7mL) and poor outcome (defined as 90-day mRS≥3) considered discrepant; patients with small FIV and good outcome (mRS≤2) considered nondiscrepant; patients with large FIV (defined as FIV≥92mL) and good outcome (defined as mRS≤2) considered discrepant; patients with large FIV and bad outcome considered nondiscrepant. Variables significant at p<0.05 are bolded; those also significant with the Hochberg correction (p<0.0013) are indicated with asterisk (*).

	**(A) FIV≤7mL**			**(B) FIV≥92mL**		
**Variable**	**Discrepant**	**Non-discrepant**	**P-value**	**Discrepant**	**Non-discrepant**	**P-value**
MRI vs CT for FIV(%)	7/41 (17.1)	87/245 (35.5)	**0.020**	21/65 (32.3)	56/216 (25.9)	0.34
Barthel Index/BI (mean, 95%CI)	68.6 (58.5-78.7)	99.1 (98.7-99.4)	**<0.0001***	97.2 (95.7-98.6)	45.7 (39.7-51.6)	**<0.0001***
- BI<95 (%)	22/33 (66.7)	7/243 (2.9)	**<0.001***	10/65 (15.4)	111/122 (91.0)	**<0.001***
Discharge location: not home (%)	31/40 (77.5)	82/245 (33.5)	**<0.001***	44/65 (67.7)	210/214 (98.1)	**<0.001***
90-day residence: not home (%)	25/41 (61.0)	13/245 (5.3)	**<0.001***	9/65 (13.9)	170/215 (79.1)	**<0.001***
Hospital LOS (mean days, 95%CI)	23.3 (17.4-29.1)	9.8 (8.2-11.4)	**<0.0001***	26.1 (22.5-29.7)	22.6 (17.5-27.8)	0.91

© 2021 Ganesh A et al. *JAMA Network Open.*

eTable 4. Univariate Comparison of Pretreatment Factors Discrepant vs Nondiscrepant Cases

Patients with small follow-up infarct volume or FIV (defined as FIV≤7mL) and poor outcome (defined as 90-day mRS≥3) considered discrepant; patients with small FIV and good outcome (mRS≤2) considered nondiscrepant; patients with large FIV (defined as FIV≥92mL) and good outcome (defined as mRS≤2) considered discrepant; patients with large FIV and bad outcome considered nondiscrepant. Variables significant at p<0.05 are bolded; those also significant with Hochberg correction (p<0.0013) are indicated with asterisk (*).

	**(A) FIV≤7mL**			**(B) FIV≥92mL**		
**Variable**	**Discrepant**	**Non-discrepant**	**P-value**	**Discrepant**	**Non-discrepant**	**P-value**
**Demographics, Co-morbidities, and Pre-Treatment Laboratory Parameters**						
**Age (Mean, 95%CI)**	80.1 (76.6-83.7)	70.1 (68.4-71.7)	**<0.0001***	65.2 (62.1-68.2)	72.3 (70.6-74.0)	**0.0001***
Female (%)	22/41 (53.7)	133/245 (54.3)	1.00	24/65 (36.9)	108/216 (50.0)	0.067
Race – Non-White (%)	4/31 (9.8)	46/245 (18.8)	0.81	13/65 (20.0)	59/216 (27.3)	0.86
**Cancer (%)**	2/41 (4.9)	0/245	**0.020**	1/65 (1.5)	1/216 (0.5)	0.41
**Coronary artery disease (%)**	16/41 (39.0)	49/245 (20.0)	**0.014**	17/65 (26.2)	53/215 (24.7)	0.87
Congestive heart failure (%)	9/41 (22.0)	35/245 (14.3)	0.24	6/65 (9.2)	23/215 (10.7)	0.82
Stroke or TIA (%)	10/41 (24.4)	37/245 (15.1)	0.17	7/65 (10.8)	31.215 (14.4)	0.54
**Stroke or TIA in last 90-days (%)**	1/41 (2.4)	11/245 (4.5)	1.00	6/65 (9.2)	4/215 (1.9)	**0.012**
Intracerebral hemorrhage(%)	2/41 (4.9)	3/245 (1.2)	0.15	0/65	4/215 (1.9)	0.58
Peripheral vascular disease (%)	4/41 (9.8)	10/245 (4.1)	0.12	5/65 (7.7)	17/215 (7.9)	1.00
Chronic renal failure	5/41 (12.2)	13/245 (5.3)	0.15	5/65 (7.7)	20/215 (9.3)	0.81
Dyslipidemia (%)	26/41 (63.4)	115/245 (46.9)	0.063	23/65 (35.4)	106/215 (49.3)	0.064
**Atrial Fibrillation (%)**	**25/41 (61.0)**	**98/245 (40.0)**	**0.016**	20/65 (30.8)	77/215 (35.8)	0.55
Hypertension (%)	34/41 (82.9)	179/245 (73.1)	0.25	43/65 (66.2)	160/215 (74.4)	0.21
**Diabetes (%)**	9/41 (22.0)	44/245 (18.0)	0.52	**7/65 (10.8)**	**62/215 (28.8)**	**0.003**
Smoking – Current/Ex (%)	18/41 (43.9)	113/245 (46.1)	0.87	37/65 (56.9)	91/212 (42.9)	0.064
Any vascular risk factors (%)	40/41 (97.6)	227/245 (92.7)	0.33	61/65 (93.9)	200/216 (92.6)	1.00
**Number of vascular risk factors (median, IQR)**	**4 (2-5)**	**3 (2-4)**	**0.0043**	2 (1-4)	3 (2-4)	0.18
Head trauma in last 90-days (%)	0/41	3/245 (1.2)	1.00	0/65	3/215 (1.4)	1.00
Major surgery in last 90-days (%)	1/41 (2.4)	11/245 (4.5)	1.00	3/65 (4.6)	8/215 (3.7)	0.72
Endocarditis (%)	1/41 (2.4)	0	0.14	1/65 (1.5)	1/216 (0.5)	0.41
Intracardiac thrombus	0/41	2/245 (0.8)	1.00	0/65	1/216 (0.5)	1.00
Patent Foramen Ovale	0/41	2/245 (0.8)	1.00	0	0	
**Pre-stroke mRS (%)**						
0	25/41 (61.0)	206/245 (84.1)	**0.0010***	53/65 (81.5)	173/213 (81.2)	0.086
1	9/41 (22.0)	28/245 (11.4)		11/65 (16.9)	23/213 (10.8)	
2	7/41 (17.1)	11/245 (4.5)		1/65 (1.5)	17/213 (8.0)	
**SBP** (Mean mmHg, 95%CI)	151.5 (143.7-159.3)	146.4 (143.2-149.6)	0.30	**146.2 (140.7-151.6)**	**153.4 (149.7-157.1)**	**0.031**

© 2021 Ganesh A et al. *JAMA Network Open.*

DBP (Mean mmHg, 95%CI)	84.1 (79.4-88.9)	81.1 (79.1-83.0)	0.20	82.2 (78.2-86.1)	81.9 (79.7-84.1)	0.57
Heart Rate (Mean bpm, 95%CI)	84.0 (76.7-91.3)	83.0 (80.5-85.4)	0.81	84.4 (80.3-88.6)	83.8 (81.2-86.4)	0.55
Temperature (Mean C, 95%CI)	36.3 (36.1-36.6)	36.4 (36.3-36.5)	0.89	36.4 (36.2-36.5)	36.3 (36.2-36.5)	0.77
**Hemoglobin (Mean g/L, 95%CI)**	**127.6 (121.4-133.8)**	**135.2 (133.0-137.3)**	**0.0027**	**140.2 (136.0-144.3)**	**133.1 (130.5-135.7)**	**0.013**
**Hematocrit (Mean, 95%CI)**	**0.39 (0.37-0.41)**	**0.40 (0.40-0.41)**	**0.030**	0.41 (0.40-0.43)	0.40 (0.39-0.41)	0.063
Platelets – baseline (Mean x10^9/L, 95%CI)	223.7 (200.2-247.3)	223.4 (215.1-231.6)	0.99	237.3 (214.7-259.9)	224.1 (213.6-234.5)	0.42
Partial Thromboplastin Time (Mean seconds, 95%CI)	27.5 (25.4-29.6)	28.2 (27.2-29.2)	0.83	29.3 (27.1-31.4)	28.0 (27.1-28.9)	0.18
International Normalized Ratio	1.1 (1.0-1.1)	1.1 (1.1-1.2)	0.59	1.1 (1.0-1.1)	1.1 (1.1-1.2)	0.20
**Glucose (Mean mmol/L, 95%CI)**	7.2 (6.5-8.0)	7.0 (6.7-7.2)	0.56	**7.5 (6.9-8.1)**	**8.5 (8.0-9.0)**	**0.019**
Creatinine – baseline (Mean umol/L, 95%CI)	94.0 (84.3-103.7)	86.8 (82.9-90.7)	0.068	84.7 (77.9-91.4)	89.8 (85.2-94.4)	0.38
**Glomerular Filtration Rate (Mean, 95%CI)**	**63.5 (56.8-70.1)**	**72.5 (69.8-75.3)**	**0.019**	74.7 (69.0-80.4)	70.4 (67.2-73.5)	0.18
Stroke Mechanism (%)			0.26			0.39
- Cardioembolic	32/41 (78.1)	153/245(62.5)		28/65 (43.1)	118/215 (54.9)	
- Large artery	2/41 (4.9)	42/245 (17.1)		21/65 (32.3)	50/215 (23.3)	
- ESUS	3/41 (7.3)	29/245 (11.8)		10/65 (15.4)	27/215 (12.6)	
- Undetermined	4/41 (9.8)	21/245 (8.6)		6/65 (9.2)	20/215 (9.3)	
**Baseline deficits and stroke locations**						
Baseline NIHSS (mean, 95%CI)	15.2 (13.6-16.9)	14.9 (14.2-15.6)	0.65	17 (15.5-18.5)	18.7 (18.0-19.3)	0.082
Altered level of consciousness (%)*	28/40 (70.0)	141/245 (57.6)	0.17	42/65 (64.6)	155/213 (72.8)	0.22
**Gaze palsy (%)***	32/41 (78.1)	155/245(63.3)	0.077	**48/65 (73.9)**	**191/216 (88.4)**	**0.009**
Hemianopia (%)*	25/40 (62.5)	136/245(55.5)	0.49	47/65 (72.3)	171/213 (80.3)	0.17
Hemiplegia (%)*	26/41 (63.4)	187/245(76.3)	0.085	53/65 (81.5)	193/216 (89.4)	0.13
Ataxia (%)*	2/39 (5.1)	18/243 (7.4)	1.00	5/64 (7.8)	8/210 (3.8)	0.19
Hemianesthesia (%)*	5/40 (12.5)	50/245 (20.4)	0.29	22/65 (33.9)	77/213 (36.2)	0.77
Any aphasia (%)*	28/40 (70.0)	129/245(52.7)	0.058	36/65 (55.4)	131/213 (61.5)	0.39
Any dysarthria (%)*	34/39 (87.2)	213/244(87.3)	1.00	53/64 (82.8)	167/209 (79.9)	0.72
Any neglect (%)*	18/40 (45.0)	138/245(56.3)	0.23	40/65 (61.5)	131/213 (61.5)	1.00
Left vs Right (%)	22/41 (53.7)	118/245(48.2)	0.61	40/65 (61.5)	116/215 (54.0)	0.32
ASPECTS score (mean, 95%CI)	8.5 (8.2-8.8)	8.4 (8.3-8.6)	0.89	6.9 (6.4-7.4)	7.1 (6.8-7.3)	0.51
**Involves:**<br>Caudate (%)	11/41 (26.8)	50/241 (20.8)	0.41	24/65 (36.9)	70/214 (32.7)	0.55
Lentiform nucleus(%)	27/41 (65.9)	157/241(65.2)	1.00	47/65 (72.3)	170/214 (79.4)	0.24
Insula (%)	20/41 (48.8)	130/241(53.9)	0.61	56/65 (86.2)	175/214 (81.8)	0.46
Internal capsule (%)	1/41 (2.4)	0/241	0.15	0/65	1/214 (0.47)	1.00
M1 (%)	0/41	3/241 (1.2)	1.00	11/65 (16.90	24/214 (11.2)	0.28
M2 (%)	1/41 (2.4)	18/241 (7.5)	0.33	12/65 (18.5)	50/214 (23.4)	0.50
M3 (%)	0/41	1/241 (0.41)	1.00	9/65 (13.9)	23/214 (10.8)	0.51
M4 (%)	1/41 (2.4)	6/241 (2.5)	1.00	17/65 (26.2)	46/214 (21.5)	0.50
M5 (%)	0/41	8/241 (3.3)	0.61	16/65 (24.6)	47/214 (22.0)	0.74

© 2021 Ganesh A et al. *JAMA Network Open.*

M6 (%)	0/41	3/241 (1.2)	1.00	9/65 (13.9)	21/214 (9.8)	0.37
Any M1-M6 (%)	2/41 (4.9)	28/241 (11.6)	0.28	32/65 (49.2)	84/214 (39.3)	0.15
**Deep vs cortical** (%)			0.84			0.36
Deep (thalamus or basal ganglia)	14/41 (34.1)	90/241 (37.3)		2/65 (3.1)	2/214 (0.9)	
Superficial (cortical)	5/41 (12.2)	30/241 (12.4)		4/65 (6.2)	12/214 (5.6)	
Both deep & cortical	22/41 (53.7)	121/241(50.2)		59/65 (90.8)	200/214 (93.5)	

ASPECTS – Alberta Stroke Program Early Computed Tomography Score, ESUS – Embolic Stroke of Undetermined Source, mRS – Modified Rankin Scale, NIHSS – National Institutes of Health Stroke Scale Score, SBP – Systolic Blood Pressure, DBP – Diastolic BP

* Neurological deficits at presentation were coded based on components of the baseline NIHSS assessment: altered level of consciousness as a total score≥1 on NIHSS Q1a-c, gaze palsy as score≥1 on NIHSS Q2, hemianopia as score≥1 on NIHSS Q3, hemiplegia as total score≥5 on NIHSS Q4-6 (either the right on Q5-6 or left on Q5-6), ataxia as score≥1 on NIHSS Q7, hemianesthesia as score≥2 on NIHSS Q8, aphasia as score≥1 on NIHSS Q9, dysarthria as score≥1 on NIHSS Q10, and neglect as score≥1 on NIHSS Q11. Whereas discrepant cases with large-PIV seemed less likely to have any gaze palsy at baseline versus non-discrepant cases, there was no difference in the presence of severe gaze palsy (score=2 on NIHSS Q2, 38.5% non-discrepant vs 52.1% discrepant, p=0.065). Both for small-PIV and large-PIV groups, there were no differences between discrepant and non-discrepant cases when we defined the deficits differently including: severe loss of consciousness (score≥3 on NIHSS Q1a-c), severe gaze palsy/deviation (score=2 on NIHSS Q2), severe hemianopia (score≥2 on NIHSS Q3), hemiparesis (score≥2 on NIHSS Q6), severe ataxia (score≥2 on NIHSS Q7), severe aphasia (score≥2 on NIHSS Q9), severe dysarthria (score=2 on NIHSS Q10), or severe neglect (score=2 on NIHSS Q11).

© 2021 Ganesh A et al. *JAMA Network Open.*

eTable 5. Comparison of Treatment-Related and Posttreatment Factors, Including Serious Adverse Events (SAEs), in Discrepant vs Nondiscrepant Cases

Patients with small follow-up infarct volume or FIV (defined as FIV≤7mL) and poor outcome (defined as 90-day mRS≥3) considered discrepant; patients with small FIV and good outcome (mRS≤2) considered nondiscrepant; patients with large FIV (defined as FIV≥92mL) and good outcome (defined as mRS≤2) considered discrepant; patients with large FIV and bad outcome considered nondiscrepant. Variables significant at p<0.05 are bolded; those also significant with Hochberg correction (p<0.0013) are indicated with asterisk (*).

**Treatment-related or post-treatment factor**	**(A) FIV≤7mL**			**(B) FIV≥92mL**		
	**Discrepant**	**Non-discrepant**	**P-value**	**Discrepant**	**Non-discrepant**	**P-value**
**Treatments Received**						
Intervention (NA1) vs Control (%)	25/41 (61.0)	118/245 (48.2)	0.18	27/65 (41.5)	99/216 (45.8)	0.57
IV alteplase given (%)	28/41 (68.3)	147/245 (60.0)	0.39	41/65 (63.1)	112/216 (51.9)	0.12
Onset-to-groin-puncture time, minutes (mean, 95%CI)	213.0 (168.6-257.3)	202.4 (184.6-220.2)	0.41	266.2 (220.2-312.3)	282.4 (258.8-306.0)	0.22
Onset-to-reperfusion time, minutes (mean, 95%CI)	242.0 (195.9-288.1)	232.9 (214.4-251.5)	0.34	299.0 (250.7-347.2)	310.7 (283.6-337.8)	0.25
**Onset-to-needle time, minutes** (mean,95%CI)	**139.3 (105.3-173.2)**	**103.7 (95.6-111.8)**	**0.016**	116.2 (94.6-137.8)	122.9 (110.9-134.9)	0.36
Reperfusion quality: modified Thrombolysis in Cerebral Infarction mTICI 2b-3 (%)	37/41 (90.2)	231/242 (95.5)	0.25	54/65 (83.1)	153/209 (73.2)	0.14
**General anesthesia (%)**	11/41 (26.8)	40/242 (16.5)	0.20	**7/65 (10.8)**	**61/213 (28.6)**	**0.009**
Length of in-hospital rehabilitation (mean days, 95%CI)	29.5 (21.3-37.7)	20.7 (16.9-24.5)	**0.032**	26.6 (19.6-33.6)	36.2 (28.8-43.6)	0.073
**Post-treatment Parameters**						
**SBP at 24-hours** (mean mmHg, 95%CI)	128.0(125.6-130.3)	129.7(123.3-136.0)	0.69	**126.0(121.5-130.5)**	**135.0 (132.4-137.7)**	**0.0009***
**DBP at 24-hours** (Mean, 95%CI)	66.3 (62.2-70.5)	68.8 (67.3-70.3)	0.098	**64.9 (62.0-67.8)**	**68.6 (66.8-70.4)**	**0.041**
**Heart Rate at 24-hours** (Mean, 95%CI)	79.9 (74.3-85.5)	76.0 (74.1-78.0)	0.20	**75.3 (72.0-78.6)**	**81.3 (79.0-83.5)**	**0.0088**
**Hemoglobin at 24-hours** (Mean g/L, 95%CI)	**111.0 (105.3-116.7)**	**122.0 (119.9-124.2)**	**0.0004***	**126.2 (122.0-130.4)**	**118.3 (115.8-120.8)**	**0.0013***
**Hematocrit at 24-hours**	**0.34 (0.32-0.36)**	**0.37 (0.36-0.37)**	**0.0032**	**0.37 (0.36-0.39)**	**0.35 (0.35-0.36)**	**0.0054**
Platelets at 24-hours (Mean x10^9/L, 95%CI)	200.7(179.4-222.0)	202.6 (194.8-210.4)	0.87	212.4(194.2-230.7)	200.0 (191.3-208.7)	0.41
**Glucose at 24-hours (Mean, 95%CI)**	7.0 (6.2-7.7)	6.3 (6.1-6.5)	0.063	**6.7 (6.2-7.2)**	**8.4 (7.9-8.8)**	**<0.0001***
Creatinine at 24-hours (Mean umol/L, 95%CI)	84.1 (74.1-94.0)	77.3 (74.1-80.5)	0.19	75.2 (69.4-80.9)	80.9 (76.5-85.4)	0.28
Estimated Glomerular Filtration Rate at 24-hours (Mean, 95%CI)	71.1 (64.4-77.8)	79.1 (76.1-82.1)	0.056	83.5 (77.3-89.8)	78.5 (75.2-81.8)	0.12
**24-hour NIHSS** (mean, 95%CI)	**6.5 (4.7-8.2)**	**2.5 (2.1-3.0)**	**<0.0001***	**9.2 (7.5-10.9)**	**18.4 (17.5-19.4)**	**<0.0001***
**48-hour NIHSS**	**5.8 (4.3-7.2)**	**1.8 (1.3-2.2)**	**<0.0001***	**7.2 (5.6-8.7)**	**18.2 (17.2-**	**<0.0001***

© 2021 Ganesh A et al. *JAMA Network Open.*

(mean, 95%CI)					**19.2)**	
**Serious Adverse Events (SAEs)**						
**Any SAE (%)**	**20/41 (48.9)**	**53/245 (21.6)**	**0.001***	**13/65 (20.3)**	**139/216 (64.7)**	**<0.001***
**Likely index stroke-related SAEs, not clearly EVT-attributable (%)**	2/41 (4.9)	1/245 (0.4)	0.055	**4/65 (6.2)**	**61/216 (28.2)**	**<0.001***
**Progression of index stroke**	1/41 (2.4)	0	0.14	**3/65 (4.6)**	**59/216 (27.3)**	**<0.001***
Functional decline or failure to thrive	0	0		0/65	1/216 (0.5)	1.00
Seizures	1/41 (2.4)	1/245 (0.4)	0.27	1/64 (1.6)	1/215 (0.5)	0.41
**EVT/thrombolysis-related SAEs (%)**	7/41 (17.1)	21/245 (8.6)	0.095	**12/65 (18.5)**	**70/216 (32.4)**	**0.030**
**Symptomatic intracerebral hemorrhage (sICH)**	0	2/245 (0.8)	1.00	**2/65 (3.1)**	**29/216 (13.4)**	**0.022**
Intra-procedural complications	3/41 (7.3)	10/245 (4.1)	0.41	8/65 (12.3)	34/216 (15.7)	0.56
- Dissection	1/41 (2.4)	6/244 (2.5)	1.00	4/65 (6.2)	9/215 (4.2)	0.51
- Embolism	0/41	6/244 (2.5)	0.60	5/65 (7.7)	21/215 (9.8)	0.81
- Perforation	2/41 (4.9)	3/244 (1.2)	0.15	0/65	11/215 (5.1)	0.073
- Iliofemoral pseudoaneurysm	0	0		0/65	1/216 (0.5)	1.00
**Infarct in New Territory**	**5/41 (12.2)**	**8/243 (3.3)**	**0.026**	4/64 (6.3)	31/209 (14.8)	0.088
Stent/intracranial re-occlusion	0	0		0/65	1/216 (0.5)	1.00
**Secondary prevention-related SAEs (%)**	7/41 (17.1)	19/245 (7.8)	0.074	3/65 (4.6)	18/216 (8.3)	0.43
**Recurrent stroke**	**5/41 (12.2)**	**6/245 (2.5)**	**0.011**	0/65	8/216 (3.7)	0.21
Acute Coronary Syndrome	1/41 (2.4)	4/245 (1.6)	0.54	1/64 (1.6)	4/215 (1.9)	1.00
Chest Pain or other pain	0	0		0/65	1/216 (0.5)	1.00
Limb or Bowel Ischemia	1/41 (2.4)	3/245 (1.2)	0.46	1/64 (1.6)	3/215 (1.4)	1.00
Central Retinal Artery Occlusion	0	1/245 (0.4)	1.00	0	0	
Transient Ischemic Attack	1/41 (2.4)	5/245 (2.0)	1.00	1/65 (1.5)	0/216	0.23
Cardiac Arrest	0	1/245 (0.4)	1.00	0/65	3/216 (1.4)	1.00
**Systemic SAEs, potentially stroke unit care-related (%)**	**13/41 (31.7)**	**17/245 (6.9)**	**<0.001***	**5/65 (7.7)**	**57/216 (26.4)**	**0.001***
**Pneumonia (all)**	**3/41 (7.3)**	**2/245 (0.8)**	**0.022**	**0/65**	**17/216 (7.9)**	**0.015**
**Aspiration Pneumonia**	1/41 (2.4)	1/245 (0.4)	0.27	**0/64**	**15/215 (7.0)**	**0.026**
Respiratory Failure	1/41 (2.4)	0	0.14	0/64	12/215 (5.6)	0.074
Dysphagia	0	0		0/65	1/216 (0.5)	1.00
Deep vein thrombosis or pulmonary embolism	1/41 (2.4)	1/245 (0.4)	0.27	0/65	3/216 (1.4)	1.00
Extracranial hemorrhage	2/41 (4.9)	4/245 (1.6)	0.21	0/64	4/215 (1.9)	0.58
Gastrointestinal Bleed	1/41 (2.4)	1/245 (0.4)	0.27	1/64 (1.6)	1/215 (0.5)	0.41
Anemia	1/41 (2.4)	1/245 (0.4)	0.27	0/65	1/216 (0.5)	1.00
**Congestive heart failure**	**3/41 (7.3)**	**2/245 (0.8)**	**0.022**	0/65	1/216 (0.5)	1.00
Hypotension	1/41 (2.4)	0/245	0.14	0/65	2/216 (0.9)	1.00
Sepsis	1/41 (2.4)	2/245 (0.8)	0.37	1/65 (1.5)	10/216 (4.6)	0.47
Urinary Tract Infection	1/41 (2.4)	2/245 (0.8)	0.37	0/65	6/216 (2.8)	0.34
Other infection	1/41 (2.4)	2/245 (0.8)	0.37	0/65	3/216 (1.4)	1.00
Trauma or injuries	1/41 (2.4)	3/245 (1.2)	0.46	1/65 (1.5)	2/216 (0.9)	0.55

© 2021 Ganesh A et al. *JAMA Network Open.*

Acute Kidney Injury	1/41 (2.4)	0	0.14	0/65	2/216 (0.9)	1.00
Depression	1/41 (2.4)	0	0.14	0/65	1/216 (0.5)	1.00
Delirium or encephalopathy	0	0		1/65 (1.5)	2/216 (0.9)	0.55
Displaced gastrostomy tube	0	0		0	0	
**Miscellaneous/idiosyncratic SAEs (%)**	2/41 (4.9)	7/245 (2.9)	0.62	0/65	3/216 (1.4)	1.00

© 2021 Ganesh A et al. *JAMA Network Open.*

eTable 6. Comparison of Prespecified and Stepwise Multivariable Logistic Regression Models for the Association of Pretreatment Variables Among 1091 Patients and Treatment-Related or Posttreatment Variables Among 1086 Patients in Prespecified Model and 631 Patients in Stepwise Model for the Outcome of mRS Score of 3 or Greater Despite FIV of 7 mL or Less in the ESCAPE-NA1 Trial

In this iteration, the generic serious adverse event (SAE) variable in the pre-specified model has been replaced with the more specific SAEs that differed between discrepant and non-discrepant cases in univariable analyses of the ESCAPE-NA1 dataset. All models were also adjusted for FIV, alteplase, nerinetide, and interaction of alteplase with nerinetide treatment.

**Outcome: FIV≤7mL & mRS≥3**					
**(1) Model with pre-specified variables based on ESCAPE analysis**			**(2) Model with variables selected by stepwise multivariable logistic regression**		
**Variable**	**aOR(95%CI)**	**P>|z|**	**Variable**	**aOR(95%CI)**	**P>|z|**
**A. PRE-TREATMENT FACTORS**					
Age (per year)	1.08(1.04-1.13)	<0.001	Age (per year)	1.08 (1.04-1.12)	<0.001
Cancer	61.6 (2.91-1304)	0.008			
Vascular risk factors (number)	1.30 (1.06-1.59)	0.013	Vascular risk factors (number)	1.27 (1.04-1.56)	0.018
**AUC (95%CI)**	0.92 (0.89-0.95)	P<0.0001		0.93 (0.90-0.95)	P<0.0001
**Pearson X^2**	344	P=1.00		329	P=1.00
**Hosmer-Lemeshow X^2**	0.37	P=0.95		0.46	P=0.93
**B. TREATMENT/POST-TREATMENT FACTORS**					
INT	6.84 (1.99-23.5)	0.002	INT	8.35 (1.81-38.5)	0.006
Recurrent stroke	8.33 (2.22-31.2)	0.002	Recurrent stroke	12.9 (1.96-84.3)	0.008
Pneumonia	12.1 (2.18-66.7)	0.004	Pneumonia	9.0 (0.73-109)	0.087
CHF	10.7 (1.69-67.5)	0.012	Hemoglobin at 24-hours (per g/L increase)	0.96 (0.93-0.99)	0.003
**AUC (95%CI)**	0.91 (0.88-0.94)	P<0.0001		0.93 (0.90-0.96)	<0.0001
**Pearson X^2**	240	P=1.00		142	P=1.00
**Hosmer-Lemeshow X^2**	0.76	P=0.86		0.22	P=0.97

No difference in AUC for pre-treatment factor models 1 and 2, P=0.66.
No difference in AUC for treatment/post-treatment factor models 1 and 2, P=0.23.
aOR=adjusted odds ratio; AUC=Area under the receiver-operating-characteristics curve; CHF=congestive heart failure (new or exacerbation); CI=confidence interval; INT=Infarct in New Territory; mRS=modified Rankin Scale score; FIV=follow-up infarct volume

© 2021 Ganesh A et al. *JAMA Network Open.*

eTable 7. Comparison of Prespecified and Stepwise Multivariable Logistic Regression Models for the Association of Pretreatment Variables Among 1094 Patients and Treatment-Related or Posttreatment Variables Among 1089 Patients for the Outcome of mRS Score of 4 or Greater Despite FIV of 7 mL or Less in the ESCAPE-NA1 Trial

All models were also adjusted for FIV, alteplase, nerinetide, and interaction of alteplase with nerinetide treatment.

**Outcome: FIV≤7mL & mRS≥4**					
**(1) Model with pre-specified variables based on ESCAPE analysis**			**(2) Model with variables selected by stepwise multivariable logistic regression**		
**Variable**	**aOR(95%CI)**	**P>|z|**	**Variable**	**aOR(95%CI)**	**P>|z|**
**A. PRE-TREATMENT FACTORS**					
Age (per year)	1.08 (1.03-1.13)	0.003	Age (per year)	1.08 (1.03-1.13)	0.003
Cancer	16.5 (1.08-251)	0.044	Cancer	16.5 (1.08-251)	0.044
Vascular risk factors (number)	1.47 (1.14-1.88)	0.002	Vascular risk factors (number)	1.47 (1.14-1.88)	0.002
**AUC (95%CI)**	0.93 (0.91-0.96)	P<0.0001		0.93 (0.91-0.96)	P<0.0001
**Pearson X^2**	297	P=1.00		297	P=1.00
**Hosmer-Lemeshow X^2**	0.38	P=0.94		0.38	P=0.94
**B. TREATMENT/POST-TREATMENT FACTORS**					
INT	4.31 (0.84-22.0)	0.080			
Recurrent stroke	7.34 (1.62-33.3)	0.010	Recurrent stroke	6.80 (1.52-30.5)	0.012
Pneumonia	23.2 (5.08-106)	<0.001	Pneumonia	19.9 (4.54-87.5)	<0.001
CHF	25.1 (4.11-154)	<0.001	CHF	22.2 (3.76-132)	0.001
**AUC (95%CI)**	0.90 (0.86-0.94)	P<0.0001		0.91 (0.87-0.95)	P<0.0001
**Pearson X^2**	230	P=1.00		281	P=1.00
**Hosmer-Lemeshow X^2**	1.36	P=0.71		0.43	P=0.93

No difference in AUC for pre-treatment factor models 1 and 2; P=1.00.
No difference in AUC for treatment/post-treatment factor models 1 and 2; P=0.15.

aOR=adjusted odds ratio; AUC=Area under the receiver-operating-characteristics curve; CHF=congestive heart failure (new or exacerbation); CI=confidence interval; INT=Infarct in New Territory; mRS=modified Rankin Scale score; FIV=follow-up infarct volume

© 2021 Ganesh A et al. *JAMA Network Open.*

eTable 8. Comparison of Prespecified and Stepwise Multivariable Logistic Regression Models for the Association of Treatment-Related or Posttreatment Variables Among 918 Patients for the Outcome of mRS Score of 3 or Greater Despite FIV of 7 mL or Less in the ESCAPE-NA1 Trial With 24-Hour or 48-hour NIHSS Included as a Posttreatment Variable

In this iteration, we permitted the 24- or 48-hour NIHSS to be included as a post-treatment variable**.** All models were also adjusted for FIV, alteplase, nerinetide, and interaction of alteplase with nerinetide treatment.

**Outcome: FIV≤7mL & mRS≥3**					
**(1) Model with pre-specified variables based on ESCAPE analysis**			**(2) Model with variables selected by stepwise multivariable logistic regression, and with onset-to-alteplase time included (limiting N)**		
**Variable**	**aOR(95%CI)**	**P>|z|**	**Variable**	**aOR(95%CI)**	**P>|z|**
INT	9.25 (1.62-53.0)	0.012			
Recurrent stroke	7.39 (1.39-39.3)	0.019	Recurrent stroke	25.1 (3.21-196)	0.002
Pneumonia	8.93 (1.52-52.5)	0.015			
CHF	4.42 (0.45-43.7)	0.20	Hemoglobin at 24-hours (per g/L increase)	0.94 (0.91-0.98)	0.001
48-hour NIHSS (per unit increase)	1.21 (1.11-1.32)	<0.001	24-hour NIHSS (per unit increase)	1.23 (1.11-1.36)	<0.001
N	920			616	
**AUC (95%CI)**	0.95 (0.92-0.97)	P<0.0001		0.95 (0.93-0.98)	P<0.0001
**Pearson X^2**	147	P=1.00		115	P=1.00
**Hosmer-Lemeshow X^2**	1.49	P=0.69		0.84	P=0.84
			**(2) Model with variables selected by stepwise multivariable logistic regression, without onset-to-alteplase time**		
			**Variable**	**aOR(95%CI)**	**P>|z|**
			INT	9.58 (1.65-55.4)	0.012
			Recurrent Stroke	7.27 (1.23-42.9)	0.029
			Systemic SAEs, potentially stroke unit care-related	6.63 (2.52-17.5)	<0.001
			48-hour NIHSS (per unit increase)	1.22 (1.12-1.34)	<0.001
			N	920	
			**AUC (95%CI)**	0.95 (0.93-0.97)	P<0.0001
			**Pearson X^2**	159	P=1.00
			**Hosmer-Lemeshow X^2**	1.08	P=0.78

No difference in AUC for model 1 vs 2, p=0.51; or between model 1 vs 3, p=0.31.

aOR=adjusted odds ratio; AUC=Area under the receiver-operating-characteristics curve; CHF=congestive heart failure (new or exacerbation); CI=confidence interval; INT=Infarct in New Territory; mRS=modified Rankin Scale score; FIV=follow-up infarct volume

© 2021 Ganesh A et al. *JAMA Network Open.*

eTable 9. Comparison of Prespecified and Stepwise Multivariable Logistic Regression Models for the Association of Pretreatment Variables Among 254 Patients and Treatment-Related or Posttreatment Variables Among 256 Patients for Outcome of mRS Score of 4 or Greater Despite FIV of 7 mL or Less in the ESCAPE-NA1 Trial, Restricted to Patients With MRI for FIV Determination
<u>All models were also adjusted for FIV, alteplase, nerinetide, and interaction of alteplase with nerinetide treatment.</u>

**Outcome: FIV≤7mL & mRS≥3**					
**(1) Model with pre-specified variables based on ESCAPE analysis**			**(2) Model with variables selected by stepwise multivariable logistic regression**		
**Variable**	**aOR(95%CI)**	**P>|z|**	**Variable**	**aOR(95%CI)**	**P>|z|**
**A. PRE-TREATMENT FACTORS**					
Age (per year)	1.15 (1.03-1.29)	0.016	Age (per year)	1.15 (1.02-1.30)	0.028
Cancer	Perfect predictor	n=2 dropped	Baseline hemoglobin (per g/L increase)	0.93 (0.88-0.99)	0.026
Vascular risk factors (number)	1.16 (0.68-1.98)	0.59			
**AUC (95%CI)**	0.89 (0.80-0.98)	P<0.0001		0.92 (0.85-0.98)	P<0.0001
**Pearson X^2**	72.8	P=1.00		38.7	P=1.00
**Hosmer-Lemeshow X^2**	0.25	P=0.97		0.04	P=1.00
**B. TREATMENT/POST-TREATMENT FACTORS**					
INT	20.1 (2.43-167)	0.005	INT	8.47 (0.69-104)	0.094
Recurrent stroke	11.6 (0.84-161)	0.068	Recurrent stroke	51.9 (1.40-1920)	0.032
Pneumonia	86.2 (0.43-17111)	0.099	Hemoglobin at 24-hours (per g/L increase)	0.88 (0.80-0.98)	0.017
CHF	Dropped as no discrepant case had CHF	n=3			
**AUC (95%CI)**	0.87 (0.68-0.99)	P<0.0001		0.62 (0.10-0.99)	P<0.0001
**Pearson X^2**	90.7	P=1.00		0.00	P=1.00
**Hosmer-Lemeshow X^2**	1.48	P=0.69		0.00	P=1.00

AUC no different for pre-treatment factor model 1 vs 2, p=0.35.
AUC no different for treatment/post-treatment factor model 2 vs 1, p=0.14.

aOR=adjusted odds ratio; AUC=Area under the receiver-operating-characteristics curve; CHF=congestive heart failure (new or exacerbation); CI=confidence interval; INT=Infarct in New Territory; mRS=modified Rankin Scale score; FIV=follow-up infarct volume

© 2021 Ganesh A et al. *JAMA Network Open.*

eTable 10. Comparison of Prespecified and Stepwise Multivariable Logistic Regression Models for the Association of Pretreatment Variables Among 1091 Patients and Treatment-Related or Posttreatment Variables Among 1082 Patients for the Outcome of mRS Score of 2 or Less Despite FIV of 92 mL or Greater in the ESCAPE-NA1 Trial

In this iteration, the generic serious adverse event (SAE) variable in the pre-specified model has been replaced with the more specific SAEs that differed between discrepant and non-discrepant cases in univariable analyses of the ESCAPE-NA1 dataset. All models were also adjusted for FIV, alteplase, nerinetide, and interaction of alteplase with nerinetide treatment.

**Outcome: FIV≥92mL & mRS≤2**					
**(1) Model with pre-specified variables based on ESCAPE analysis**			**(2) Model with variables selected by stepwise multivariable logistic regression**		
**Variable**	**aOR(95%CI)**	**P>|z|**	**Variable**	**aOR(95%CI)**	**P>|z|**
**A. PRE-TREATMENT FACTORS**					
Age (per year)	0.97 (0.95-0.99)	0.006	Age (per year)	0.98 (0.96-0.99)	0.017
Vascular risk factors (number)	0.99 (0.84-1.16)	0.89	Diabetes mellitus	0.38 (0.16-0.92)	0.032
			Baseline hemoglobin (per g/L increase)	1.02 (1.00-1.03)	0.043
**AUC (95%CI)**	0.76 (0.70-0.82)	P<0.0001		0.76 (0.70-0.82)	P<0.0001
**Pearson X^2**	703	P=1.00		686	P=1.00
**B. TREATMENT/POST-TREATMENT FACTORS**					
Stroke progression	0.03 (0.01-0.14)	<0.001	Stroke progression	0.05 (0.01-0.25)	<0.001
sICH	0.09 (0.02-0.49)	0.006	sICH	0.15 (0.03-0.84)	0.031
Pneumonia	No mismatch case had pneumonia	n=40 dropped	DBP at 24-hours (per mmHg increase)	0.97 (0.94-0.99)	0.003
			Glucose at 24-hours (per mM increase)	0.82 (0.69-0.96)	0.016
			Hemoglobin at 24-hours (per g/L increase)	1.02 (1.01-1.04)	0.009
**AUC (95%CI)**	0.85 (0.80-0.90)	P<0.0001		0.79 (0.72-0.86)	P<0.0001
**Pearson X^2**	822	P=0.86		725	P=0.93
**Hosmer-Lemeshow X^2**	22.5	P=0.65		3.17	P=0.37

No difference in AUC between pre-treatment factor models 1 and 2, P=0.90.

AUC superior for treatment/post-treatment factor model 1 vs 2, p=0.030.

aOR=adjusted odds ratio; AUC=Area under the receiver-operating-characteristics curve; CI=confidence interval; DBP=diastolic blood pressure; mRS=modified Rankin Scale score; FIV=follow-up infarct volume; sICH=symptomatic intracerebral hemorrhage

© 2021 Ganesh A et al. *JAMA Network Open.*

eTable 11. Comparison of Prespecified and Stepwise Multivariable Logistic Regression Models for the Association of Pretreatment Variables Among 1091 Patients and Treatment-Related or Posttreatment Variables Among 1082 Patients for the Outcome of mRS Score of 3 or Less Despite FIV of 92 mL or Greater in the ESCAPE-NA1 Trial
All models were also adjusted for FIV, alteplase, nerinetide, and interaction of alteplase with nerinetide treatment.

**Outcome: FIV≥92mL & mRS≤3**					
**(1) Model with pre-specified variables based on ESCAPE analysis**			**(2) Model with variables selected by stepwise multivariable logistic regression**		
**Variable**	**aOR(95%CI)**	**P>|z|**	**Variable**	**aOR(95%CI)**	**P>|z|**
**A. PRE-TREATMENT FACTORS**					
Age (per year)	0.97 (0.96-0.99)	0.002	Age (per year)	0.98 (0.96-0.99)	0.002
Vascular risk factors (number)	1.02 (0.89-1.15)	0.82	Diabetes mellitus	0.66 (0.37-1.19)	0.17
**AUC (95%CI)**	0.81 (0.78-0.84)	P<0.0001		0.82 (0.79-0.85)	P<0.0001
**Pearson X^2**	688	P=0.99		663	P=1.00
**Hosmer-Lemeshow X^2**	7.05	P=0.07		4.93	P=0.18
**B. TREATMENT/POST-TREATMENT FACTORS**					
Stroke progression	0.02 (0.004-0.07)	<0.001	Stroke progression	0.01 (0.002-0.06)	<0.001
sICH	0.08 (0.02-0.33)	0.001	sICH	0.07 (0.02-0.31)	<0.001
Pneumonia	0.07 (0.01-0.62)	0.017	Pneumonia	0.07 (0.01-0.64)	0.018
			SBP at 24 hours (per mmHg increase)	0.99 (0.98-1.00)	0.090
**AUC (95%CI)**	0.86 (0.82-0.91)	P<0.0001		0.87 (0.83-0.91)	P<0.0001
**Pearson X^2**	149.2	P=0.99		168.8	P=0.97
**Hosmer-Lemeshow X^2**	5.69	P=0.13		6.39	P=0.094

No difference in AUC between pre-treatment factor models 1 and 2, P=0.29.
No difference in AUC between post-treatment factor models 1 and 2, P=0.67.

aOR=adjusted odds ratio; AUC=Area under the receiver-operating-characteristics curve; CI=confidence interval; mRS=modified Rankin Scale score; FIV=follow-up infarct volume; SAE=serious adverse event; SBP=systolic blood pressure; sICH=symptomatic intracerebral hemorrhage

© 2021 Ganesh A et al. *JAMA Network Open.*

eTable 12. Comparison of Prespecified and Stepwise Multivariable Logistic Regression Models for the Association Treatment-Related or Posttreatment Variables Among 924 Patients for the Outcome of mRS Score of 2 or Less Despite FIV of 92 mL or Greater in the ESCAPE-NA1 Trial With 24-Hour or 48-hour NIHSS Included as a Posttreatment Variable
In this iteration, we permitted the 24- or 48-hour NIHSS to be included as a post-treatment variable**.** All models were also adjusted for FIV, alteplase, nerinetide, and interaction of alteplase with nerinetide treatment.

**Outcome: FIV≥92mL & mRS≤2**					
**(1) Model with pre-specified variables based on ESCAPE analysis**			**(2) Model with variables selected by stepwise multivariable logistic regression**		
**Variable**	**aOR(95%CI)**	**P>|z|**	**Variable**	**aOR(95%CI)**	**P>|z|**
Stroke Progression	0.05 (0.006-0.41)	0.005	Stroke progression	0.06 (0.01-0.44)	0.006
sICH	0.13 (0.02-0.86)	0.034	sICH	0.22 (0.03-1.39)	0.11
Pneumonia	No mismatch case had pneumonia	n=38 dropped	SBP at 24-hours (per mmHg increase)	0.98 (0.96-0.996)	0.015
48-hour NIHSS (per unit increase)	0.82 (0.77-0.88)	<0.001	48-hour NIHSS (per unit increase)	0.83 (0.78-0.90)	<0.001
			Hemoglobin at 24-hours (per g/L increase)	1.02 (1.004-1.04)	0.014
**AUC (95%CI)**	0.85 (0.78-0.92)	P<0.0001		0.83 (0.76-0.90)	P<0.0001
**Pearson X^2**	119.6	P=0.996		156.2	P=0.70
**Hosmer-Lemeshow X^2**	4.75	P=0.19		1.68	P=0.82

No difference in AUC between post-treatment models 1 and 2, p=0.078.

aOR=adjusted odds ratio; AUC=Area under the receiver-operating-characteristics curve; CI=confidence interval; INT=Infarct in New Territory; mRS=modified Rankin Scale score; NIHSS=National Institutes of Health Stroke Scale score; FIV=follow-up infarct volume; SBP=systolic blood pressure; sICH=symptomatic intracerebral hemorrhage.

© 2021 Ganesh A et al. *JAMA Network Open.*

eFigure 1. Proportions of Patients in the ESCAPE-NA1 Trial With Discrepancies Between FIV and 90-day mRS Score, Based on the Definitions Used in this Study

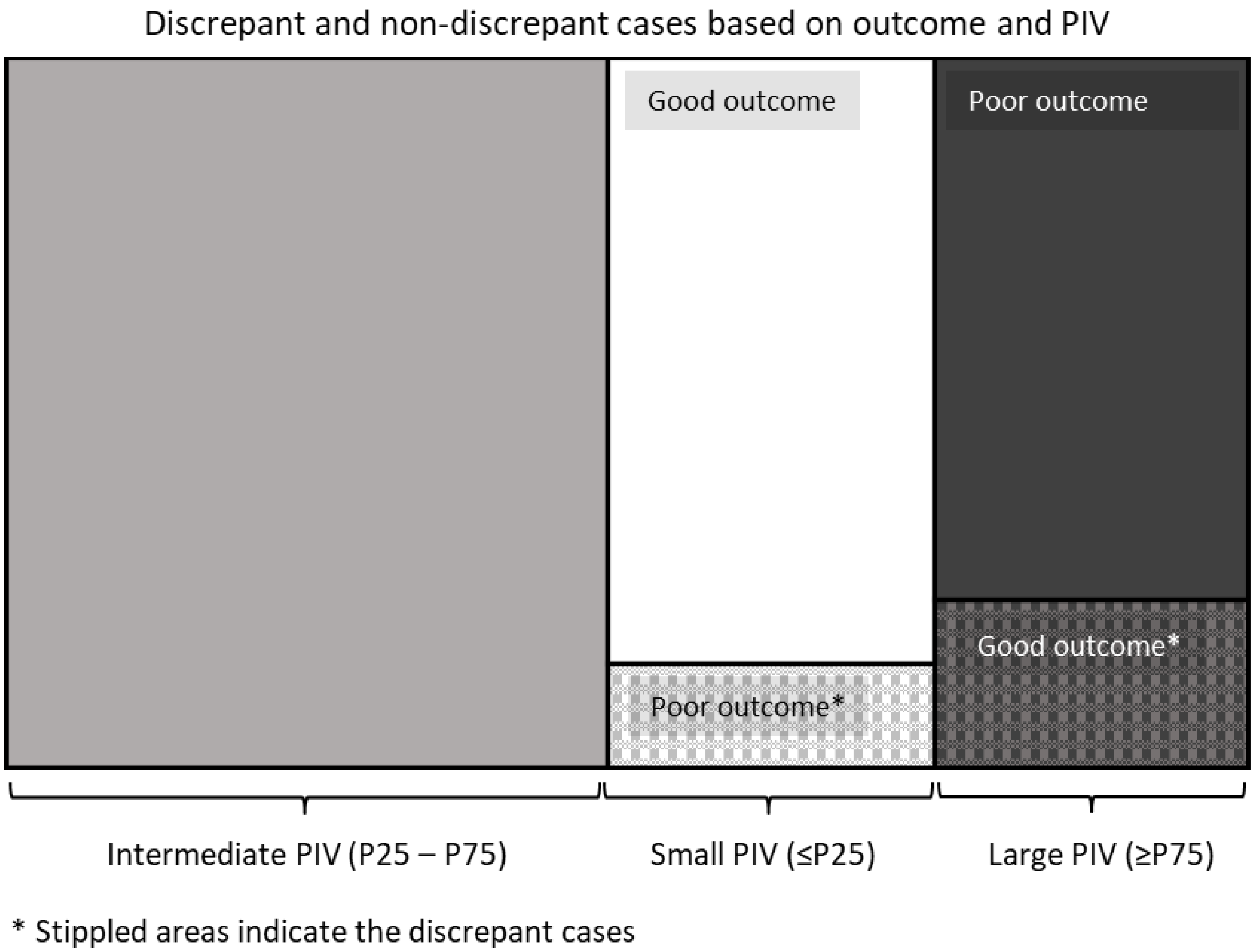

© 2021 Ganesh A et al. *JAMA Network Open.*

eFigure 2. Proportion of Patients in Each Age Group With 90-Day mRS Score of 3 or Greater Despite Small FIV and mRS Score of 2 or Less Despite Large FIV

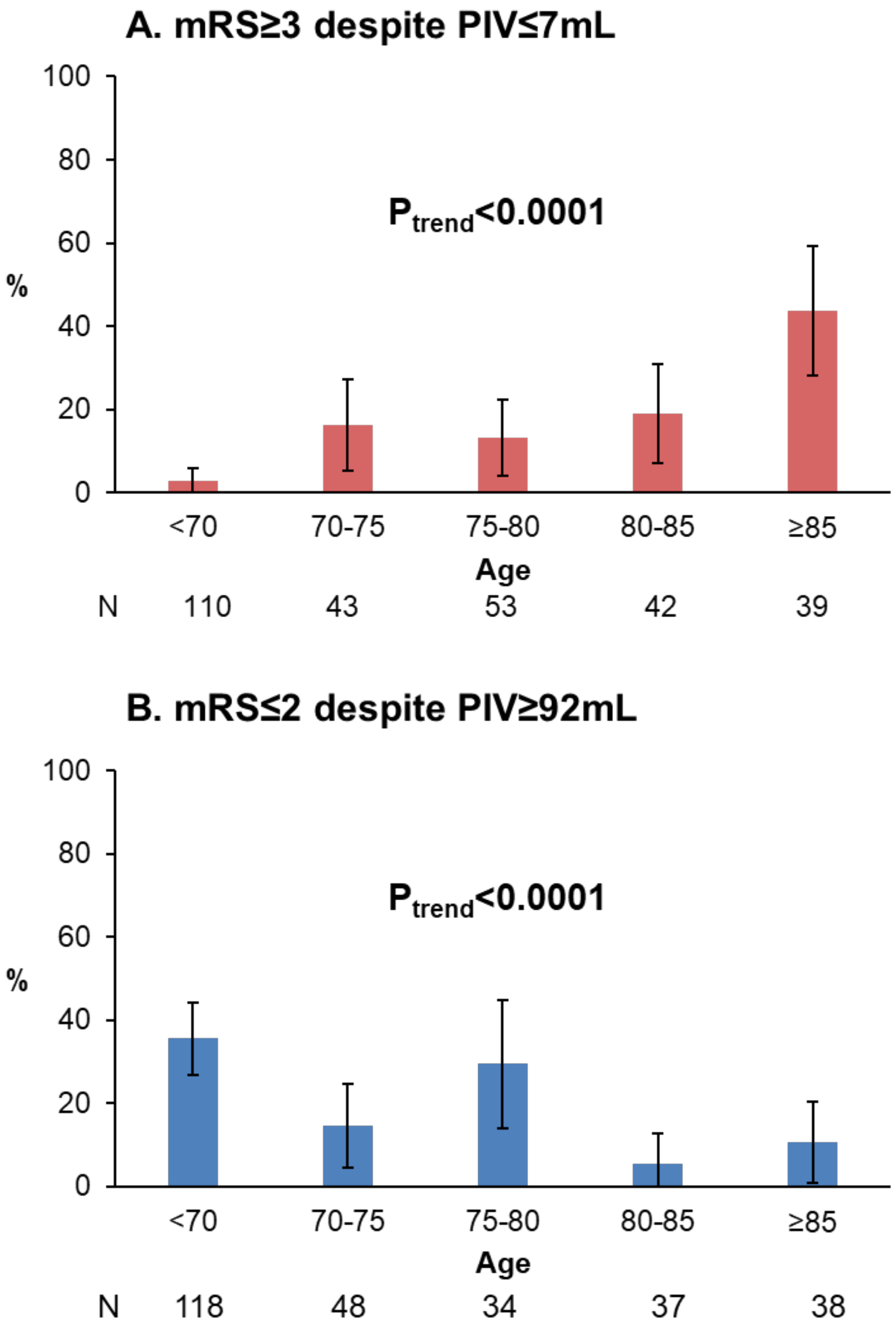

Bars represent 95% confidence intervals. The total number of individuals in each group is shown below each graph. P-values are from the Fisher's exact test. Older patients were more likely to have a poor functional outcome despite a small FIV, whereas younger patients were more likely to have a good outcome despite a large FIV.

© 2021 Ganesh A et al. *JAMA Network Open.*
