# Supplementary material for: Assessment of Discrepancies Between Follow-up Infarct Volume and 90-Day Outcomes Among Patients With Ischemic Stroke Who Received Endovascular Therapy
Source: JAMA Netw Open. 2021 Nov 5;4(11):e2132376. doi: 10.1001/jamanetworkopen.2021.32376 (PMC8571657; doi:10.1001/jamanetworkopen.2021.32376)
Supplement: Supplement 2. — Nonauthor Collaborators [file jamanetwopen-e2132376-s002.pdf]

*Indicates required information. Only first name, last name, and suffix will appear in PubMed.

| *Group Name(s): ESCAPE-NA1 Trial Investigators | | | | | | | |
|---|---|---|---|---|---|---|---|
| *First Name and Middle Initial(s) | *Last Name | *Suffix (eg, Jr, III) | Academic Degrees | Institution | Location (city, state/province, country) | Role or Contribution, eg, chair, principal investigator | Group (if more than 1 Group listed in the byline) and/or Subgroup (eg, Steering Committee) |
| Philip A. | Barber | | MD | Foothills Medical Centre, University of Calgary | Calgary, AB, Canada | Collaborator | |
| Eric E. | Smith | | MD | Foothills Medical Centre, University of Calgary | Calgary, AB, Canada | Collaborator | |
| Simerpreet | Bal | | MD | Foothills Medical Centre, University of Calgary | Calgary, AB, Canada | Collaborator | |
| Suresh | Subramanian | | MD | Foothills Medical Centre, University of Calgary | Calgary, AB, Canada | Collaborator | |
| Steven | Peters | | MD | Foothills Medical Centre, University of Calgary | Calgary, AB, Canada | Collaborator | |
| Philippe | Couillard | | MD | Foothills Medical Centre, University of Calgary | Calgary, AB, Canada | Collaborator | |
| Gary | Klein | | MD | Foothills Medical Centre, University of Calgary | Calgary, AB, Canada | Collaborator | |
| Peter | Stys | | MD | Foothills Medical Centre, University of Calgary | Calgary, AB, Canada | Collaborator | |
| Shelagh B. | Coutts | | MD | Foothills Medical Centre, University of Calgary | Calgary, AB, Canada | Collaborator | |
| John | Wong | | MD | Foothills Medical Centre, University of Calgary | Calgary, AB, Canada | Collaborator | |
| Alim | Mitha | | MD | Foothills Medical Centre, University of Calgary | Calgary, AB, Canada | Collaborator | |
| Muneer | Eesa | | MD | Foothills Medical Centre, University of Calgary | Calgary, AB, Canada | Collaborator | |
| William | Morrish | | MD | Foothills Medical Centre, University of Calgary | Calgary, AB, Canada | Collaborator | |
| Saad | Alqatani | | MD | Foothills Medical Centre, University of Calgary | Calgary, AB, Canada | Collaborator | |
| Nima | Kashani | | MD | Foothills Medical Centre, University of Calgary | Calgary, AB, Canada | Collaborator | |
| Prasanna | Venkatesan | | MD | Foothills Medical Centre, University of Calgary | Calgary, AB, Canada | Collaborator | |
| Ericka | Teleg | | MD | Foothills Medical Centre, University of Calgary | Calgary, AB, Canada | Collaborator | |

*Indicates required information. Only first name, last name, and suffix will appear in PubMed.

| *First Name and Middle Initial(s) | *Last Name | *Suffix (eg, Jr, III) | Academic Degrees | Institution | Location (city, state/province, country) | Role or Contribution, eg, chair, principal investigator | Group (if more than 1 Group listed in the byline) and/or Subgroup (eg, Steering Committee) |
|---|---|---|---|---|---|---|---|
| Amith | Sitaram | | MD | Foothills Medical Centre, University of Calgary | Calgary, AB, Canada | Collaborator | |
| Brett | Graham | | MD | Foothills Medical Centre, University of Calgary | Calgary, AB, Canada | Collaborator | |
| Stephen van | Gaal | | MD | Foothills Medical Centre, University of Calgary | Calgary, AB, Canada | Collaborator | |
| Aimen | Moussaddy | | MD | Foothills Medical Centre, University of Calgary | Calgary, AB, Canada | Collaborator | |
| Debabrata | Chakraborty | | MD | Foothills Medical Centre, University of Calgary | Calgary, AB, Canada | Collaborator | |
| Nicholar | Maraj | | MD | Foothills Medical Centre, University of Calgary | Calgary, AB, Canada | Collaborator | |
| Andrew | Lockey | | MD | Foothills Medical Centre, University of Calgary | Calgary, AB, Canada | Collaborator | |
| Shuo | Chen | | MD | Foothills Medical Centre, University of Calgary | Calgary, AB, Canada | Collaborator | |
| Ravinder | Singh | | MD | Foothills Medical Centre, University of Calgary | Calgary, AB, Canada | Collaborator | |
| Abdulaziz Sulaiman | Alsultan | | MD | Foothills Medical Centre, University of Calgary | Calgary, AB, Canada | Collaborator | |
| Ria | Asunsian | | MD | Foothills Medical Centre, University of Calgary | Calgary, AB, Canada | Collaborator | |
| Dominic | Tse | | MD | Foothills Medical Centre, University of Calgary | Calgary, AB, Canada | Collaborator | |
| Darshan | Doshi | | MD | Foothills Medical Centre, University of Calgary | Calgary, AB, Canada | Collaborator | |
| Ondrej | Volny | | MD | Foothills Medical Centre, University of Calgary | Calgary, AB, Canada | Collaborator | |
| Piyush | Ojha | | MD | Foothills Medical Centre, University of Calgary | Calgary, AB, Canada | Collaborator | |
| Ankur | Wadhwa | | MD | Foothills Medical Centre, University of Calgary | Calgary, AB, Canada | Collaborator | |
| Martha | Marko | | MD | Foothills Medical Centre, University of Calgary | Calgary, AB, Canada | Collaborator | |
| Nishita | Singh | | MD | Foothills Medical Centre, University of Calgary | Calgary, AB, Canada | Collaborator | |

*Indicates required information. Only first name, last name, and suffix will appear in PubMed.

| ***First Name and Middle Initial(s)** | ***Last Name** | ***Suffix (eg, Jr, III)** | Academic Degrees | Institution | Location (city, state/province, country) | Role or Contribution, eg, chair, principal investigator | Group (if more than 1 Group listed in the byline) and/or Subgroup (eg, Steering Committee) |
|---|---|---|---|---|---|---|---|
| Sanchea | Wasyliw | | MD | Foothills Medical Centre, University of Calgary | Calgary, AB, Canada | Collaborator | |
| Karla J. | Ryckborst | | RN | Foothills Medical Centre, University of Calgary | Calgary, AB, Canada | Collaborator | |
| Carol | Kenney | | RN | Foothills Medical Centre, University of Calgary | Calgary, AB, Canada | Collaborator | |
| Supriya | Save | | | Foothills Medical Centre, University of Calgary | Calgary, AB, Canada | Collaborator | |
| Anitha | Jambula | | | Foothills Medical Centre, University of Calgary | Calgary, AB, Canada | Collaborator | |
| Nancy | Newcommon | | | Foothills Medical Centre, University of Calgary | Calgary, AB, Canada | Collaborator | |
| Gavin | Hull | | | Foothills Medical Centre, University of Calgary | Calgary, AB, Canada | Collaborator | |
| Darcy | Blackstock | | | Foothills Medical Centre, University of Calgary | Calgary, AB, Canada | Collaborator | |
| Sharon | Kiszczak | | | Foothills Medical Centre, University of Calgary | Calgary, AB, Canada | Collaborator | |
| Leslie | Zimmel | | | Foothills Medical Centre, University of Calgary | Calgary, AB, Canada | Collaborator | |
| Michelle | Wright | | | Foothills Medical Centre, University of Calgary | Calgary, AB, Canada | Collaborator | |
| Cari | Jahraus | | | Foothills Medical Centre, University of Calgary | Calgary, AB, Canada | Collaborator | |
| Linda | Andersen | | | Foothills Medical Centre, University of Calgary | Calgary, AB, Canada | Collaborator | |
| Shelly | Bohn | | | Foothills Medical Centre, University of Calgary | Calgary, AB, Canada | Collaborator | |
| Joseph | Paul | | | Foothills Medical Centre, University of Calgary | Calgary, AB, Canada | Collaborator | |
| | | | | | | | |
| Brian H. | Buck | | MD | University of Alberta Hospital | Edmonton, AB, Canada | Site PI | |
| Kenneth S. | Butcher | | MD | University of Alberta Hospital | Edmonton, AB, Canada | Site PI | |
| Ashfaq | Shuaib | | MD | University of Alberta Hospital | Edmonton, AB, Canada | Collaborator | |
| Tom | Jeerakathil | | MD | University of Alberta Hospital | Edmonton, AB, Canada | Collaborator | |
| Glen | Jickling | | MD | University of Alberta Hospital | Edmonton, AB, Canada | Collaborator | |

*Indicates required information. Only first name, last name, and suffix will appear in PubMed.

| *First Name and Middle Initial(s) | *Last Name | *Suffix (eg, Jr, III) | Academic Degrees | Institution | Location (city, state/province, country) | Role or Contribution, eg, chair, principal investigator | Group (if more than 1 Group listed in the byline) and/or Subgroup (eg, Steering Committee) |
|---|---|---|---|---|---|---|---|
| Derek J. | Emery | | MD | University of Alberta Hospital | Edmonton, AB, Canada | Collaborator | |
| Jeremy | Rempel | | MD | University of Alberta Hospital | Edmonton, AB, Canada | Collaborator | |
| Richard J. | Ower | | MD | University of Alberta Hospital | Edmonton, AB, Canada | Collaborator | |
| Robert | Ashforth | | MD | University of Alberta Hospital | Edmonton, AB, Canada | Collaborator | |
| Tom | Yeo | | MD | University of Alberta Hospital | Edmonton, AB, Canada | Collaborator | |
| Trevor B. | Kotylak | | MD | University of Alberta Hospital | Edmonton, AB, Canada | Collaborator | |
| Cian | O;Kelly | | MD | University of Alberta Hospital | Edmonton, AB, Canada | Collaborator | |
| Michael | Chow | | MD | University of Alberta Hospital | Edmonton, AB, Canada | Collaborator | |
| Mizaffar | Siddiqui | | MD | University of Alberta Hospital | Edmonton, AB, Canada | Collaborator | |
| Maher | Saqqur | | MD | University of Alberta Hospital | Edmonton, AB, Canada | Collaborator | |
| Atlantic | D'Souza | | MD | University of Alberta Hospital | Edmonton, AB, Canada | Collaborator | |
| Mar | Lloret | | MD | University of Alberta Hospital | Edmonton, AB, Canada | Collaborator | |
| Asif | Butt | | MD | University of Alberta Hospital | Edmonton, AB, Canada | Collaborator | |
| Ali | Nomani | | MD | University of Alberta Hospital | Edmonton, AB, Canada | Collaborator | |
| Hayrapet | Kalashyan | | MD | University of Alberta Hospital | Edmonton, AB, Canada | Collaborator | |
| Sibi | Thirunavukkarasu | | MD | University of Alberta Hospital | Edmonton, AB, Canada | Collaborator | |
| Juline | Jabs | | RN | University of Alberta Hospital | Edmonton, AB, Canada | Collaborator | |
| Paige | Fairall | | | University of Alberta Hospital | Edmonton, AB, Canada | Collaborator | |
| Lori | Piquette | | RN | University of Alberta Hospital | Edmonton, AB, Canada | Collaborator | |
| | | | | | | | |
| Stephen J. | Phillips | | MBBS FRCPC | Queen Elizabeth II Health Science Centre, Dalhousie University | Halifax, NS, Canada | Site PI | |
| A. Laine | Green | | MD, FRCPC | Queen Elizabeth II Health Science Centre, Dalhousie University | Halifax, NS, Canada | Collaborator | |
| Gordon J. | Gubitz | | MD, FRCPC | Queen Elizabeth II Health Science Centre, Dalhousie University | Halifax, NS, Canada | Collaborator | |
| Jens O. | Heidenreich | | MD | Queen Elizabeth II Health Science Centre, Dalhousie University | Halifax, NS, Canada | Collaborator | |
| Thien | Huynh | | MD, FRCPC | Queen Elizabeth II Health Science Centre, Dalhousie University | Halifax, NS, Canada | Collaborator | |
| Jai J. S. | Shankar | | MD, FRCPC | Queen Elizabeth II Health Science Centre, Dalhousie University | Halifax, NS, Canada | Collaborator | |
| William J. | Maloney | | MD, FRCPC | Queen Elizabeth II Health Science Centre, Dalhousie University | Halifax, NS, Canada | Collaborator | |
| Robert | Vandorpe | | MD, FRCPC | Queen Elizabeth II Health Science Centre, Dalhousie University | Halifax, NS, Canada | Collaborator | |

*Indicates required information. Only first name, last name, and suffix will appear in PubMed.

| *First Name and Middle Initial(s) | *Last Name | *Suffix (eg, Jr, III) | Academic Degrees | Institution | Location (city, state/province, country) | Role or Contribution, eg, chair, principal investigator | Group (if more than 1 Group listed in the byline) and/or Subgroup (eg, Steering Committee) |
|---|---|---|---|---|---|---|---|
| Matthias H. | Schmidt | | MD, FRCPC | Queen Elizabeth II Health Science Centre, Dalhousie University | Halifax, NS, Canada | Collaborator | |
| Gwynedd E. | Pickett | | MD, FRCSC | Queen Elizabeth II Health Science Centre, Dalhousie University | Halifax, NS, Canada | Collaborator | |
| Adrienne | Weeks | | MD, PhD, FRCSC | Queen Elizabeth II Health Science Centre, Dalhousie University | Halifax, NS, Canada | Collaborator | |
| Juditch | Jarrett | | RN, CCRP | Queen Elizabeth II Health Science Centre, Dalhousie University | Halifax, NS, Canada | Collaborator | |
| Debbie | MacDonald | | RN, CCRP | Queen Elizabeth II Health Science Centre, Dalhousie University | Halifax, NS, Canada | Collaborator | |
| Joanna | Arsenault | | CRP | Queen Elizabeth II Health Science Centre, Dalhousie University | Halifax, NS, Canada | Collaborator | |
| Ruth | Kinnear | | RN | Queen Elizabeth II Health Science Centre, Dalhousie University | Halifax, NS, Canada | Collaborator | |
| | | | | | | | |
| Jennifer L. | Mandzia | | MD, PhD | London Health Science Centre, Western Univer | London, ON, Canada | Site PI | |
| Michael | Mayich | | MD | London Health Science Centre, Western Univer | London, ON, Canada | Site PI | |
| Melfort | Boulton | | MD, PhD | London Health Science Centre, Western Univer | London, ON, Canada | Collaborator | |
| Maria Bres | Bullrich | | MD | London Health Science Centre, Western Univer | London, ON, Canada | Collaborator | |
| Sebastian | Fridman | | MD | London Health Science Centre, Western Univer | London, ON, Canada | Collaborator | |
| Ruba | Kiwan | | MD | London Health Science Centre, Western Univer | London, ON, Canada | Collaborator | |
| Donald | Lee | | MD | London Health Science Centre, Western Univer | London, ON, Canada | Collaborator | |
| Stephen | Lownie | | MD | London Health Science Centre, Western Univer | London, ON, Canada | Collaborator | |
| Alexander V. | Khaw | | MD | London Health Science Centre, Western Univer | London, ON, Canada | Collaborator | |
| Sachin K. | Pandey | | MD | London Health Science Centre, Western Univer | London, ON, Canada | Collaborator | |
| Manas | Sharma | | MD | London Health Science Centre, Western Univer | London, ON, Canada | Collaborator | |
| Luciano A. | Sposato | | MD | London Health Science Centre, Western Univer | London, ON, Canada | Collaborator | |
| Kevin | Wade | | MD | London Health Science Centre, Western Univer | London, ON, Canada | Collaborator | |
| Beth | Beauchamp | | RN | London Health Science Centre, Western Univer | London, ON, Canada | Collaborator | |
| Lindsay | Lambourn | | RN | London Health Science Centre, Western Univer | London, ON, Canada | Collaborator | |
| Belinda | Amato-Marziali | | | London Health Science Centre, Western Univer | London, ON, Canada | Collaborator | |
| | | | | | | | |
| Daniel | Roy | | MD | CHUM | Montreal, QC, Canada | Site PI | |
| Nicole | Deneault | | MD | CHUM | Montreal, QC, Canada | Collaborator | |
| Yan | Deschaintre | | MD | CHUM | Montreal, QC, Canada | Collaborator | |
| Laura C. | Gioia | | MD | CHUM | Montreal, QC, Canada | Collaborator | |

Supplemental Online Content: Nonauthor Collaborators

*Indicates required information. Only first name, last name, and suffix will appear in PubMed.

| *First Name and Middle Initial(s) | *Last Name | *Suffix (eg, Jr, III) | Academic Degrees | Institution | Location (city, state/province, country) | Role or Contribution, eg, chair, principal investigator | Group (if more than 1 Group listed in the byline) and/or Subgroup (eg, Steering Committee) |
|---|---|---|---|---|---|---|---|
| Grégory | Jacquin | | MD | CHUM | Montreal, QC, Canada | Collaborator | |
| Céline | Odier | | MD | CHUM | Montreal, QC, Canada | Collaborator | |
| Christian | Stapf | | MD, INR | CHUM | Montreal, QC, Canada | Collaborator | |
| Daniela | Iancu | | MD | CHUM | Montreal, QC, Canada | Collaborator | |
| Jean | Raymond | | MD | CHUM | Montreal, QC, Canada | Collaborator | |
| Alain | Weill | | MD | CHUM | Montreal, QC, Canada | Collaborator | |
| Marlène | Lapierre | | BSc | CHUM | Montreal, QC, Canada | Rearch Nurse Coordinator | |
| Nadia | Jadil | | | CHUM | Montreal, QC, Canada | Research administrative assistant | |
| Judlène | Jolteus | | Bsc | CHUM | Montreal, QC, Canada | Clinical Stroke Nurse | |
| | | | | | | | |
| Jeanne | Teitelbaum | | MD | Montreal Neurological Institute, McGill Univers | Montreal, QC, Canada | Site PI | |
| Mark | Angle | | MD | Montreal Neurological Institute, McGill Univers | Montreal, QC, Canada | Collaborator | |
| Mathew | Hannouche | | MD | Montreal Neurological Institute, McGill Univers | Montreal, QC, Canada | Collaborator | |
| Mohamed | Badawy | | MD | Montreal Neurological Institute, McGill Univers | Montreal, QC, Canada | Collaborator | |
| Justin | Letourneau | | MD | Montreal Neurological Institute, McGill Univers | Montreal, QC, Canada | Collaborator | |
| Maria | Cortes | | MD | Montreal Neurological Institute, McGill Univers | Montreal, QC, Canada | Collaborator | |
| Grant | Linnell | | Md | Montreal Neurological Institute, McGill Univers | Montreal, QC, Canada | Collaborator | |
| Donatella | Tampieri | | MD | Montreal Neurological Institute, McGill Univers | Montreal, QC, Canada | Collaborator | |
| Lucy | Vieira | | MD | Montreal Neurological Institute, McGill Univers | Montreal, QC, Canada | Collaborator | |
| Aimen | Moussaddy | | MD | Montreal Neurological Institute, McGill Univers | Montreal, QC, Canada | Collaborator | |
| Catherine | Legault | | MD | Montreal Neurological Institute, McGill Univers | Montreal, QC, Canada | Collaborator | |
| Liam | Durcan | | MD | Montreal Neurological Institute, McGill Univers | Montreal, QC, Canada | Collaborator | |
| Angela | Moore | | MSc | Montreal Neurological Institute, McGill Univers | Montreal, QC, Canada | Collaborator | |
| Erin | Cole | | MSc | Montreal Neurological Institute, McGill Univers | Montreal, QC, Canada | Collaborator | |
| Claire | Magnussen | | PhD | Montreal Neurological Institute, McGill Univers | Montreal, QC, Canada | Collaborator | |
| Kristiana | Salmon | | EMBA | Montreal Neurological Institute, McGill Univers | Montreal, QC, Canada | Collaborator | |
| Salma | Khalil | | MSc | Montreal Neurological Institute, McGill Univers | Montreal, QC, Canada | Collaborator | |
| Isabelle | Desloges | | MSc | Montreal Neurological Institute, McGill Univers | Montreal, QC, Canada | Collaborator | |
| Lindsay | Waxman | | MSc | Montreal Neurological Institute, McGill Univers | Montreal, QC, Canada | Collaborator | |
| Wynne | Abdon | | BScN | Montreal Neurological Institute, McGill Univers | Montreal, QC, Canada | Collaborator | |
| Sonia | Lai | | BSc | Montreal Neurological Institute, McGill Univers | Montreal, QC, Canada | Collaborator | |
| | | | | | | | |
| Dar | Dowlatshahi | | PhD | Ottawa Hospital, University of Ottawa | Ottawa, ON, Canada | Site PI | |
| Daniela | Iancu | | MSc | Ottawa Hospital, University of Ottawa | Ottawa, ON, Canada | Collaborator | |
| Michel | Shamy | | MD | Ottawa Hospital, University of Ottawa | Ottawa, ON, Canada | Collaborator | |

*Indicates required information. Only first name, last name, and suffix will appear in PubMed.

| ***First Name and Middle Initial(s)** | ***Last Name** | ***Suffix (eg, Jr, III)** | Academic Degrees | Institution | Location (city, state/province, country) | Role or Contribution, eg, chair, principal investigator | Group (if more than 1 Group listed in the byline) and/or Subgroup (eg, Steering Committee) |
|---|---|---|---|---|---|---|---|
| Grant | Stotts | | MD | Ottawa Hospital, University of Ottawa | Ottawa, ON, Canada | Collaborator | |
| Dylan | Blacquiere | | MD | Ottawa Hospital, University of Ottawa | Ottawa, ON, Canada | Collaborator | |
| Howard | Lesiuk | | MD | Ottawa Hospital, University of Ottawa | Ottawa, ON, Canada | Collaborator | |
| Aiman | Quateen | | MD | Ottawa Hospital, University of Ottawa | Ottawa, ON, Canada | Collaborator | |
| Brian | Drake | | MB | Ottawa Hospital, University of Ottawa | Ottawa, ON, Canada | Collaborator | |
| Brian | Dewar | | MLIS | Ottawa Hospital, University of Ottawa | Ottawa, ON, Canada | Collaborator | |
| Zeinab | Daham | | | Ottawa Hospital, University of Ottawa | Ottawa, ON, Canada | Collaborator | |
| | | | | | | | |
| Michael E. | Kelly | | MD, PhD | Royal University Hospital, University of Saskatc | Saskatoon, SK, Canada | Site PI | |
| Gary | Hunter | | MD, FRCPC | Royal University Hospital, University of Saskatc | Saskatoon, SK, Canada | Collaborator | |
| Lissa M. | Peeling | | MD, FRCSC | Royal University Hospital, University of Saskatc | Saskatoon, SK, Canada | Collaborator | |
| Brett R. | Graham | | MD, FRCPC | Royal University Hospital, University of Saskatc | Saskatoon, SK, Canada | Collaborator | |
| Ruth | Whelan | | BSN, MSN | Royal University Hospital, University of Saskatc | Saskatoon, SK, Canada | Collaborator | |
| Aaron J. | Garner | | BSN | Royal University Hospital, University of Saskatc | Saskatoon, SK, Canada | Collaborator | |
| Lilian | Urroz | | MD | Royal University Hospital, University of Saskatc | Saskatoon, SK, Canada | Collaborator | |
| Sharleen | Maley | | BA | Royal University Hospital, University of Saskatc | Saskatoon, SK, Canada | Collaborator | |
| | | | | | | | |
| Frank L. | Silver | | MD | University Health Network, University of Toron | Toronto ON, Canada | Site PI | |
| Leanne | Casaubon | | MD | University Health Network, University of Toron | Toronto ON, Canada | Collaborator | |
| Aleksanda | Pikula | | MD | University Health Network, University of Toron | Toronto ON, Canada | Collaborator | |
| Martin del | Campo | | MD | University Health Network, University of Toron | Toronto ON, Canada | Collaborator | |
| Joanna | Schaafsma | | MD | University Health Network, University of Toron | Toronto ON, Canada | Collaborator | |
| Cheryl | Jaigobin | | MD | University Health Network, University of Toron | Toronto ON, Canada | Collaborator | |
| Timo | Krings | | MD | University Health Network, University of Toron | Toronto ON, Canada | Collaborator | |
| Vitor | Pereira | | MD | University Health Network, University of Toron | Toronto ON, Canada | Collaborator | |
| Ronit | Agid | | MD | University Health Network, University of Toron | Toronto ON, Canada | Collaborator | |
| Richard | Farb | | MD | University Health Network, University of Toron | Toronto ON, Canada | Collaborator | |
| Libby | Kalman | | RN | University Health Network, University of Toron | Toronto ON, Canada | Collaborator | |
| Anne | Cayley | | RN | University Health Network, University of Toron | Toronto ON, Canada | Collaborator | |
| Janice | Williams | | | University Health Network, University of Toron | Toronto ON, Canada | Collaborator | |
| Tim | Stewart | | | University Health Network, University of Toron | Toronto ON, Canada | Collaborator | |
| Karen | Misquitta | | RN | University Health Network, University of Toron | Toronto ON, Canada | Collaborator | |
| | | | | | | | |
| Aditya | Bharatha | | MD | St. Michael’s Hospital, University of Toronto | Toronto, ON, Canada | Site PI | |
| Daniel H. | Selchen | | MD, FRCPC | St. Michael’s Hospital, University of Toronto | Toronto, ON, Canada | Collaborator | |
| Thomas R. | Marotta | | MD, FRCPC | St. Michael’s Hospital, University of Toronto | Toronto, ON, Canada | Collaborator | |

Supplemental Online Content: Nonauthor Collaborators

*Indicates required information. Only first name, last name, and suffix will appear in PubMed.

| *First Name and Middle Initial(s) | *Last Name | *Suffix (eg, Jr, III) | Academic Degrees | Institution | Location (city, state/province, country) | Role or Contribution, eg, chair, principal investigator | Group (if more than 1 Group listed in the byline) and/or Subgroup (eg, Steering Committee) |
|---|---|---|---|---|---|---|---|
| Dipanka | Sarma | | MBBS | St. Michael’s Hospital, University of Toronto | Toronto, ON, Canada | Collaborator | |
| Walter J. | Montanera | | MD, FRCPC | St. Michael’s Hospital, University of Toronto | Toronto, ON, Canada | Collaborator | |
| Julian | Spears | | MD, SM, FRCSC, FACS | St. Michael’s Hospital, University of Toronto | Toronto, ON, Canada | Collaborator | |
| Gustavo | Saposnik | | MD, MSc, FRCPC | St. Michael’s Hospital, University of Toronto | Toronto, ON, Canada | Collaborator | |
| Pawel | Kostyrko | | MD, MSc | St. Michael’s Hospital, University of Toronto | Toronto, ON, Canada | Collaborator | |
| Yangmei | Li | | PhD, CCRC, CCRP | St. Michael’s Hospital, University of Toronto | Toronto, ON, Canada | Collaborator | |
| Carmen | Parra-Farinas | | MD | St. Michael’s Hospital, University of Toronto | Toronto, ON, Canada | Collaborator | |
| Jose Danilo Bengzon | Diestro | | MD | St. Michael’s Hospital, University of Toronto | Toronto, ON, Canada | Collaborator | |
| | | | | | | | |
| Marie-Christine | Camden | | MD | CHU de Québec - Enfant-Jésus Hospital, Laval U | Québec City, QC, Canada | Site PI | |
| Steve | Verreault | | MD, FRCPC | CHU de Québec - Enfant-Jésus Hospital, Laval U | Québec City, QC, Canada | Collaborator | |
| Ariane | Mackey | | MD, FRCPC | CHU de Québec - Enfant-Jésus Hospital, Laval U | Québec City, QC, Canada | Collaborator | |
| Marie-Eve | Audet | | MD, FRCPC | CHU de Québec - Enfant-Jésus Hospital, Laval U | Québec City, QC, Canada | Collaborator | |
| Geneviève | Milot | | MD, FRCPC | CHU de Québec - Enfant-Jésus Hospital, Laval U | Québec City, QC, Canada | Collaborator | |
| Pascale | Lavoie | | MD, FRCPC | CHU de Québec - Enfant-Jésus Hospital, Laval U | Québec City, QC, Canada | Collaborator | |
| Jean-Luc | Gariépy | | MD, FRCPC | CHU de Québec - Enfant-Jésus Hospital, Laval U | Québec City, QC, Canada | Collaborator | |
| Karine | Collard | | | CHU de Québec - Enfant-Jésus Hospital, Laval U | Québec City, QC, Canada | Collaborator | |
| Annette | Haché | | | CHU de Québec - Enfant-Jésus Hospital, Laval U | Québec City, QC, Canada | Collaborator | |
| | | | | | | | |
| Demetrios J. | Sahlas | | MD | McMaster University | Hamilton, ON, Canada | Site PI | |
| Ahmed M. | Alshanbari | | MBBS | McMaster University | Hamilton, ON, Canada | Collaborator | |
| Josephine | Baldwin | | | McMaster University | Hamilton, ON, Canada | Collaborator | |
| Luciana | Catanese | | MD | McMaster University | Hamilton, ON, Canada | Collaborator | |
| Bing Y. | Chen | | MD | McMaster University | Hamilton, ON, Canada | Collaborator | |
| Danielle de | Sa Boasquevisque | | MD | McMaster University | Hamilton, ON, Canada | Collaborator | |
| Aviraj S. | Deshmukh | | MBBS | McMaster University | Hamilton, ON, Canada | Collaborator | |
| Josrdan M. | Knapman | | MSc | McMaster University | Hamilton, ON, Canada | Collaborator | |
| Shana | Lamers | | MSc | McMaster University | Hamilton, ON, Canada | Collaborator | |
| Ramiro A. | Larrazabal | | MD | McMaster University | Hamilton, ON, Canada | Collaborator | |
| Susan V. | MacMillan | | MSc | McMaster University | Hamilton, ON, Canada | Collaborator | |
| Lauren M. | Mai | | MD | McMaster University | Hamilton, ON, Canada | Collaborator | |
| Alicia M. | Mattai | | MD | McMaster University | Hamilton, ON, Canada | Collaborator | |

*Indicates required information. Only first name, last name, and suffix will appear in PubMed.

| *First Name and Middle Initial(s) | *Last Name | *Suffix (eg, Jr, III) | Academic Degrees | Institution | Location (city, state/province, country) | Role or Contribution, eg, chair, principal investigator | Group (if more than 1 Group listed in the byline) and/or Subgroup (eg, Steering Committee) |
|---|---|---|---|---|---|---|---|
| Sumiti | Nayar | | MD | McMaster University | Hamilton, ON, Canada | Collaborator | |
| Kuan H. | Ng | | MBBS | McMaster University | Hamilton, ON, Canada | Collaborator | |
| Wieslaw J. | Oczkowski | | MD | McMaster University | Hamilton, ON, Canada | Collaborator | |
| Kanjana S. | Perera | | MBBS | McMaster University | Hamilton, ON, Canada | Collaborator | |
| Mays A. K. | Shawawrah | | MBBS | McMaster University | Hamilton, ON, Canada | Collaborator | |
| Ashkan | Shoamanesh | | MD | McMaster University | Hamilton, ON, Canada | Collaborator | |
| Gita | Sobhi | | BSc | McMaster University | Hamilton, ON, Canada | Collaborator | |
| Nabeel M. | Syed | | MD | McMaster University | Hamilton, ON, Canada | Collaborator | |
| Noran Y. | Yaher | | MBBS | McMaster University | Hamilton, ON, Canada | Collaborator | |
| Cheyanne | Vandervelde | | BSc | McMaster University | Hamilton, ON, Canada | Collaborator | |
| Di | Wang | | Pharm D | McMaster University | Hamilton, ON, Canada | Collaborator | |
| | | | | | | | |
| Thalia S. | Field | | MD, FRCPC, MHSc | University of British Columbia | Vancouver, BC, Canada | Site PI | |
| Stephen C. | Van Gaal | | MD, FRCPC | University of British Columbia | Vancouver, BC, Canada | Collaborator | |
| Philip A. | Teal | | MD, FRCPC | University of British Columbia | Vancouver, BC, Canada | Collaborator | |
| Oscar R. | Benavente | | MD, FRCPC | University of British Columbia | Vancouver, BC, Canada | Collaborator | |
| Laura K. | Wilson | | MD, FRCPC | University of British Columbia | Vancouver, BC, Canada | Collaborator | |
| Sharanpal K. | Mann | | MD, FRCPC | University of British Columbia | Vancouver, BC, Canada | Collaborator | |
| Samuel | Yip | | PhD, MD, FRCPC | University of British Columbia | Vancouver, BC, Canada | Collaborator | |
| Colleen B. | Murphy | | MD, FRCPC | University of British Columbia | Vancouver, BC, Canada | Collaborator | |
| Manraj K.S. | Heran | | MD, FRCPC | University of British Columbia | Vancouver, BC, Canada | Collaborator | |
| Axel | Rohr | | MD, PhD | University of British Columbia | Vancouver, BC, Canada | Collaborator | |
| Fabio | Settecase | | MD, MSc, FRCPC | University of British Columbia | Vancouver, BC, Canada | Collaborator | |
| Gary J. | Redekop | | MD, MSc, FRCSC | University of British Columbia | Vancouver, BC, Canada | Collaborator | |
| Charles S. | Haw | | MD, MSc, FRCSC | University of British Columbia | Vancouver, BC, Canada | Collaborator | |
| Genoveva | Maclean | | RN | University of British Columbia | Vancouver, BC, Canada | Collaborator | |
| Karina | Murray | | RN | University of British Columbia | Vancouver, BC, Canada | Collaborator | |
| Rosalin | Chiu | | RN | University of British Columbia | Vancouver, BC, Canada | Collaborator | |
| Alana M. | Flexman | | MD, FRCPC | University of British Columbia | Vancouver, BC, Canada | Collaborator | |
| Eilidh | Strecha | | RN | University of British Columbia | Vancouver, BC, Canada | Collaborator | |
| Yolanda | Gayton | | RN | University of British Columbia | Vancouver, BC, Canada | Collaborator | |

*Indicates required information. Only first name, last name, and suffix will appear in PubMed.

| *First Name and Middle Initial(s) | *Last Name | *Suffix (eg, Jr, III) | Academic Degrees | Institution | Location (city, state/province, country) | Role or Contribution, eg, chair, principal investigator | Group (if more than 1 Group listed in the byline) and/or Subgroup (eg, Steering Committee) |
|---|---|---|---|---|---|---|---|
| Judy | Yip | | BSc (Pharm), ACPR | University of British Columbia | Vancouver, BC, Canada | Collaborator | |
| | | | | | | | |
| Richard H. | Swartz | | MD, PhD | Sunnybrook Health Sciences Centre, University | Toronto ON, Canada | Site PI | |
| Mark I. | Boulos | | MD | Sunnybrook Health Sciences Centre, University | Toronto ON, Canada | Collaborator | |
| David J. | Gladstone | | MD, PhD | Sunnybrook Health Sciences Centre, University | Toronto ON, Canada | Collaborator | |
| Amy Y. | Yu | | MD | Sunnybrook Health Sciences Centre, University | Toronto ON, Canada | Collaborator | |
| Julia J. | Hopyan | | MBBS | Sunnybrook Health Sciences Centre, University | Toronto ON, Canada | Collaborator | |
| Houman | Khosravani | | MD, PhD | Sunnybrook Health Sciences Centre, University | Toronto ON, Canada | Collaborator | |
| Herbert A. | Manosalva Alzate | | MD | Sunnybrook Health Sciences Centre, University | Toronto ON, Canada | Collaborator | |
| Alisia | Southwell | | HBSc | Sunnybrook Health Sciences Centre, University | Toronto ON, Canada | Collaborator | |
| Maneesha | Kamra | | MD | Sunnybrook Health Sciences Centre, University | Toronto ON, Canada | Collaborator | |
| Kaitlyn N. | Lopes | | BMSc | Sunnybrook Health Sciences Centre, University | Toronto ON, Canada | Collaborator | |
| Arunima | Kapoor | | MSc | Sunnybrook Health Sciences Centre, University | Toronto ON, Canada | Collaborator | |
| Leodante | da Costa | | MD | Sunnybrook Health Sciences Centre, University | Toronto ON, Canada | Collaborator | |
| Victor X. D. | Yang | | MD, PhD | Sunnybrook Health Sciences Centre, University | Toronto ON, Canada | Collaborator | |
| Erin E. | Dyer | | MD | Sunnybrook Health Sciences Centre, University | Toronto ON, Canada | Collaborator | |
| | | | | | | | |
| Ruchir A. | Shah | | MD | Erlanger Hospital | Chattanooga, TN, US | Site PI | |
| Brenda M. | Knowles | | MSN, RN | Erlanger Hospital | Chattanooga, TN, US | Collaborator | |
| Jennifer | Nichols | | MSN, AGACNP-BC | Erlanger Hospital | Chattanooga, TN, US | Collaborator | |
| Jennifer L. | Patterson | | RN, MSN, ACNP-BC, CCRN, FHM | Erlanger Hospital | Chattanooga, TN, US | Collaborator | |
| Krista A. | Cope | | Pharm D, RD, LDN | Erlanger Hospital | Chattanooga, TN, US | Collaborator | |
| Lee | Dickerson | | aPT | Erlanger Hospital | Chattanooga, TN, US | Collaborator | |
| Katrina K. | Barton | | CCRC | Erlanger Hospital | Chattanooga, TN, US | Collaborator | |
| Kimberly D. | Gray | | CRC | Erlanger Hospital | Chattanooga, TN, US | Collaborator | |
| Ticey N. | Massengale | | CRC | Erlanger Hospital | Chattanooga, TN, US | Collaborator | |
| Melanie G. | Miller | | RN, CRNI | Erlanger Hospital | Chattanooga, TN, US | Collaborator | |
| Cindy | Simpson | | BSN, RN | Erlanger Hospital | Chattanooga, TN, US | Collaborator | |
| Sabrina | Walker | | MSN, RN-BC | Erlanger Hospital | Chattanooga, TN, US | Collaborator | |
| Chlin R. | Tennyson-Yemm | | BSN RN | Erlanger Hospital | Chattanooga, TN, US | Collaborator | |
| | | | | | | | |

*Indicates required information. Only first name, last name, and suffix will appear in PubMed.

| *First Name and Middle Initial(s) | *Last Name | *Suffix (eg, Jr, III) | Academic Degrees | Institution | Location (city, state/province, country) | Role or Contribution, eg, chair, principal investigator | Group (if more than 1 Group listed in the byline) and/or Subgroup (eg, Steering Committee) |
|---|---|---|---|---|---|---|---|
| Donald F. | Frei | | MD | Swedish Medical Center- Colorado Neurologica | Denver, CO, US | Site PI | |
| Richard J, | Bellon | | MD | Swedish Medical Center- Colorado Neurologica | Denver, CO, US | Collaborator | |
| Benjamin N. | Atchie | | DO | Swedish Medical Center- Colorado Neurologica | Denver, CO, US | Collaborator | |
| Ian A. | Kaminsky | | MD | Swedish Medical Center- Colorado Neurologica | Denver, CO, US | Collaborator | |
| Duandelyn C. | Wilson | | NP | Swedish Medical Center- Colorado Neurologica | Denver, CO, US | Collaborator | |
| Nicolle L. | Schraeder | | NP | Swedish Medical Center- Colorado Neurologica | Denver, CO, US | Collaborator | |
| Eric J. | Arias | | MD | Swedish Medical Center- Colorado Neurologica | Denver, CO, US | Collaborator | |
| Lisa M. | Kodis | | MSN, RN | Swedish Medical Center- Colorado Neurologica | Denver, CO, US | Collaborator | |
| Mark E. | Talley | | MS | Swedish Medical Center- Colorado Neurologica | Denver, CO, US | Collaborator | |
| Alex A. | Edinger | | BS | Swedish Medical Center- Colorado Neurologica | Denver, CO, US | Collaborator | |
| Tiffany C. | Talley | | MS | Swedish Medical Center- Colorado Neurologica | Denver, CO, US | Collaborator | |
| Ita P. | Dempsey | | RN | Swedish Medical Center- Colorado Neurologica | Denver, CO, US | Collaborator | |
| Laurie A. | Williams | | RN | Swedish Medical Center- Colorado Neurologica | Denver, CO, US | Collaborator | |
| Scott A. | Williams | | Pharm D, BCOP | Swedish Medical Center- Colorado Neurologica | Denver, CO, US | Collaborator | |
| Sonny | Kupniewski | | Pharm D, BCPS | Swedish Medical Center- Colorado Neurologica | Denver, CO, US | Collaborator | |
| Brad K. | Fasbinder | | BS | Swedish Medical Center- Colorado Neurologica | Denver, CO, US | Collaborator | |
| Joanna M. | Snead | | MS, CCRP | Swedish Medical Center- Colorado Neurologica | Denver, CO, US | Collaborator | |
| | | | | | | | |
| Hana | Choe | | MD | Neurosciences Institute, Abington Jefferson Ho | Philadelphia, PA, US | Site PI | |
| Larami | Mackenzie | | MD | Neurosciences Institute, Abington Jefferson Ho | Philadelphia, PA, US | Collaborator | |
| David C. | Weisman | | MD | Neurosciences Institute, Abington Jefferson Ho | Philadelphia, PA, US | Collaborator | |
| Osman S. | Kozak | | MD | Neurosciences Institute, Abington Jefferson Ho | Philadelphia, PA, US | Collaborator | |
| Qaisar A. | Shah | | MD | Neurosciences Institute, Abington Jefferson Ho | Philadelphia, PA, US | Collaborator | |
| Dan | Gzesh | | MD | Neurosciences Institute, Abington Jefferson Ho | Philadelphia, PA, US | Collaborator | |
| Kandan | Kulandaivel | | MD | Neurosciences Institute, Abington Jefferson Ho | Philadelphia, PA, US | Collaborator | |
| John S. | Khoury | | MD | Neurosciences Institute, Abington Jefferson Ho | Philadelphia, PA, US | Collaborator | |
| Brad | Klein | | MD, MBA | Neurosciences Institute, Abington Jefferson Ho | Philadelphia, PA, US | Collaborator | |
| Patricia A. | Bussinger | | MBA, MSN,AGACNP-BC, CRNP | Neurosciences Institute, Abington Jefferson Ho | Philadelphia, PA, US | Collaborator | |
| Lisa R. | Griffin | | AGACNP-BC, CRNP | Neurosciences Institute, Abington Jefferson Ho | Philadelphia, PA, US | Collaborator | |
| Ashley L. | DePalmo | | BA, CRC | Neurosciences Institute, Abington Jefferson Ho | Philadelphia, PA, US | Collaborator | |
| Cynthia | Oliva | | PharmD | Neurosciences Institute, Abington Jefferson Ho | Philadelphia, PA, US | Collaborator | |

Supplemental Online Content: Nonauthor Collaborators

*Indicates required information. Only first name, last name, and suffix will appear in PubMed.

| *First Name and Middle Initial(s) | *Last Name | *Suffix (eg, Jr, III) | Academic Degrees | Institution | Location (city, state/province, country) | Role or Contribution, eg, chair, principal investigator | Group (if more than 1 Group listed in the byline) and/or Subgroup (eg, Steering Committee) |
|---|---|---|---|---|---|---|---|
| | | | | | | | |
| Ashutosh | Jadhav | | MD | UPMC Medical Centre | Pittsburgh, PA, US | Site PI | |
| Tudor | Jovin | | MD | UPMC Medical Centre | Pittsburgh, PA, US | Collaborator | |
| Cynthia | Kenmuir | | MD | UPMC Medical Centre | Pittsburgh, PA, US | Collaborator | |
| Brian | Jankowitz | | MD | UPMC Medical Centre | Pittsburgh, PA, US | Collaborator | |
| Bradley | Gross | | MD | UPMC Medical Centre | Pittsburgh, PA, US | Collaborator | |
| Marcelo | Rocha | | MD | UPMC Medical Centre | Pittsburgh, PA, US | Collaborator | |
| Matthew | Starr | | MD | UPMC Medical Centre | Pittsburgh, PA, US | Collaborator | |
| Merritt | Brown | | MD | UPMC Medical Centre | Pittsburgh, PA, US | Collaborator | |
| Chrstine | Hawkes | | MD | UPMC Medical Centre | Pittsburgh, PA, US | Collaborator | |
| Kavit | Shah | | MD | UPMC Medical Centre | Pittsburgh, PA, US | Collaborator | |
| Danoushka | Tememe | | MD | UPMC Medical Centre | Pittsburgh, PA, US | Collaborator | |
| Gregory | Walker | | MD | UPMC Medical Centre | Pittsburgh, PA, US | Collaborator | |
| Pratit | Patel | | MD | UPMC Medical Centre | Pittsburgh, PA, US | Collaborator | |
| Bradley | Klein | | MD | UPMC Medical Centre | Pittsburgh, PA, US | Collaborator | |
| Habibullah | Ziayee | | MD | UPMC Medical Centre | Pittsburgh, PA, US | Collaborator | |
| Kaustubh | Limaye | | MD | UPMC Medical Centre | Pittsburgh, PA, US | Collaborator | |
| Lisa | Baxendell | | BSN | UPMC Medical Centre | Pittsburgh, PA, US | Collaborator | |
| Vicki | Glichrist | | BSN | UPMC Medical Centre | Pittsburgh, PA, US | Collaborator | |
| Patricia | Feineigle | | BSN,PhD | UPMC Medical Centre | Pittsburgh, PA, US | Collaborator | |
| Kelsea | Toseki Haibach | | BSN | UPMC Medical Centre | Pittsburgh, PA, US | Collaborator | |
| Cathy | Van Every | | BSN | UPMC Medical Centre | Pittsburgh, PA, US | Collaborator | |
| Shasvat | Desai | | | UPMC Medical Centre | Pittsburgh, PA, US | Collaborator | |
| Maryam | Zulfiqar | | | UPMC Medical Centre | Pittsburgh, PA, US | Collaborator | |
| Linda | Gibson | | | UPMC Medical Centre | Pittsburgh, PA, US | Collaborator | |
| Sean | Barrett | | | UPMC Medical Centre | Pittsburgh, PA, US | Collaborator | |
| | | | | | | | |
| David | Turkel-Parrella | | MD | NYU School of Medicine | Brooklyn, NY, US | Site PI | |
| Karthikeyan | Arcot | | MD | NYU School of Medicine | Brooklyn, NY, US | Collaborator | |
| Steven | DiCrescento | | RPh | NYU School of Medicine | Brooklyn, NY, US | Collaborator | |
| Jeffrey | Farkas | | MD | NYU School of Medicine | Brooklyn, NY, US | Collaborator | |
| Gregory | Filipowski | | RPh | NYU School of Medicine | Brooklyn, NY, US | Collaborator | |
| Jennifer | Frontera | | MD | NYU School of Medicine | Brooklyn, NY, US | Collaborator | |
| Danielle | Joset | | PharmD | NYU School of Medicine | Brooklyn, NY, US | Collaborator | |
| Jeremy | Liff | | MD | NYU School of Medicine | Brooklyn, NY, US | Collaborator | |
| Erica | Scher | | RN, MPH | NYU School of Medicine | Brooklyn, NY, US | Collaborator | |

*Indicates required information. Only first name, last name, and suffix will appear in PubMed.

| *First Name and Middle Initial(s) | *Last Name | *Suffix (eg, Jr, III) | Academic Degrees | Institution | Location (city, state/province, country) | Role or Contribution, eg, chair, principal investigator | Group (if more than 1 Group listed in the byline) and/or Subgroup (eg, Steering Committee) |
|---|---|---|---|---|---|---|---|
| | | | | | | | |
| Cameron G. | McDougall | | MD | Swedish Neurological Institute | Seattle, WA, US | Site PI | |
| Cheryl E. | Kelly | | BSN, CNRN | Swedish Neurological Institute | Seattle, WA, US | Collaborator | |
| Akshal S. | Patel | | MD | Swedish Neurological Institute | Seattle, WA, US | Collaborator | |
| Stephen J. | Monteith | | MD | Swedish Neurological Institute | Seattle, WA, US | Collaborator | |
| | | | | | | | |
| Sidney | Starkman | | MD | UCLA Comprehensive Stroke Center, University | Los Angeles, Los Angeles, | Site PI | |
| Kunakorn C. | Atchaneeyasakul | | MD | UCLA Comprehensive Stroke Center, University | Los Angeles, Los Angeles, | Collaborator | |
| Adrian M. | Burgos | | MD | UCLA Comprehensive Stroke Center, University | Los Angeles, Los Angeles, | Collaborator | |
| Janice | Anne Y. Chua | | BSN, CNRN | UCLA Comprehensive Stroke Center, University | Los Angeles, Los Angeles, | Collaborator | |
| Nathan D. | Gaines | | MD | UCLA Comprehensive Stroke Center, University | Los Angeles, Los Angeles, | Collaborator | |
| Ileana D. | Grunberg | | RN | UCLA Comprehensive Stroke Center, University | Los Angeles, Los Angeles, | Collaborator | |
| Judy | Guzy | | RN | UCLA Comprehensive Stroke Center, University | Los Angeles, Los Angeles, | Collaborator | |
| Zuolu | Liu | | MD | UCLA Comprehensive Stroke Center, University | Los Angeles, Los Angeles, | Collaborator | |
| Neil D. | Maluste | | MD | UCLA Comprehensive Stroke Center, University | Los Angeles, Los Angeles, | Collaborator | |
| Lucas | Ramirez | | MD | UCLA Comprehensive Stroke Center, University | Los Angeles, Los Angeles, | Collaborator | |
| Latisha K. | Sharma | | MD | UCLA Comprehensive Stroke Center, University | Los Angeles, Los Angeles, | Collaborator | |
| | | | | | | | |
| Donald | Heck | | MD | Forsyth Medical Center | Winston-Salem, NC, US | Site PI | |
| Morry | Brown | | MD, PhD | Forsyth Medical Center | Winston-Salem, NC, US | Collaborator | |
| Colin | McDonald | | MD | Forsyth Medical Center | Winston-Salem, NC, US | Collaborator | |
| Mateo | Calderon | | MD | Forsyth Medical Center | Winston-Salem, NC, US | Collaborator | |
| Mitch | Hargis | | MD | Forsyth Medical Center | Winston-Salem, NC, US | Collaborator | |
| Christina | Roels | | Pharm D | Forsyth Medical Center | Winston-Salem, NC, US | Collaborator | |
| Prabhu | Emmady | | MD | Forsyth Medical Center | Winston-Salem, NC, US | Collaborator | |
| Talat | Alvi | | MD | Forsyth Medical Center | Winston-Salem, NC, US | Collaborator | |
| | | | | | | | |
| Mahesh V. | Jayaraman | | MD | Warren Alpert School of Medicine at Brown Ur | Providence, RI, US | Collaborator | |
| Shawna M. | Cutting | | MD | Warren Alpert School of Medicine at Brown Ur | Providence, RI, US | Collaborator | |
| Wendy J. | Smith | | BS, RTRCV | Warren Alpert School of Medicine at Brown Ur | Providence, RI, US | Collaborator | |
| Susan M. | Foley | | RTRCV | Warren Alpert School of Medicine at Brown Ur | Providence, RI, US | Collaborator | |
| Gino A. | Paolucci | | NP | Warren Alpert School of Medicine at Brown Ur | Providence, RI, US | Collaborator | |
| Richard A. | Haas | | MD | Warren Alpert School of Medicine at Brown Ur | Providence, RI, US | Collaborator | |
| Katie A. | Quinn | | PA | Warren Alpert School of Medicine at Brown Ur | Providence, RI, US | Collaborator | |
| Lindsey R. | Fuller | | PA | Warren Alpert School of Medicine at Brown Ur | Providence, RI, US | Collaborator | |
| Rebecca E. | Brierley | | PA | Warren Alpert School of Medicine at Brown Ur | Providence, RI, US | Collaborator | |

Supplemental Online Content: Nonauthor Collaborators
*Indicates required information. Only first name, last name, and suffix will appear in PubMed.

| *First Name and Middle Initial(s) | *Last Name | *Suffix (eg, Jr, III) | Academic Degrees | Institution | Location (city, state/province, country) | Role or Contribution, eg, chair, principal investigator | Group (if more than 1 Group listed in the byline) and/or Subgroup (eg, Steering Committee) |
|---|---|---|---|---|---|---|---|
| Christina C. | Watkins | | NP | Warren Alpert School of Medicine at Brown Ur | Providence, RI, US | Collaborator | |
| Nicole C. | Demir | | NP | Warren Alpert School of Medicine at Brown Ur | Providence, RI, US | Collaborator | |
| | | | | | | | |
| George A. | Lopez | | MD, PhD | Swedish Neurological Institute | Seattle, WA, US | Site PI | |
| Alejandro | Vargas | | MD | Rush University Medical Center | Chicago, IL, US | Collaborator | |
| Nicholas | Osteraas | | MD | Rush University Medical Center | Chicago, IL, US | Collaborator | |
| Becky | Holtz | | RN | Rush University Medical Center | Chicago, IL, US | Collaborator | |
| | | | | | | | |
| Eric | Sauvageau | | MD | Lyerly Neurosurgery, Baptist Hospital | Jacksonville, FL, US | Site PI | |
| Amin | Aghaebrahim | | MD | Lyerly Neurosurgery, Baptist Hospital | Jacksonville, FL, US | Collaborator | |
| Mohamad | Chmayssani | | MD | Lyerly Neurosurgery, Baptist Hospital | Jacksonville, FL, US | Collaborator | |
| Neeraj S. | Naval | | MD | Lyerly Neurosurgery, Baptist Hospital | Jacksonville, FL, US | Collaborator | |
| Jason S. | Day | | MD | Lyerly Neurosurgery, Baptist Hospital | Jacksonville, FL, US | Collaborator | |
| Scott M. | Dellorso | | MD | Lyerly Neurosurgery, Baptist Hospital | Jacksonville, FL, US | Collaborator | |
| Benjamin J. | Ludwig | | MD | Lyerly Neurosurgery, Baptist Hospital | Jacksonville, FL, US | Collaborator | |
| Derek J. | Schemmel | | MD | Lyerly Neurosurgery, Baptist Hospital | Jacksonville, FL, US | Collaborator | |
| Nancy | Ebreo | | CCRC, RRT | Lyerly Neurosurgery, Baptist Hospital | Jacksonville, FL, US | Collaborator | |
| Karen | Bell | | BSH | Lyerly Neurosurgery, Baptist Hospital | Jacksonville, FL, US | Collaborator | |
| Lanai | Lewis | | CRC | Lyerly Neurosurgery, Baptist Hospital | Jacksonville, FL, US | Collaborator | |
| Marjorie | Delucia | | PharmD | Lyerly Neurosurgery, Baptist Hospital | Jacksonville, FL, US | Collaborator | |
| | | | | | | | |
| Diogo | Haussen | | MD | Emory University School of Medicine, Grady M | Atlanta, GA, US | Collaborator | |
| Michael | Frankel | | MD | Emory University School of Medicine, Grady M | Atlanta, GA, US | Collaborator | |
| Nirav | Bhatt | | MD | Emory University School of Medicine, Grady M | Atlanta, GA, US | Collaborator | |
| Nicolas | Bianchi | | MD | Emory University School of Medicine, Grady M | Atlanta, GA, US | Collaborator | |
| Aaron | Anderson | | MD | Emory University School of Medicine, Grady M | Atlanta, GA, US | Collaborator | |
| Samir | Belagaje | | MD | Emory University School of Medicine, Grady M | Atlanta, GA, US | Collaborator | |
| Bernardo | Liberato | | MD | Emory University School of Medicine, Grady M | Atlanta, GA, US | Collaborator | |
| Srikant | Rangaraju | | MD | Emory University School of Medicine, Grady M | Atlanta, GA, US | Collaborator | |
| Alhamza R. | Al-Bayati | | MD | Emory University School of Medicine, Grady M | Atlanta, GA, US | Collaborator | |
| Jonathan | Grossber | | MD | Emory University School of Medicine, Grady M | Atlanta, GA, US | Collaborator | |
| Leah P. | Craft | | RN, BSN, CCRC | Emory University School of Medicine, Grady M | Atlanta, GA, US | Collaborator | |
| Kiva | Schindler | | RN, CCRC | Emory University School of Medicine, Grady M | Atlanta, GA, US | Collaborator | |
| Erin | Schaad | | CRC | Emory University School of Medicine, Grady M | Atlanta, GA, US | Collaborator | |
| Meagan | Schultz | | CRC | Emory University School of Medicine, Grady M | Atlanta, GA, US | Collaborator | |

*Indicates required information. Only first name, last name, and suffix will appear in PubMed.

| *First Name and Middle Initial(s) | *Last Name | *Suffix (eg, Jr, III) | Academic Degrees | Institution | Location (city, state/province, country) | Role or Contribution, eg, chair, principal investigator | Group (if more than 1 Group listed in the byline) and/or Subgroup (eg, Steering Committee) |
|---|---|---|---|---|---|---|---|
| Lorretta J. | Sutherly | | MS, BSN, RN-BC, SCRN, NVRN, CCRC | Emory University School of Medicine, Grady M | Atlanta, GA, US | Collaborator | |
| Shannon | Doppelheuer | | CCRC | Emory University School of Medicine, Grady M | Atlanta, GA, US | Collaborator | |
| Jacquelyn | Charlton | | CRC | Emory University School of Medicine, Grady M | Atlanta, GA, US | Collaborator | |
| Jason | Faggard | | RN | Emory University School of Medicine, Grady M | Atlanta, GA, US | Collaborator | |
| Zuzana | Barbret | | RN | Emory University School of Medicine, Grady M | Atlanta, GA, US | Collaborator | |
| Ethan | DukSoo Han | | RN | Emory University School of Medicine, Grady M | Atlanta, GA, US | Collaborator | |
| Robin | Walters | | RN | Emory University School of Medicine, Grady M | Atlanta, GA, US | Collaborator | |
| Jaydevsinh | Dolia | | MD | Emory University School of Medicine, Grady M | Atlanta, GA, US | Collaborator | |
| Sleiman | El-Jamal | | MD | Emory University School of Medicine, Grady M | Atlanta, GA, US | Collaborator | |
| Brendan | Eby | | MD | Emory University School of Medicine, Grady M | Atlanta, GA, US | Collaborator | |
| Yasir | Saleem | | MD | Emory University School of Medicine, Grady M | Atlanta, GA, US | Collaborator | |
| Harrison | Pearl | | MD | Emory University School of Medicine, Grady M | Atlanta, GA, US | Collaborator | |
| Kishan | Patel | | MD | Emory University School of Medicine, Grady M | Atlanta, GA, US | Collaborator | |
| Haseeb | Rahman | | MD | Emory University School of Medicine, Grady M | Atlanta, GA, US | Collaborator | |
| Reema | Butt | | MD | Emory University School of Medicine, Grady M | Atlanta, GA, US | Collaborator | |
| Stephen | English | | MD | Emory University School of Medicine, Grady M | Atlanta, GA, US | Collaborator | |
| | | | | | | | |
| Ajit S. | Puri | | MD | University of Massachusetts Medical Center | Worcester, MA, US | Site PI | |
| Mary | Howk | | MS | University of Massachusetts Medical Center | Worcester, MA, US | Collaborator | |
| Jasmeet | Singh | | MD | University of Massachusetts Medical Center | Worcester, MA, US | Collaborator | |
| Francesco | Massari | | MD, PhD | University of Massachusetts Medical Center | Worcester, MA, US | Collaborator | |
| Katyucia | DeMacedo Rodrigues | | MD | University of Massachusetts Medical Center | Worcester, MA, US | Collaborator | |
| Anna L. | Kuhn | | MD, PhD | University of Massachusetts Medical Center | Worcester, MA, US | Collaborator | |
| | | | | | | | |
| Joey D. | English | | MD | California Pacific Medical Center, Sutter Health | San Francisco, CA, US | Site PI | |
| Nobl | Barazangi | | MD | California Pacific Medical Center, Sutter Health | San Francisco, CA, US | Collaborator | |
| Nick | Telischak | | MD | California Pacific Medical Center, Sutter Health | San Francisco, CA, US | Collaborator | |
| Warren | Kim | | MD | California Pacific Medical Center, Sutter Health | San Francisco, CA, US | Collaborator | |
| Josh | Ross | | RN | California Pacific Medical Center, Sutter Health | San Francisco, CA, US | Collaborator | |
| Nata | DeVole | | RN | California Pacific Medical Center, Sutter Health | San Francisco, CA, US | Collaborator | |
| Jessica | Redford | | RN | California Pacific Medical Center, Sutter Health | San Francisco, CA, US | Collaborator | |
| Melina | Ferreira | | | California Pacific Medical Center, Sutter Health | San Francisco, CA, US | Collaborator | |
| Katie | Ponting | | | California Pacific Medical Center, Sutter Health | San Francisco, CA, US | Collaborator | |

*Indicates required information. Only first name, last name, and suffix will appear in PubMed.

| *First Name and Middle Initial(s) | *Last Name | *Suffix (eg, Jr, III) | Academic Degrees | Institution | Location (city, state/province, country) | Role or Contribution, eg, chair, principal investigator | Group (if more than 1 Group listed in the byline) and/or Subgroup (eg, Steering Committee) |
|---|---|---|---|---|---|---|---|
| Helen | Shen | | | California Pacific Medical Center, Sutter Health | San Francisco, CA, US | Collaborator | |
| Ann | Bedenk | | RN | California Pacific Medical Center, Sutter Health | San Francisco, CA, US | Collaborator | |
| Susila | Patel | | RN | California Pacific Medical Center, Sutter Health | San Francisco, CA, US | Collaborator | |
| Julia | Fernandess | | RN | California Pacific Medical Center, Sutter Health | San Francisco, CA, US | Collaborator | |
| Michael | Ke | | MD | California Pacific Medical Center, Sutter Health | San Francisco, CA, US | Collaborator | |
| Illanit | Spokoyny | | MD | California Pacific Medical Center, Sutter Health | San Francisco, CA, US | Collaborator | |
| Billy | Gao | | MD | California Pacific Medical Center, Sutter Health | San Francisco, CA, US | Collaborator | |
| David | Tong | | MD | California Pacific Medical Center, Sutter Health | San Francisco, CA, US | Collaborator | |
| Charlene | Chen | | MD | California Pacific Medical Center, Sutter Health | San Francisco, CA, US | Collaborator | |
| Christine | Wong | | MD | California Pacific Medical Center, Sutter Health | San Francisco, CA, US | Collaborator | |
| Jessica | Choe | | MD | California Pacific Medical Center, Sutter Health | San Francisco, CA, US | Collaborator | |
| | | | | | | | |
| Coleman O | Martin | | MD | Saint Luke’s Hospital of Kansas City | Kansas City, MO, US | Site PI | |
| Debbie V. | Summers | | MSN | Saint Luke’s Hospital of Kansas City | Kansas City, MO, US | Collaborator | |
| Christine M. | Boutwell | | MD | Saint Luke’s Hospital of Kansas City | Kansas City, MO, US | Collaborator | |
| Karin E. | Olds | | MD | Saint Luke’s Hospital of Kansas City | Kansas City, MO, US | Collaborator | |
| Suzanne C. | Crandall | | MD | Saint Luke’s Hospital of Kansas City | Kansas City, MO, US | Collaborator | |
| John D. | Eatman | | MD | Saint Luke’s Hospital of Kansas City | Kansas City, MO, US | Collaborator | |
| Naveed | Akhtar | | MD | Saint Luke’s Hospital of Kansas City | Kansas City, MO, US | Collaborator | |
| William | Holloway | | MD | Saint Luke’s Hospital of Kansas City | Kansas City, MO, US | Collaborator | |
| Jared S. | Halpin | | MD | Saint Luke’s Hospital of Kansas City | Kansas City, MO, US | Collaborator | |
| Brett | Donegan | | MD | Saint Luke’s Hospital of Kansas City | Kansas City, MO, US | Collaborator | |
| | | | | | | | |
| Joseph L. | Schindler | | MD | Yale University School of Medicine | New Haven, CT, US | Site PI | |
| Daiv Y. | Hwang | | MD | Yale University School of Medicine | New Haven, CT, US | Collaborator | |
| Emily J. | Gilmore | | MD | Yale University School of Medicine | New Haven, CT, US | Collaborator | |
| Nils | Petersen | | MD | Yale University School of Medicine | New Haven, CT, US | Collaborator | |
| Kevin N. | Sheth | | MD | Yale University School of Medicine | New Haven, CT, US | Collaborator | |
| Stacy Y. | Brown | | MD | Yale University School of Medicine | New Haven, CT, US | Collaborator | |
| Rachel B. | Beekman | | MD | Yale University School of Medicine | New Haven, CT, US | Collaborator | |
| Benjamin P. | George | | MD | Yale University School of Medicine | New Haven, CT, US | Collaborator | |
| Firas | Kaddouh | | MD | Yale University School of Medicine | New Haven, CT, US | Collaborator | |
| Guido J. | Falcone | | MD | Yale University School of Medicine | New Haven, CT, US | Collaborator | |
| Charles R. | Wira | | MD | Yale University School of Medicine | New Haven, CT, US | Collaborator | |
| Charles C. | Matouk | | MD | Yale University School of Medicine | New Haven, CT, US | Collaborator | |
| Caitlin | Loomis | | MD | Yale University School of Medicine | New Haven, CT, US | Collaborator | |

Supplemental Online Content: Nonauthor Collaborators

*Indicates required information. Only first name, last name, and suffix will appear in PubMed.

| *First Name and Middle Initial(s) | *Last Name | *Suffix (eg, Jr, III) | Academic Degrees | Institution | Location (city, state/province, country) | Role or Contribution, eg, chair, principal investigator | Group (if more than 1 Group listed in the byline) and/or Subgroup (eg, Steering Committee) |
|---|---|---|---|---|---|---|---|
| Hardik | Amin | | MD | Yale University School of Medicine | New Haven, CT, US | Collaborator | |
| Hans Christoph | Stretz | | MD | Yale University School of Medicine | New Haven, CT, US | Collaborator | |
| Reshma | Narula | | MD | Yale University School of Medicine | New Haven, CT, US | Collaborator | |
| Adam S. | Jasne | | MD | Yale University School of Medicine | New Haven, CT, US | Collaborator | |
| Lauren H. | Sansing | | MD | Yale University School of Medicine | New Haven, CT, US | Collaborator | |
| Tijil | Agarwal | | MD | Yale University School of Medicine | New Haven, CT, US | Collaborator | |
| Sara D. | Jasak | | BSN | Yale University School of Medicine | New Haven, CT, US | Collaborator | |
| Briana D. | Fontaine | | BA | Yale University School of Medicine | New Haven, CT, US | Collaborator | |
| Zachary | King | | BA | Yale University School of Medicine | New Haven, CT, US | Collaborator | |
| Lindsey R. | Kouhn | | BA | Yale University School of Medicine | New Haven, CT, US | Collaborator | |
| Hailey | Orgass | | BA | Yale University School of Medicine | New Haven, CT, US | Collaborator | |
| Megan A. | Leary | | BS | Yale University School of Medicine | New Haven, CT, US | Collaborator | |
| Joan L. | Nye | | BS | Yale University School of Medicine | New Haven, CT, US | Collaborator | |
| Kelsey | Halbert | | RN, BSN, MSN, CNL, SCRN, CNRN | Yale University School of Medicine | New Haven, CT, US | Collaborator | |
| Karin V. | Nystrom | | MSN, APRN, FAHA | Yale University School of Medicine | New Haven, CT, US | Collaborator | |
| Kaile B. | Neuschatz | | BSN, SCRN | Yale University School of Medicine | New Haven, CT, US | Collaborator | |
| Dawn M. | Petrucci | | BSN, SCRN | Yale University School of Medicine | New Haven, CT, US | Collaborator | |
| Anna M. | Coppola | | BS, RN | Yale University School of Medicine | New Haven, CT, US | Collaborator | |
| | | | | | | | |
| Treasure A. | Joyce | | LVN | Providence Little Company of Mary Medical Ce | Torrance, CA, US | Collaborator | |
| Sam Y. | Hou | | MD, PhD | Providence Little Company of Mary Medical Ce | Torrance, CA, US | Collaborator | |
| Mark S. | Umekubo | | Pharm D, BCCP | Providence Little Company of Mary Medical Ce | Torrance, CA, US | Collaborator | |
| Catrice M. | Nakamura | | MSN, SCRN | Providence Little Company of Mary Medical Ce | Torrance, CA, US | Collaborator | |
| Renee M. | Ovando | | NP, SCRN | Providence Little Company of Mary Medical Ce | Torrance, CA, US | Collaborator | |
| Diana | Zuniga | | LVN | Providence Little Company of Mary Medical Ce | Torrance, CA, US | Collaborator | |
| Yih Lin | Nien | | MD | Providence Little Company of Mary Medical Ce | Torrance, CA, US | Collaborator | |
| Fernando Mayor | Basto | | MD | Providence Little Company of Mary Medical Ce | Torrance, CA, US | Collaborator | |
| Allison E. | Arch | | MD | Providence Little Company of Mary Medical Ce | Torrance, CA, US | Collaborator | |
| Laura T. | Jong | | MD | Providence Little Company of Mary Medical Ce | Torrance, CA, US | Collaborator | |
| William A. | Conrad | | MD | Providence Little Company of Mary Medical Ce | Torrance, CA, US | Collaborator | |
| Tara C. | Abbott | | BSN | Providence Little Company of Mary Medical Ce | Torrance, CA, US | Collaborator | |
| Jay Z. | Yao | | MD | Providence Little Company of Mary Medical Ce | Torrance, CA, US | Collaborator | |

*Indicates required information. Only first name, last name, and suffix will appear in PubMed.

| *First Name and Middle Initial(s) | *Last Name | *Suffix (eg, Jr, III) | Academic Degrees | Institution | Location (city, state/province, country) | Role or Contribution, eg, chair, principal investigator | Group (if more than 1 Group listed in the byline) and/or Subgroup (eg, Steering Committee) |
|---|---|---|---|---|---|---|---|
| Scott D. | Caganap | | MD | Providence Little Company of Mary Medical Ce | Torrance, CA, US | Collaborator | |
| John E. | Jordan | | MD | Providence Little Company of Mary Medical Ce | Torrance, CA, US | Collaborator | |
| George P. | Teitelbaum | | MD | Providence Little Company of Mary Medical Ce | Torrance, CA, US | Collaborator | |
| Robert J. | Darflinger | | MD | Providence Little Company of Mary Medical Ce | Torrance, CA, US | Collaborator | |
| Daniel F. | Kelly | | MD | Providence Little Company of Mary Medical Ce | Torrance, CA, US | Collaborator | |
| | | | | | | | |
| Rishi | Gupta | | MD | Wellstar Health System, Kennestone Hospital | Marietta, GA, US | Site PI | |
| | | | | | | | |
| Ronald F. | Budzik | | MD | Ohio Health, Riverside Methodist Hospital | Columbus, OH, US | Site PI | |
| Jennifer H. | Czerniak | | RN | Ohio Health, Riverside Methodist Hospital | Columbus, OH, US | Collaborator | |
| Katherine M. | Groezinger | | BSN, RN, SCRN | Ohio Health, Riverside Methodist Hospital | Columbus, OH, US | Collaborator | |
| William J. | Hicks | II | MD | Ohio Health, Riverside Methodist Hospital | Columbus, OH, US | Collaborator | |
| Omran B. | Kaskar | | DO | Ohio Health, Riverside Methodist Hospital | Columbus, OH, US | Collaborator | |
| Brian S. | Katz | | MD | Ohio Health, Riverside Methodist Hospital | Columbus, OH, US | Collaborator | |
| Aaron I. | Loochtan | | DO | Ohio Health, Riverside Methodist Hospital | Columbus, OH, US | Collaborator | |
| Peter J. | Pema | | MD | Ohio Health, Riverside Methodist Hospital | Columbus, OH, US | Collaborator | |
| Vivek | Rai | | MD | Ohio Health, Riverside Methodist Hospital | Columbus, OH, US | Collaborator | |
| Nirav A. | Vora | | MD | Ohio Health, Riverside Methodist Hospital | Columbus, OH, US | Collaborator | |
| Amanda | Brown | | MPH | Ohio Health, Riverside Methodist Hospital | Columbus, OH, US | Collaborator | |
| Diane | Goodman | | | Ohio Health, Riverside Methodist Hospital | Columbus, OH, US | Collaborator | |
| Barb | Danenbergs | | RPh, CCRP | Ohio Health, Riverside Methodist Hospital | Columbus, OH, US | Collaborator | |
| Monica | Gossett | | Pharm D | Ohio Health, Riverside Methodist Hospital | Columbus, OH, US | Collaborator | |
| | | | | | | | |
| Oh Young | Bang | | MD | Samsung Medical Center, Departments of Neu | Seoul, South Korea | Site PI | |
| Jong-Won | Chung | | MD, PhD | Samsung Medical Center, Departments of Neu | Seoul, South Korea | Collaborator | |
| Pyoung | Jeon | | MD, PhD | Samsung Medical Center, Departments of Neu | Seoul, South Korea | Collaborator | |
| Keon Ha | Kim | | MD, PhD | Samsung Medical Center, Departments of Neu | Seoul, South Korea | Collaborator | |
| Yun Jeong | Lim | | RN | Samsung Medical Center, Departments of Neu | Seoul, South Korea | Collaborator | |
| | | | | | | | |
| Ji | Hoe Heo | | MD | Yonsei University College of Medicine | Seoul, South Korea | Site PI | |
| Hyo Suk | Nam | | MD, PhD | Yonsei University College of Medicine | Seoul, South Korea | Collaborator | |
| Young Dae | Kim | | MD, PhD | Yonsei University College of Medicine | Seoul, South Korea | Collaborator | |
| HyungJong | Park | | MD | Yonsei University College of Medicine | Seoul, South Korea | Collaborator | |
| In Gun | Hwang | | MD | Yonsei University College of Medicine | Seoul, South Korea | Collaborator | |

*Indicates required information. Only first name, last name, and suffix will appear in PubMed.

| *First Name and Middle Initial(s) | *Last Name | *Suffix (eg, Jr, III) | Academic Degrees | Institution | Location (city, state/province, country) | Role or Contribution, eg, chair, principal investigator | Group (if more than 1 Group listed in the byline) and/or Subgroup (eg, Steering Committee) |
|---|---|---|---|---|---|---|---|
| Wooseok | Ha | | MD | Yonsei University College of Medicine | Seoul, South Korea | Collaborator | |
| Jin Kyo | Choi | | MD | Yonsei University College of Medicine | Seoul, South Korea | Collaborator | |
| Kyungsun | Jeong | | RN | Yonsei University College of Medicine | Seoul, South Korea | Collaborator | |
| | | | | | | | |
| Joung-Ho | Rha | | MD | Inha University Hospital Neurology | Incheon, South Korea | Site PI | |
| Hee-Kwon | Park | | MD | Inha University Hospital Neurology | Incheon, South Korea | Collaborator | |
| Cindy W. | Yoon | | MD | Inha University Hospital Neurology | Incheon, South Korea | Collaborator | |
| Bo-Kyung | Kim | | RN | Inha University Hospital Neurology | Incheon, South Korea | Collaborator | |
| | | | | | | | |
| Sung-Il | Sohn | | MD | Dongsan Hospital, Keimyung University School | Daegu, South Korea | Site PI | |
| Chang-Hyun | Kim | | MD | Dongsan Hospital, Keimyung University School | Daegu, South Korea | Site PI | |
| Joonsang | Yoo | | MD | Dongsan Hospital, Keimyung University School | Daegu, South Korea | Collaborator | |
| Jeong-Ho | Hong | | MD, PhD | Dongsan Hospital, Keimyung University School | Daegu, South Korea | Collaborator | |
| Hyungjong | Park | | MD | Dongsan Hospital, Keimyung University School | Daegu, South Korea | Collaborator | |
| Sohyeon | Kim | | MD | Dongsan Hospital, Keimyung University School | Daegu, South Korea | Collaborator | |
| Moonkyung | Choi | | MD | Dongsan Hospital, Keimyung University School | Daegu, South Korea | Collaborator | |
| Hyoeun | Bae | | MD | Dongsan Hospital, Keimyung University School | Daegu, South Korea | Collaborator | |
| Jun Seok | Lee | | MD | Dongsan Hospital, Keimyung University School | Daegu, South Korea | Collaborator | |
| Jae-Joon | Lee | | MD | Dongsan Hospital, Keimyung University School | Daegu, South Korea | Collaborator | |
| Go-Eun | Jun | | BA (CRA) | Dongsan Hospital, Keimyung University School | Daegu, South Korea | Collaborator | |
| Sujeong | Jeon | | BA (CRA) | Dongsan Hospital, Keimyung University School | Daegu, South Korea | Collaborator | |
| | | | | | | | |
| Bruce C. V. | Campbell | | PhD | The Royal Melbourne Hospital, University of M | Parkville, Victoria, Austral | Site PI | |
| Peter J. | Mitchell | | Mmed | The Royal Melbourne Hospital, University of M | Parkville, Victoria, Austral | Collaborator | |
| Nawaf | Yassi | | PhD | The Royal Melbourne Hospital, University of M | Parkville, Victoria, Austral | Collaborator | |
| Stephen M. | Davis | | MD | The Royal Melbourne Hospital, University of M | Parkville, Victoria, Austral | Collaborator | |
| Geoffrey A. | Donnan | | MD | The Royal Melbourne Hospital, University of M | Parkville, Victoria, Austral | Collaborator | |
| Mark W. | Parsons | | PhD | The Royal Melbourne Hospital, University of M | Parkville, Victoria, Austral | Collaborator | |
| Bernard | Yan | | DMedSc | The Royal Melbourne Hospital, University of M | Parkville, Victoria, Austral | Collaborator | |
| Richard J. | Dowling | | MBBS | The Royal Melbourne Hospital, University of M | Parkville, Victoria, Austral | Collaborator | |
| Steven J. | Bush | | MBBS | The Royal Melbourne Hospital, University of M | Parkville, Victoria, Austral | Collaborator | |
| Teddy Y. | Wu | | PhD | The Royal Melbourne Hospital, University of M | Parkville, Victoria, Austral | Collaborator | |
| Darshan G. | Shan | | MBBS | The Royal Melbourne Hospital, University of M | Parkville, Victoria, Austral | Collaborator | |
| Henry | Zhao | | MBBS | The Royal Melbourne Hospital, University of M | Parkville, Victoria, Austral | Collaborator | |
| Patrick | Salvaris | | MBBS | The Royal Melbourne Hospital, University of M | Parkville, Victoria, Austral | Collaborator | |
| Fana | Alemseged | | MD | The Royal Melbourne Hospital, University of M | Parkville, Victoria, Austral | Collaborator | |

*Indicates required information. Only first name, last name, and suffix will appear in PubMed.

| *First Name and Middle Initial(s) | *Last Name | *Suffix (eg, Jr, III) | Academic Degrees | Institution | Location (city, state/province, country) | Role or Contribution, eg, chair, principal investigator | Group (if more than 1 Group listed in the byline) and/or Subgroup (eg, Steering Committee) |
|---|---|---|---|---|---|---|---|
| Felix C. | Ng | | MBBS | The Royal Melbourne Hospital, University of M | Parkville, Victoria, Austral | Collaborator | |
| Cameron | Williams | | MBBS | The Royal Melbourne Hospital, University of M | Parkville, Victoria, Austral | Collaborator | |
| Anna | Balabanski | | MBBS | The Royal Melbourne Hospital, University of M | Parkville, Victoria, Austral | Collaborator | |
| Angela | dos Santos | | MBBS | The Royal Melbourne Hospital, University of M | Parkville, Victoria, Austral | Collaborator | |
| Jo-Lyn | Ng | | MBBS | The Royal Melbourne Hospital, University of M | Parkville, Victoria, Austral | Collaborator | |
| Amy | McDonald | | BN | The Royal Melbourne Hospital, University of M | Parkville, Victoria, Austral | Collaborator | |
| David | Jackson | | BN | The Royal Melbourne Hospital, University of M | Parkville, Victoria, Austral | Collaborator | |
| Jessica | Tsoleridis | | BN | The Royal Melbourne Hospital, University of M | Parkville, Victoria, Austral | Collaborator | |
| Lauren | Pesavento | | BN | The Royal Melbourne Hospital, University of M | Parkville, Victoria, Austral | Collaborator | |
| | | | | | | | |
| Timothy John | Kleinig | | PhD | Royal Adelaide Hospital | Port Road, Adelaide Soutl | Site PI | |
| Roy | Drew | | B Nurs | Royal Adelaide Hospital | Port Road, Adelaide Soutl | Collaborator | |
| Jennifer | Cranefield | | M Nurs | Royal Adelaide Hospital | Port Road, Adelaide Soutl | Collaborator | |
| Rebecca | Scroop | | MBBS | Royal Adelaide Hospital | Port Road, Adelaide Soutl | Collaborator | |
| Lavenia | Cagi | | MBBS | Royal Adelaide Hospital | Port Road, Adelaide Soutl | Collaborator | |
| Jackson | Harvey | | MBBS | Royal Adelaide Hospital | Port Road, Adelaide Soutl | Collaborator | |
| Michael J. | Waters | | | Royal Adelaide Hospital | Port Road, Adelaide Soutl | Collaborator | |
| | | | | | | | |
| John | Thornton | | MD | Beaumont Hospital | Dublin, Ireland | Site PI | |
| Robert P. | Brennan | | MD RCSI | Beaumont Hospital | Dublin, Ireland | Collaborator | |
| Alan | O'Hare | | MD | Beaumont Hospital | Dublin, Ireland | Collaborator | |
| Sarah | Power | | PhD | Beaumont Hospital | Dublin, Ireland | Collaborator | |
| David JP | Williams | | PhD RCSI | Beaumont Hospital | Dublin, Ireland | Collaborator | |
| Karl | Boyle | | MD | Beaumont Hospital | Dublin, Ireland | Collaborator | |
| Barry J. | Moynihan | | MD RCSI | Beaumont Hospital | Dublin, Ireland | Collaborator | |
| Ann-Marie | Liddy | | MRCPI, PhD | Beaumont Hospital | Dublin, Ireland | Collaborator | |
| Margaret | Large | | Bsc, RCSI | Beaumont Hospital | Dublin, Ireland | Collaborator | |
| Ailbhe | Cullen | | RGN, RSCN, MSc RCSI | Beaumont Hospital | Dublin, Ireland | Collaborator | |
| Roisin | Walsh | | RGN, Hdip, Pgrad, MSc RCSI | Beaumont Hospital | Dublin, Ireland | Collaborator | |
| Emma | Martin | | Bsc, Pgdip | Beaumont Hospital | Dublin, Ireland | Collaborator | |
| Julie | Lynch | | RGN, MSc | Beaumont Hospital | Dublin, Ireland | Collaborator | |
| Sinéad | McElroy | | | Beaumont Hospital | Dublin, Ireland | Collaborator | |
| | | | | | | | |

*Indicates required information. Only first name, last name, and suffix will appear in PubMed.

| ***First Name and Middle Initial(s)** | ***Last Name** | ***Suffix (eg, Jr, III)** | Academic Degrees | Institution | Location (city, state/province, country) | Role or Contribution, eg, chair, principal investigator | Group (if more than 1 Group listed in the byline) and/or Subgroup (eg, Steering Committee) |
|---|---|---|---|---|---|---|---|
| Seán | Murphy | | MD | Mater Misericordiae University Hospital | Dublin, Ireland | Site PI | |
| Sarah A. | Coveney | | MRCPI | Mater Misericordiae University Hospital | Dublin, Ireland | Collaborator | |
| Caroline | Deegan | | | Mater Misericordiae University Hospital | Dublin, Ireland | Collaborator | |
| Gillian | Horgan | | BSc | Mater Misericordiae University Hospital | Dublin, Ireland | Collaborator | |
| Peter | Kelly | | MD | Mater Misericordiae University Hospital | Dublin, Ireland | Collaborator | |
| Aoife | Laffan | | MD | Mater Misericordiae University Hospital | Dublin, Ireland | Collaborator | |
| Yudy | Llamas-Osorio | | MD | Mater Misericordiae University Hospital | Dublin, Ireland | Collaborator | |
| Catherine T. | Lynch | | BSN | Mater Misericordiae University Hospital | Dublin, Ireland | Collaborator | |
| Peter J. | Mac Mahon | | FFR(RCSI) | Mater Misericordiae University Hospital | Dublin, Ireland | Collaborator | |
| Michael | Marnane | | MB PhD | Mater Misericordiae University Hospital | Dublin, Ireland | Collaborator | |
| John J. | McCabe | | MRCPI | Mater Misericordiae University Hospital | Dublin, Ireland | Collaborator | |
| | | | | | | | |
| Paul A. | Burns | | MB | Royal Victoria Hospital | Belfast, Northern Ireland | Site PI | |
| Suzanne | Tauro | | | Royal Victoria Hospital | Belfast, Northern Ireland | Collaborator | |
| Sarah | Cuddy | | | Royal Victoria Hospital | Belfast, Northern Ireland | Collaborator | |
| Ian | Rennie | | | Royal Victoria Hospital | Belfast, Northern Ireland | Collaborator | |
| Graham | Smyth | | | Royal Victoria Hospital | Belfast, Northern Ireland | Collaborator | |
| Peter | Flynn | | | Royal Victoria Hospital | Belfast, Northern Ireland | Collaborator | |
| Ivan | Wiggam | | | Royal Victoria Hospital | Belfast, Northern Ireland | Collaborator | |
| Enda | Kerr | | | Royal Victoria Hospital | Belfast, Northern Ireland | Collaborator | |
| Patricia | Gordon | | | Royal Victoria Hospital | Belfast, Northern Ireland | Collaborator | |
| Patricia | Fearon | | | Royal Victoria Hospital | Belfast, Northern Ireland | Collaborator | |
| Geralt | Roberts | | | Royal Victoria Hospital | Belfast, Northern Ireland | Collaborator | |
| Cathy | Patterson | | | Royal Victoria Hospital | Belfast, Northern Ireland | Collaborator | |

*Indicates required information. Only first name, last name, and suffix will appear in PubMed.

| *First Name and Middle Initial(s) | *Last Name | *Suffix (eg, Jr, III) | Academic Degrees | Institution | Location (city, state/province, country) | Role or Contribution, eg, chair, principal investigator | Group (if more than 1 Group listed in the byline) and/or Subgroup (eg, Steering Committee) |
|---|---|---|---|---|---|---|---|
| Karen | Adams | | | Royal Victoria Hospital | Belfast, Northern Ireland | Collaborator | |
| Brian | Wells | | | Royal Victoria Hospital | Belfast, Northern Ireland | Collaborator | |
| Margaret | McFarland | | | Royal Victoria Hospital | Belfast, Northern Ireland | Collaborator | |
| | | | | | | | |
| Tiago | Moreira | | MD, PhD | Department of Clinical Neuroscience, Karolinsk | Stockholm, Sweden | Collaborator | |
| Christina | Sjöstrand | | MD, PhD | Department of Clinical Neuroscience, Karolinsk | Stockholm, Sweden | Collaborator | |
| Michael | Mazya | | MD, PhD | Department of Clinical Neuroscience, Karolinsk | Stockholm, Sweden | Collaborator | |
| Håkan | Almqvist | | MD | Department of Clinical Neuroscience, Karolinsk | Stockholm, Sweden | Collaborator | |
| Nils | Wahlgren | | MD, PhD | Department of Clinical Neuroscience, Karolinsk | Stockholm, Sweden | Collaborator | |
| Anna | Steinberg | | MD, PhD | Department of Clinical Neuroscience, Karolinsk | Stockholm, Sweden | Collaborator | |
| Charith | Cooray | | MD, PhD | Department of Clinical Neuroscience, Karolinsk | Stockholm, Sweden | Collaborator | |
| Einar | Eriksson | | MD, PhD | Department of Clinical Neuroscience, Karolinsk | Stockholm, Sweden | Collaborator | |
| Magnus | Thorén | | MD, PhD | Department of Clinical Neuroscience, Karolinsk | Stockholm, Sweden | Collaborator | |
| Boris | Keselman | | MD, PhD | Department of Clinical Neuroscience, Karolinsk | Stockholm, Sweden | Collaborator | |
| Niaz | Ahmed | | MD, PhD | Department of Clinical Neuroscience, Karolinsk | Stockholm, Sweden | Collaborator | |
| Åke | Holmberg | | | Department of Clinical Neuroscience, Karolinsk | Stockholm, Sweden | Collaborator | |
| Maria C. | Axelsson | | | Department of Clinical Neuroscience, Karolinsk | Stockholm, Sweden | Collaborator | |
| Annika | Berglund | | PhD | Department of Clinical Neuroscience, Karolinsk | Stockholm, Sweden | Collaborator | |
| | | | | | | | |
| Amir | Golsari | | MD | University Medical Center Hamburg-Eppendorf | Hamburg, Germany | Collaborator | |
| Julia | Hoppe | | MD | University Medical Center Hamburg-Eppendorf | Hamburg, Germany | Collaborator | |
| Milani | Deb-Chatterji | | MD | University Medical Center Hamburg-Eppendorf | Hamburg, Germany | Collaborator | |
| Bastian | Cheng | | MD | University Medical Center Hamburg-Eppendorf | Hamburg, Germany | Collaborator | |
| Ewgenia | Barow | | MD | University Medical Center Hamburg-Eppendorf | Hamburg, Germany | Collaborator | |
| Eckhard | Schlemm | | MD | University Medical Center Hamburg-Eppendorf | Hamburg, Germany | Collaborator | |
| Iris | Lettow | | MD | University Medical Center Hamburg-Eppendorf | Hamburg, Germany | Collaborator | |
| Märit | Jensen | | MD | University Medical Center Hamburg-Eppendorf | Hamburg, Germany | Collaborator | |
| Dagmar | Otto | | | University Medical Center Hamburg-Eppendorf | Hamburg, Germany | Collaborator | |
| Kirsten | Jaramillo | | | University Medical Center Hamburg-Eppendorf | Hamburg, Germany | Collaborator | |
| Hannes | Appelbohm | | | University Medical Center Hamburg-Eppendorf | Hamburg, Germany | Collaborator | |
| Jens | Fiehler | | MD | University Medical Center Hamburg-Eppendorf | Hamburg, Germany | Collaborator | |
| Maxim | Bester | | MD | University Medical Center Hamburg-Eppendorf | Hamburg, Germany | Collaborator | |
| Michael | Schönfeld | | MD | University Medical Center Hamburg-Eppendorf | Hamburg, Germany | Collaborator | |

*Indicates required information. Only first name, last name, and suffix will appear in PubMed.

| *First Name and Middle Initial(s) | *Last Name | *Suffix (eg, Jr, III) | Academic Degrees | Institution | Location (city, state/province, country) | Role or Contribution, eg, chair, principal investigator | Group (if more than 1 Group listed in the byline) and/or Subgroup (eg, Steering Committee) |
|---|---|---|---|---|---|---|---|
| Caspar | Brekenfeld | | MD | University Medical Center Hamburg-Eppendorf | Hamburg, Germany | Collaborator | |
| Brigette | Holst | | MD | University Medical Center Hamburg-Eppendorf | Hamburg, Germany | Collaborator | |
| Ginette | Wortmann | | | University Medical Center Hamburg-Eppendorf | Hamburg, Germany | Collaborator | |
| Friederike | Koch | | | University Medical Center Hamburg-Eppendorf | Hamburg, Germany | Collaborator | |
| | | | | | | | |
| Johannes C. | Gerber | | MD | University Hospital Carl Gustav Carus at the Te | Dresden, Germany | Collaborator | |
| Andrij | Abramyuk | | MD | University Hospital Carl Gustav Carus at the Te | Dresden, Germany | Collaborator | |
| Daniel | Kaiser | | MD | University Hospital Carl Gustav Carus at the Te | Dresden, Germany | Collaborator | |
| Simon | Winzer | | MD | University Hospital Carl Gustav Carus at the Te | Dresden, Germany | Collaborator | |
| Alexandra | Prakapenia | | MD | University Hospital Carl Gustav Carus at the Te | Dresden, Germany | Collaborator | |
| Lars-Peder | Pallesen | | MD | University Hospital Carl Gustav Carus at the Te | Dresden, Germany | Collaborator | |
| Timo | Siepmann | | MD | University Hospital Carl Gustav Carus at the Te | Dresden, Germany | Collaborator | |
| Kristian | Barlinn | | MD | University Hospital Carl Gustav Carus at the Te | Dresden, Germany | Collaborator | |
| Kathrin | Haase | | RN | University Hospital Carl Gustav Carus at the Te | Dresden, Germany | Collaborator | |
| Angela | Sauer | | RN | University Hospital Carl Gustav Carus at the Te | Dresden, Germany | Collaborator | |
| | | | | | | | |
| Marios-Nikos | Psychogios | | MD | University Medical Center Göttingen | Göttingen, Germany | Site PI | |
| Jan | Liman | | MD | University Medical Center Göttingen | Göttingen, Germany | Collaborator | |
| Alex | Brehm | | MD | University Medical Center Göttingen | Göttingen, Germany | Collaborator | |
| Volker | Maus | | MD | University Medical Center Göttingen | Göttingen, Germany | Collaborator | |
| Nicole | Hollstein | | | University Medical Center Göttingen | Göttingen, Germany | Collaborator | |
| Annika | Reinke | | | University Medical Center Göttingen | Göttingen, Germany | Collaborator | |
| Gustav | Neitz | | | University Medical Center Göttingen | Göttingen, Germany | Collaborator | |
| Marlena | Schnieder | | MD | University Medical Center Göttingen | Göttingen, Germany | Collaborator | |
| Margret | Schwarz | | | University Medical Center Göttingen | Göttingen, Germany | Collaborator | |
| Allam | Ibrahim | | MD | University Medical Center Göttingen | Göttingen, Germany | Collaborator | |
| Julia | Müller | | | University Medical Center Göttingen | Göttingen, Germany | Collaborator | |
| Stefanie | Meister | | | University Medical Center Göttingen | Göttingen, Germany | Collaborator | |
| Berit | Bäumle | | | University Medical Center Göttingen | Göttingen, Germany | Collaborator | |
| | | | | | | | |
| René | Chapot | | MD | Alfred Krupp Krankenhaus Hospital, Departme | Essen, Germany | Site PI | |
| Sara | Pilgram-Pastor | | MD | Alfred Krupp Krankenhaus Hospital, Departme | Essen, Germany | Collaborator | |
| Marta | Wallocha | | MD | Alfred Krupp Krankenhaus Hospital, Departme | Essen, Germany | Collaborator | |
| Michael | Stauder | | MD | Alfred Krupp Krankenhaus Hospital, Departme | Essen, Germany | Collaborator | |
| Ekin | Celik | | MD | Alfred Krupp Krankenhaus Hospital, Departme | Essen, Germany | Collaborator | |
| Paul | Stracke | | MD | Alfred Krupp Krankenhaus Hospital, Departme | Essen, Germany | Collaborator | |

Supplemental Online Content: Nonauthor Collaborators

*Indicates required information. Only first name, last name, and suffix will appear in PubMed.

| ***First Name and Middle Initial(s)** | ***Last Name** | ***Suffix (eg, Jr, III)** | Academic Degrees | Institution | Location (city, state/province, country) | Role or Contribution, eg, chair, principal investigator | Group (if more than 1 Group listed in the byline) and/or Subgroup (eg, Steering Committee) |
|---|---|---|---|---|---|---|---|
| Nicole | Müller | | | Alfred Krupp Krankenhaus Hospital, Departmer | Essen, Germany | Collaborator | |
| Ralph | Weber | | MD | Alfred Krupp Krankenhaus Hospital, Departmer | Essen, Germany | Collaborator | |
| R. | Veltkamp | | MD | Alfred Krupp Krankenhaus Hospital, Departmer | Essen, Germany | Collaborator | |
| | | | | | | | |
| Simon | Nagel | | MD | University Hospital Heidelberg | Heidelberg, Germany | Site PI | |
| Peter Arthur | Ringleb | | MD | University Hospital Heidelberg | Heidelberg, Germany | Collaborator | |
| Miriam | Heyse | | MD | University Hospital Heidelberg | Heidelberg, Germany | Collaborator | |
| Hemasse | Amiri | | MD | University Hospital Heidelberg | Heidelberg, Germany | Collaborator | |
| Sibu | Mundiyanapurath | | MD | University Hospital Heidelberg | Heidelberg, Germany | Collaborator | |
| Min | Chen | | MD | University Hospital Heidelberg | Heidelberg, Germany | Collaborator | |
| Elisabeth | Beyrle | | | University Hospital Heidelberg | Heidelberg, Germany | Collaborator | |
| Perdita | Beck | | | University Hospital Heidelberg | Heidelberg, Germany | Collaborator | |
| Markus Alfred | Möhlenbruch | | MD | University Hospital Heidelberg | Heidelberg, Germany | Collaborator | |
